# Reactivity of [{Cp'Fe(μ‐I)}_2_] toward 3‐phospha‐ and 3‐arsaethynolate

**DOI:** 10.1002/chem.202501339

**Published:** 2025-07-04

**Authors:** Katharina Münster, Jasper Mindner, William‐Dale Möller, Dirk Baabe, Iker del Rosal, Laurent Maron, Marc D. Walter

**Affiliations:** ^1^ Institut für Anorganische und Analytische Chemie Technische Universität Braunschweig Hagenring 30 38106 Braunschweig Germany; ^2^ Université de Toulouse et CNRS, INSA, UPS, UMR5215 LPCNO135 Avenue de Rangueil Toulouse 31077 France

**Keywords:** arsenic, cyclopentadienyl, iron, mössbauer spectroscopy, phosphorus

## Abstract

[{Cp'Fe(μ‐I)}_2_] (**I**; Cp’ = η^5^–1,2,4‐(Me_3_C)_3_C_5_H_2_) reacts with [Na(OCP)(1,4‐dioxane)_x_] and K(OCAs) to yield [(Cp'Fe)_2_(μ‐η^2^:η^2^‐P_2_)(μ‐CO)] (**1**) and [(Cp'Fe)_2_(μ‐η^2^:η^2^‐As_2_)(μ‐CO)] (**3**), respectively. While complex **1** was previously accessed by *Scherer* and co‐workers using rather harsh reaction conditions, the new synthetic method already proceeds at ambient temperature. UV‐light irradiation of **1** and **3** induces CO release forming complexes [(Cp'Fe)_2_(μ‐η^2^:η^2^‐E_2_)] (E = P (**2**) and As (**4**)), respectively. However, both reactions also yielded several byproducts which were spectroscopically identified. Furthermore, upon thermally triggered CO elimination from **3** the Fe_3_As_6_‐cluster [(Cp'Fe)_3_(As_3_)_2_] (**5**) is isolated in low yield. In addition, zero‐field ^57^Fe Mössbauer spectra were recorded on complexes **1**–**4** and computational studies complement the experimental findings and provide additional insights into the bonding in these complexes and the reaction pathways resulting in the formation of complexes **1**–**4**.

## Graphical abstract

[(Cp'Fe)_2_(μ‐η^2^:η^2^‐E_2_)(μ‐CO)] (E = P, As) can be isolated from the reaction of [{Cp'Fe(μ‐I)}_2_] (Cp’ = η^5^‐1,2,4‐(Me_3_C)_3_C_5_H_2_) with [Na(OCP)(1,4‐dioxane)_x_] and K(OCAs), respectively. UV‐light induced CO elimination yields [(Cp'Fe)_2_(μ‐η^2^:η^2^‐E_2_)] (E = P and As). ^57^Fe Mössbauer spectra as well as computational studies were performed.

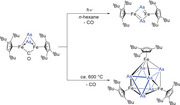

## 1 Introduction

**Scheme 1:**
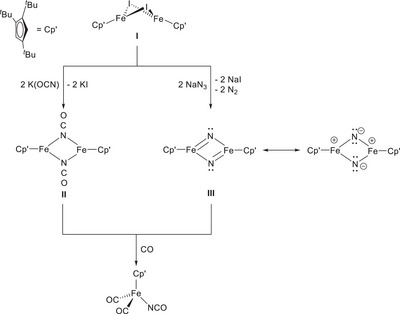
Reactivity of pseudo‐halides toward [{Cp'Fe(μ‐I)}_2_] (**I**).

In earlier investigations, the iron half‐sandwich complex [{Cp'Fe(μ‐I)}_2_] (**I**; Cp’ = η^5^–1,2,4‐(Me_3_C)_3_C_5_H_2_) was found to react with pseudo‐halides such as KOCN and NaN_3_ to form the cyanato‐ or nitrido‐bridged complexes **II** and **III**, respectively (Scheme 1).^[^ ^1^ ^]^ The reaction with KOCN proceeds via a straightforward salt metathesis, whereas the NaN_3_‐mediated reaction leads to complex **III** through spontaneous N_2_ elimination that precludes the isolation of an azido‐bridged intermediate. Despite the importance of nitrido complexes in various chemical transformations,^[^ ^2^, ^3^, ^4^ ^]^ compound **III** remains inert toward H_2_ or a range of different *N*‐heterocyclic carbenes (NHCs). Nevertheless, in the presence of CO (5 bar), it is converted into [Cp'Fe(CO)_2_(NCO)]–a product that can also be obtained by reacting **II** with CO.^[^ ^1^ ^]^

**Scheme 2:**
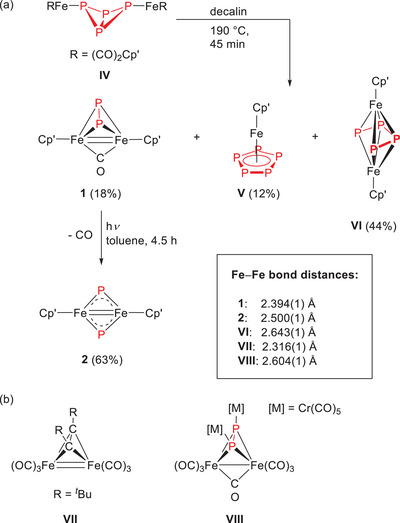
(a) Thermolysis of butterfly molecule **IV** to yield **1**, which extrudes CO on irradiation with UV light. (b) Examples of substituted iron tetrahedranes. The Fe‒Fe bonds are drawn as originally proposed in the respective publications.^[^ ^5^, ^6^, ^7^ ^].^

Over two decades ago, *Scherer* demonstrated that thermolysis of the butterfly molecule **IV** yields the diphosphido‐carbonyl‐bridged complex **1**, in addition to two phosphorus‐containing products: pentaphosphaferrocene **V** and the phosphabutadiene‐bridged triple‐decker **VI**, formed in 12% and 44% yield, respectively (Scheme 2a).^[^ ^5^ ^]^

Upon irradiation with UV‐light, complex **1** liberates carbon monoxide (CO) to form the phosphido complex **2** (Scheme 2a), which serves as the heavier homologue to compound **III**. Complex **1** is characterized by a tetrahedrane core with an Fe–Fe distance of 2.394(1) Å. For comparison, complexes **VII** ^[^ ^6^ ^]^ and **VIII** ^[^ ^7^ ^]^ feature Fe–Fe distances of 2.316(1) Å and 2.604(1) Å – values that are indicative of Fe–Fe double and single bonds, respectively (Scheme 2b). The comparison of these values to the Fe‒Fe bond distance in **1** supports the notion of an Fe–Fe double bond. A similar inference is made for the phosphido‐bridged dimer **2**, which also features a relatively short Fe–Fe separation of 2.501(1) Å.^[^ ^5^ ^]^ However, despite the nitrido‐bridged analog **III** displaying an even shorter Fe‐Fe distance of 2.232(1) Å, computational studies suggest that no direct Fe–Fe bonding interaction is present in this case (Scheme 1),^[^ ^1^ ^]^ and that the bond order is not necessarily related to the Fe‒Fe distance. Another example constitutes [{Cp'Fe(μ‐CPh)}_2_], in which–despite an Fe···Fe distance of 2.3558(4) Å–direct Fe‐Fe bonding was discounted. In this case, DFT computations suggest that the electronic structure is best described as two low‐spin Fe(II) [Cp'Fe]^+^ cations being bridged by two monoanionic PhC^−^ ligands representing a deprotonated benzylidene fragment with six valence electrons.^[8]^

**Scheme 3:**
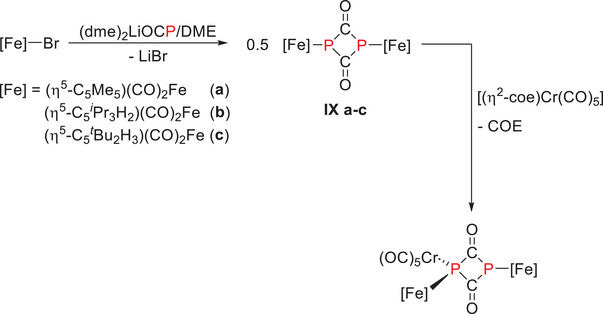
Synthesis of 1,3‐diferrio‐1,3‐diphosphetane‐2,4‐diones [{(η^5^‐C_5_R_5_)(CO)_2_Fe}_2_{μ‐(PCO)}_2_] (C_5_R_5_ = C_5_Me_5_, 1,3‐*^t^* Bu_2_C_5_H_3_, 1,2,4‐*^i^* Pr_3_C_5_H_2_) (**IXa‐c**).^[^ ^9^ ^].^

In 1996, *Weber* detailed the synthesis of 1,3‐diferrio‐1,3‐diphosphetane‐2,4‐diones [{(η^5^‐C_5_R_5_)(CO)_2_Fe}_2_{μ‐(PCO)}_2_] (C_5_R_5_ = C_5_Me_5_, 1,3‐*^t^* Bu_2_C_5_H_3_, 1,2,4‐*^i^* Pr_3_C_5_H_2_) by reacting [(η^5^‐C_5_R_5_)(CO)_2_FeBr] with lithium 3‐phosphaethynolate ((dme)_2_LiOCP) in 1,2‐dimethoxyethane (DME). The only documented reactivity involved its reaction with [(η^2^‐coe)Cr(CO)_5_] (Scheme 3).^[^ ^9^ ^]^

The 3‐phosphaethynolate anion OCP^−^ was initially prepared by *Becker* in 1992,^[^ ^10^ ^]^ but along with its arsenic analog, OCAs^−^, it has only recently been re‐discovered as a valuable reagent. This renewed interest stems from the work of *Grützmacher* and *Goicoechea*,^[^ ^11^ ^]^ who established efficient synthetic protocols for [Na(OCP)(1,4‐dioxane)_x_]^[^ ^12^ ^]^ and K(OCAs),^[^ ^13^, ^14^, ^15^ ^]^ respectively. These species can act as atom transfer reagents for P and As–releasing CO during the process–which inspired computational studies to assess whether OCP^−^ could serve as a synthetic entry point for phosphido‐bridged species such as **III**.^[^ ^16^ ^]^

Building on this precedent, we investigated the reactivity of [{Cp'Fe(μ‐I)}_2_] (**I**) toward OCE^−^ (E = P, As) with the dual goal of uncovering a more efficient route to [(Cp'Fe)_2_(μ‐η^2^:η^2^‐P_2_)] (**II**) and of accessing its congener [(Cp'Fe)_2_(μ‐η^2^:η^2^‐As_2_)]. This contribution details our efforts in synthesizing and characterizing these molecules.

## 2 Results and Discussion

### 2.1 Syntheses

**Scheme 4:**
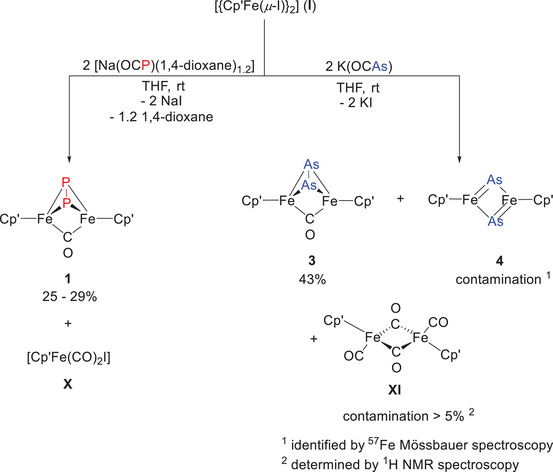
Synthesis of diphospha‐ and diarsadiferratetrahedranes **1** and **3**. The presence of **4** was initially suspected based on ^57^Fe Mössbauer spectra of **3**. However, the unavailability of pure **4**, combined with the complications arising from overlapping NMR signals, precluded further validation. Considering the ambiguity with respect to the presence of an Fe‒Fe bond we refrained from drawing such bonds in this Scheme.

When [{Cp'Fe(μ‐I)}_2_] (**I**) is treated with [Na(OCP)(1,4‐dioxane)_x_], the reaction affords a mixture containing [(Cp'Fe)_2_(μ‐η^2^:η^2^‐P_2_)(μ‐CO)] (**1**) together with the by‐product [Cp'Fe(CO)_2_I] (**X**) (Scheme 4). Despite thorough investigations under varying reaction conditions, the product distribution remained unchanged. Notably, even under a 6.5 bar CO atmosphere, no 1,3‐diphosphetane‐2,4‐dione‐bridged species analogous to **IX** is observed. Owing to the nearly identical solubility of the two products **1** and **X**, the reaction mixture must be purified by preparative thin‐layer chromatography or column chromatography under inert conditions to isolate pure [(Cp'Fe)_2_(μ‐η^2^:η^2^‐P_2_)(μ‐CO)] (**1**) in yields of 25–29%.

The reaction of [{Cp'Fe(μ‐I)}_2_] with K(OCAs) in Et_2_O affords [(Cp'Fe)_2_(μ‐η^2^:η^2^‐As_2_)(μ‐CO)] (**3**) in 43% yield (Scheme 4). Much like its phosphorus analog **1**, column chromatography under inert conditions is required to remove byproducts such as [{Cp'Fe(CO)}_2_(μ‐CO)_2_] (**XI**) and [(Cp'Fe)_2_(μ‐η^2^:η^2^‐As_2_)] (**4**). While most of **XI** can be removed by multiple purification cycles as shown by Mössbauer spectroscopy (vide infra). These side products were identified either by ^1^H NMR or ^57^Fe Mössbauer spectroscopy (vide infra). The green, micro‐crystalline compounds **1** and **3** are both moisture‐ and air‐sensitive, yet stable at room temperature under inert conditions, exhibiting melting points of 242 °C (with decomposition near 280 °C) and 225 °C, respectively.

**Scheme 5:**
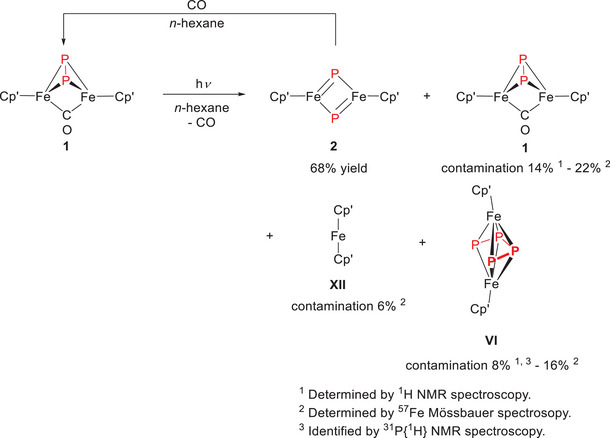
Synthesis of [(Cp'Fe)_2_(μ‐η^2^:η^2^‐P_2_)] (**2**) by UV‐light‐driven CO extrusion. Considering the ambiguity with respect to the presence of an Fe‒Fe bond we refrained from drawing such bonds in this Scheme.

Following isolation of the CO‐bridged complexes, photochemical CO elimination was undertaken by irradiating *n*‐hexane solutions of **1** with UV light – similar to the procedure described by *Scherer* ^[^ ^5^ ^]^ – but employing a slight vacuum to assist in CO release (Scheme 5). This procedure proved most effective on small scales (approximately 10 mg of starting material per batch). Nevertheless, ^1^H NMR and ^57^Fe Mössbauer spectra revealed that up to 22% of the starting material **1** might still be present, along with additional contaminants such as ferrocene **XII** (up to 8%) and the P_4_‐bridged Fe complex **VI** (up to 14%). Despite efforts to purify the product further, the complete removal of these impurities proved elusive. Moreover, extended irradiation is discouraged due to the risk of forming new, unidentified degradation products.

**Scheme 6:**
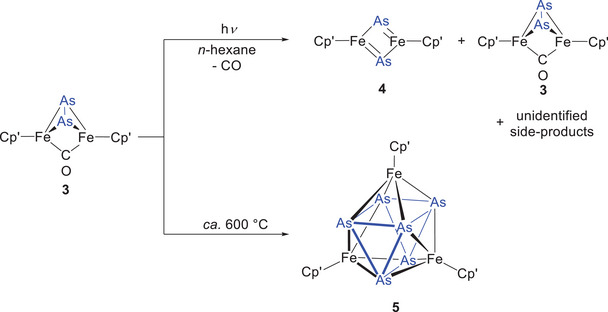
Attempted synthesis of [(Cp'Fe)_2_(μ‐η^2^:η^2^‐As_2_)] (**4**) by UV‐light‐driven CO extrusion. Considering the ambiguity with respect to the presence of an Fe‒Fe bond we refrained from drawing such bonds in this Scheme.

**Figure 1:**
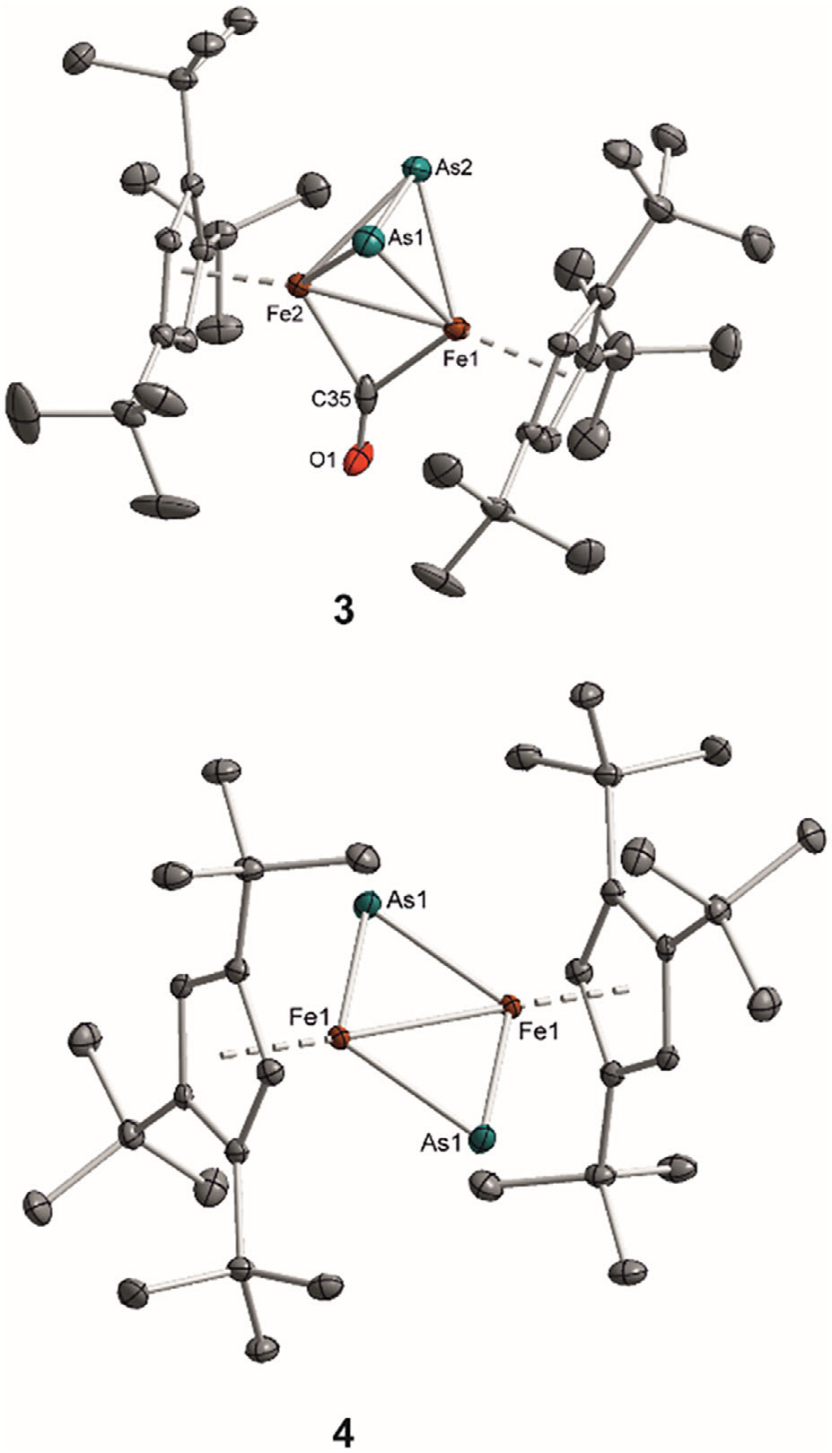
Diamond^[^ ^35^ ^]^ representations of the solid‐state molecular structures for complexes **3** and **4**. Thermal ellipsoids are drawn at the 50% probability level. Hydrogen atoms are omitted for clarity.

For the arsenic congener **3**, CO extrusion under UV‐light irradiation proves even more challenging. Irrespective of the employed solvent (THF, Et_2_O, or *n*‐hexane), the reaction yields a complex mixture comprised of [(Cp'Fe)_2_(μ‐η^2^:η^2^‐As_2_)] (**4**), unreacted starting material **3** and other as‐yet unidentified products (Scheme 6). Despite these difficulties in optimizing reaction conditions and purifying the resulting mixture, a few crystals of **4** suitable for X‐ray diffraction analysis were ultimately obtained. The molecular structures of **3** and **4** are depicted in Figure 1.

Furthermore, efforts to trigger CO release by heating compound **3** in the solid state to approximately 600 °C under a dinitrogen atmosphere, followed by evacuation of the reaction flask, resulted in the isolation of a few crystals of the Fe_3_As_6_ cluster **5** (Scheme 6, and see Supporting Information for details), which features a known structural motif previously observed for [(Cp^BIG^Fe)_3_(μ_3_,η^4:4:4^‐As_6_)] (Cp^BIG^ = η^5^‐C_5_(4‐*n*BuC_6_H_4_)_5_),^[^ ^17^ ^]^ [(Cp*Fe)_3_As_6_]^+^ (Cp* = η^5^‐C_5_Me_5_),^[^ ^18^ ^]^ [(Cp*Fe)_3_As_6_]^x^ (Cp* = η^5^‐C_5_Me_5_; x = 2+, 1+, 0, 1‐),^[^ ^19^ ^]^ and [(Cp’’Fe)_3_As_6_]^x^ (Cp’’ = η^5^–1,3‐*^t^* Bu_2_C_5_H_3_). ^[^ ^20^ ^]^

### 2.2 NMR Spectroscopy

Compounds **1**, **2**, and **3** were characterized using ^1^H and ^13^C{^1^H} spectroscopy in C_6_D_6_ solution at ambient temperature, and their chemical shifts are summarized in Table 1. Notably, the ^31^P{^1^H} NMR spectra of the phosphorus‐containing complexes **1** and **2** exhibit markedly low‐field‐shifted resonances at δ = 780.3 and 1407.1 ppm, respectively, in excellent agreement with the previously reported values.^[^ ^5^ ^]^

**Table 1:** ^1^H and ^31^P{^1^H} NMR chemical shifts (*δ* in ppm) of complexes **III**, **1**, **2,** and **3** recorded in C_6_D_6_ at room temperature.

Compound	2 x *t*Bu^[^ ^a^ ^]^	4 x *t*Bu^[^ ^a^ ^]^	Cp’‐*H* ^[a]^	^31^P{^1^H} NMR	^15^N{^1^H} NMR
**III** ^[b]^	0.58	1.33	6.45	–	1118
**1**	1.10	1.27	6.02	780.3	–
**2**	0.33	1.33	6.55	1407.1	–
**3**	2.60	1.87	10.48	–	–

^[a]^ ^1^H NMR.

^[b]^ Ref. 1]

To contextualize these values, they can be directly compared to those reported for related compounds, e.g. [(η^2^‐P_2_)Fe(CO)_2_(CNAr^Dipp2^)_2_] (Ar^Dipp2^ = 2,6‐(2,6‐(*iso*‐propyl)_3_C_6_H_2_)_2_C_6_H_3_)) (δ(^31^P) = 434.4 ppm), ^[^ ^21^ ^]^ [{CpFe(CO)_2_}_3_(μ_3_‐P)] (δ(^31^P) = 373.9 ppm), [{CpFe(CO)_2_}{CpFe(CO)}_2_(μ‐CO)(P)] (δ(^31^P) = 450.3 ppm), [{CpFe(CO)_2_}{CpFe(CO)}_2_(μ‐CO)(P‐Cl)]^+^ (δ(^31^P) = 492.6 ppm),^[^ ^22^ ^]^ [{CpFe(CO)_3_}_3_(P‐L)_2_] (L = transition metal‐carbonyl fragment, δ(^31^P) > 700 ppm)^[^ ^7^ ^]^ or [{(W(OAr)_2_)_2_(μ‐P)}(μ‐P‐C*^t^* Bu){μ‐P(OAr)_2_}] (δ(^31^P) = 831.8 ppm for the “naked” P atom),^[^ ^23^ ^]^ and phosphinidene complexes (δ(^31^P) in the range of ca. 790 to 1,200 ppm).^[^ ^24^, ^25^ ^]^ The ^31^P{^1^H} NMR chemical shift in **1** can further be related to that experimentally determined for P_4_ (δ(^31^P) = −540 ppm)^[^ ^26^ ^]^ and that computed by density functional theory (DFT) methods for free P_2_ (δ_calc_(^31^P) = 689.3 ± 45.4 ppm).^[^ ^27^, ^28^, ^29^ ^]^

For the CO elimination product **2**, a minor phosphorus‐containing species was detected in the ^31^P{^1^H} NMR spectrum as a broad singlet δ = 91.3 ppm. It corresponds to the P_4_‐bridged compound **VI** ^[^ ^30^ ^]^ and is present by approximately 8% as determined by ^1^H NMR spectroscopy. The Cp’‐ring C*H* resonances are observed at δ = 6.02 ppm for **1** and δ = 6.55 ppm for **2**, whereas for **3** the corresponding resonance is shifted considerably downfield to δ = 10.48 ppm. Additionally, the *tert*‐butyl group in the 3‐position experiences a substantial upfield shift from δ = 1.10 ppm in **1** to δ = 0.33 ppm in **2**. Interestingly, the ^1^H NMR chemical shifts for **2** bear striking resemblance to those of the previously reported nitrido counterpart **III**.^[^ ^1^ ^]^ Further notable features emerge in the ^13^C{^1^H} NMR spectra. The resonance for the Cp’‐ring *C*H groups is found at δ = 81.6 ppm in the compounds of **1** and **2** but shifts significantly to higher field (δ = 45.9 ppm) for **3**. In contrast, the methyl groups of the *tert*‐butyl group in 3‐position in complex **3** are markedly deshielded, resonating at δ = 81.0 ppm, whereas in **1** and **2** these resonances appear at δ = 38.4 and 34.2 ppm, respectively. The unusual chemical shifts for **3** might be linked to energetically low‐lying excited states, affecting the chemical shift (see Computational section).

### 2.3 X‐Ray Crystallography

Compounds **3** and **4** were crystallized from saturated *n*‐hexane solutions at ‒30 °C. The solid‐state molecular structures are illustrated in Figure 1, and selected bond distances and angles are summarized in Table 2. For ease of comparison, Table 2 also includes the corresponding values reported for **III**,^[^ ^1^ ^]^ **1** and **2**.^[^ ^5^ ^]^

**Table 2:** Selected bond distances (Å) and angles (°) for the pnictogen‐bridged diiron complexes **1**–**4** and **III**.

	1 ^[a]^	3	III ^[b]^	2 ^[a]^	4
E	P	As	N	P	As
**Cp_cent_‐Fe**	1.746(1), 1.742(1)	1.734(1), 1.739(1)	1.744(1), 1.744(1)	1.728(1), 1.727(1)	1.728(1)
**Fe···Fe**	2.394(1)	2.410(1)	2.232(1)	2.501(1)	2.567(1)
**E···E/E‐E**	2.064(2)	2.288(1)	2.667(3)	3.383(1)	3.618(1)
**Fe‐E (mean)**	2.291(2)	2.397(1)	1.739(2)	2.104(1)	2.218(1)
**Fe‐CO**	1.922(7) 1.914(7)	1.937(4) 1.941(3)	–	–	–
**angle between Cp’ planes**	16.7(2)	17.5(2)	4.1(2)	2.3(2)	0
**angle Cp'Fe/Cp'Fe**	14.5(1)	15.1(1)	3.0(1)	0.9(2)	0

^[a]^ Ref. 5]

^[b]^ Ref. [1]

All complexes feature Cp_cent_‒Fe distances that fall within a narrow range, spanning from 1.727(1) to 1.746(1) Å. The two isostructural complexes **1** and **3**, which both crystallize in the space group *P*2_1_/n, exhibit E‒E distances in the μ‐η^2^:η^2^‐E_2_ moiety of 2.064(2) for E = P and 2.288(1) Å for E = As. These bond lengths are in excellent agreement with values reported for other μ‐η^2^:η^2^‐E_2_‐bridged complexes. A comprehensive review by *Scherer* documents numerous μ‐η^2^:η^2^‐E_2_‐bridged compounds available up to 1989,^[^ ^31^ ^]^ and more recent examples include [(Cp*Cr(CO)_2_)_2_(μ‐η^2^:η^2^‐P_2_)] (2.060(1) Å),^[^ ^32^ ^]^ [(LFe)_2_(μ‐η^2^:η^2^‐P_2_)_2_] (L = CH(CHNDipp)_2_, Dipp = 2,6‐*^i^* Pr_2_C_6_H_3_) (2.036(2) Å), ^[^ ^33^ ^]^ [(η^2^‐P_2_)Fe(CO)_2_(CNAr^Dipp2^)_2_] (Ar^Dipp2^ = 2,6‐(2,6‐(*iso*‐propyl)_3_C_6_H_2_)_2_C_6_H_3_)) (1.987(3)/1.989(3) Å),^[^ ^21^ ^]^ and [(L^B^Fe)_2_(μ‐η^2^:η^2^‐As_2_)] (L^B^ = CH(C*^t^* BuNDipp)_2_, Dipp = 2,6‐*^i^* Pr_2_C_6_H_3_) (2.3447(5) Å).^[^ ^34^ ^]^

Analyzing the E‒E bond distances provides valuable insights into the nature of the μ‐η^2^:η^2^‐E_2_ moiety. For instance, gas phase electron diffraction studies on E_4_ reveal E‒E single bond distances of 2.1994(3) Å for P_4_^[^ ^36^ ^]^ and 2.435(4) Å for As_4_. ^[^ ^37^ ^]^ In contrast, E = E double bonds in compounds such as [{(Me_3_Si)_3_CE}_2_] are shorter with 2.014(6)/2.004(6) Å for E = P^[^ ^38^ ^]^ and 2.245(1) Å for E = As.^[^ ^39^ ^]^ When these are compared to the significantly shorter bonds observed in free E₂ molecules − 1.893 Å for P_2_ and 2.103 Å for As_2_^[^ ^40^ ^]^ – it becomes apparent that the μ‐η^2^:η^2^‐E_2_ units exhibit characteristics most consistent with E = E double bonds. This is attributed to the synergistic interplay between σ‐donation from the E₂ π‐orbitals and π‐back‐bonding into their π*‐orbitals, which effectively reduces the bond order.^[^ ^41^ ^]^

Notably, after CO extrusion, the E‒E distances in **2** and **4** (E = P, As) increase by approximately 1.3 Å compared to those in their CO‐bridged counterparts **1** and **3** indicating E‒E bond cleavage. This is further supported by the average decrease of the Fe‒E distances by 0.19 Å for E = P and 0.18 Å for E = As.

**Figure 2:**
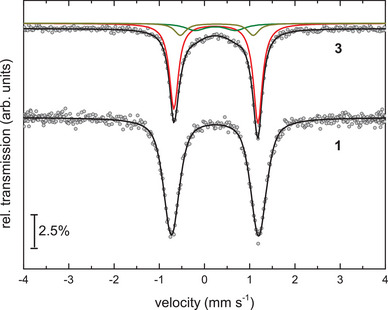
Zero‐field ^57^Fe Mössbauer spectra of **1** (at T = 80 K) and **3** (at T = 100 K). Symbols: experimental data. Solid lines: fit with doublets of Lorentzian lines. The black line represents the superposition of the different sub‐spectra (used in the fit for compound **3**), suggesting the presence of nonequivalent ^57^Fe sites in this specimen. The main signal (red) is attributed to complex **3**, while the additional components (green and dark yellow) can be associated with **4** (or, alternatively, with **4** and a yet unidentified by‐product). The parameters of the fit are summarized in Table 3.

Additionally, the Fe···Fe distances offer further insights into the bonding in these systems. In complexes **1** and **3**, the Fe···Fe separation is approximately 2.4 Å; however, upon conversion to compounds **2** and **4**, these distances expand by roughly 0.1 and 0.15 Å, respectively. In comparison, the nitrido‐bridged complex **III** features a significantly shorter Fe‒Fe distance of 2.232(1) Å.^[^ ^1^ ^]^ As a reference, literature values for Fe‒Fe single bonds typically range from 2.5 to 2.6 Å,^[^ ^42^ ^]^ whereas Fe‒Fe double bonds are considerably shorter (e.g., 2.316(1) Å in [(CH_3_)_3_CC = CC(CH_3_)_3_][Fe_2_(CO)_6_] (**VII**, Figure 2b)^[^ ^6^ ^]^ or 2.265(1) Å in [(η^5^‐C_5_Me_5_)_2_Fe_2_(μ‐CO)_3_]. ^[^ ^43^ ^]^ This comparison has led to the proposal that both complexes **1** and **2** may feature formal Fe‒Fe double bonds (Figure 2).^[^ ^5^ ^]^ Following the same argument would then also suggest the presence of a formal Fe‒Fe double bond in **3**, while the Fe···Fe distance of 2.567(1) Å in **4** would suggest a Fe‒Fe single bond. Nevertheless, for complex **III** computational studies suggest the absence of any direct Fe─Fe bonding interaction despite its shorter Fe···Fe distance. Obviously assigning metal‐metal bonds solely on bond distances can lead to incorrect assignments. This intriguing discrepancy will be examined in greater depth in the computational section (*vide infra*).

Extrusion of CO alleviates steric crowding at the Fe atoms in complexes **2** and **4**. This is evidenced by the cyclopentadienyl ring planes adopting an almost or a perfectly parallel orientation, a stark contrast to the distinctly bent arrangement observed in the CO‐containing molecules **1** and **3**.

### 2.4 Zero‐field ^57^Fe Mössbauer Spectroscopy

The zero‐field Mössbauer spectra for compounds **1** and **3** were recorded at T = 80 K or T = 100 K, respectively, and are depicted in Figure 2. Similarly, the product mixtures **2** and **4** obtained from the CO elimination were also analyzed by Mössbauer spectroscopy at T = 100 K; the corresponding spectra are provided in the Supporting Information (Figures S32 and S33). All spectra were fitted using doublets of Lorentzian lines, and Table 3 summarizes the resulting parameters alongside literature values for related compounds.

**Table 3:** Zero‐field ^57^Fe Mössbauer parameters at T = 80 or 100 K, respectively, for compounds **1** – **4**, **4 + CO**, and **XI**. The isomer shift (δ) is specified relative to metallic iron at ambient temperatures but was not corrected for the second order Doppler shift. $\Delta E_{Q}$ denotes the electric quadrupole splitting, $\Gamma_{FWHM}$ the full (Lorentzian) line width at half maximum and V the relative integral intensity of a specific sub‐spectrum used in the fit. In some cases, the ratio of the integral intensities of the individual lines ($A_{1}/A_{2}$) of a specific doublet of Lorentzian lines was also used as free parameter in the fit. The given uncertainties (in parentheses) correspond to the uncertainty of the fit.

Compound	T / K	δ / mm s^−1^	ΔE_Q_ / mm s^−1^	Γ / mm s^−1^	$A_{1}/A_{2}$ ^[a]^	V / %	Assignment
[{Cp'Fe(μ‐I)}_2_] (**I**)^[b]^	75	1.066(4)	1.973(7)	0.158(3)		100	→ **I**
[{Cp'Fe(μ‐*κ*(*N*)‐NCO)}_2_] (**II**)^[c]^	77	1.05(1)	1.49(1)			100	→ **II**
[{Cp'Fe(μ‐N)}_2_] (**III**)^[d]^	77	−0.02(1)	0.71(1)			100	→ **III**
[Cp'FeCp’’]^[d]^, ^[e]^	100	0.58(1)	2.49(1)	0.26(1)		100	**→ [Cp'FeCp’’]**
**1**	80	0.355(2)	1.928(8)	0.2913(13)	1.01(1)	100	**→ 1**
**2**	100	0.192(3)	1.150(5)	0.318(9)	1.09(2)	57.0	**→ 2**
		0.368(6)	1.864(14)	0.349(25)	1^[f]^	21.7	**→ 1**
		0.311(9)	0.780(16)	0.274(21)	1^[f]^	13.7	**→ VI**
		0.452(48)	2.539(101)	0.384(79)	1^[f]^	7.6	**→ XII**
**3**	100	0.374(1)	1.857(5)	0.241(5)	1.17(3)	75.6	**→ 3**
		0.360(30)	0.893(71)	0.540(71)	1^[f]^	12.2^[g]^	**→ 4 (?)**
		0.392(11)	1.616(50)	0.299(41)	1^[f]^	12.2^[g]^	**→ 4 (?)**
**4**	100	0.437(3)	1.634(6)	0.443(7)	0.95(2)	46.3^[g]^	→ **4 (?)**
		0.363(2)	0.865(5)	0.397(6)	1.10(2)	46.3^[g]^	**→ 4 (?)**
		0.544(6)	2.449(17)	0.248(20)	1^[f]^	7.4	**→ XII**
**4 + CO**	100	0.220(4)	1.875(4)	0.314(10)	0.86(2)	45.7	**→ XI**
		0.36^[e]^	0.87^[e]^	0.622(39)	1^[f]^	20.2^[g]^	**→ 4 (?)**
		0.44^[e]^	1.63^[e]^	0.300(13)	1^[f]^	20.2^[g]^	**→ 4 (?)**
		0.54^[e]^	2.45^[e]^	0.249(40)	1^[f]^	7.8	→ **XII**
		0.37^[e]^	1.86^[e]^	0.192*	1^[f]^	8.0	→ **3**
C_5_H_5_	77	0.23	1.90	0.27		100	
[Cp'Fe(CO)_2_(μ‐CO)_2_] (**XI**)	100	0.244(1)	1.929(1)	0.291(2)	1.07(1)	100	→ **XI**

^[a]^ For polycrystalline powders, a 1:1 ratio of integral intensities between the individual lines of a specific doublet of Lorentzian lines is expected, while deviations from this ratio are suggesting the presence of texture effects in this sample (e.g., due to compacting the powder in the sample container) and/or the presence of two nonequivalent (but not fully resolved) ^57^Fe sites with slightly different values for δ and/or $\Delta E_{Q}$.

^[b]^ Ref. [44]

^[c]^ Ref. [1]

^[d]^ Ref. [45]

^[e]^ Cp’’ = 1,3‐di‐*tert*‐butylcyclopentadienyl.

^[f]^ Fixed in the fit.

^[g]^ Fit with a fixed integral intensity ratio of 1:1 for both sub‐spectra.

^[h]^ Ref. [46]

Specifically, the spectrum of **1** displays a (slightly broadened) doublet of Lorentzian lines with an isomer shift of δ = 0.36 mm s^−1^ and a quadrupole splitting of ∆E_Q_ = 1.93 mm s^−1^ (Table 3). Notably, other compounds occasionally detected in the corresponding NMR spectra (*vide supra*) were absent in the sample used for Mössbauer spectroscopy. For comparison, the μ‐κ(*N*)‐NCO‐bridged iron(II) compound **II** exhibits a significantly higher isomer shift of δ = 1.05 mm s^−1^, whereas the quadrupole splitting of ∆E_Q_ = 1.49 mm s^−1^ is appreciably smaller (Table 3).^[^ ^1^ ^]^ Given that the isomer shift δ tends to decrease with increasing oxidation state,^[^ ^47^ ^]^ comparison with the nitrido complex **III** – which features two iron atoms in the oxidation state of + IV^[^ ^1^ ^]^ – raises intriguing questions regarding the iron oxidation states in complexes **1** to **4**. However, from the isomer shifts observed for complexes **1** to **4** no conclusive assignment can be drawn, since these values would be consistent with, e.g., with Fe(I) (S = 1/2), Fe(II) (S = 0) as well as Fe(IV) (S = 1),^[^ ^47^ ^]^ which might point to strong covalency in these compounds.

The main component in the spectrum of the arsenic complex **3**, which accounts for approximately 76% of the total spectral intensity, exhibits Mössbauer parameters (δ = 0.37 mm s^−1^ and ∆E_Q_ = 1.86 mm s^−1^) that are very similar with the related phosphorus complex **1**. In addition, two minor species are detected, each contributing roughly 12% of the total spectral intensity, for which definitive assignments could not be made. Interestingly, both of these unassigned doublets also appear in the Mössbauer spectrum of the CO‐elimination product **4** (vide infra).

Although numerous arsenic‐containing iron cyclopentadienyl species are well‐documented and well‐characterized by other techniques,^[^ ^48^, ^49^, ^50^ ^] [^ ^17^, ^51^ ^] [^ ^52^, ^53^ ^]^ to the best of our knowledge, ^57^Fe Mössbauer parameters have so far only been reported for two arsenic‐containing iron cyclopentadienyl complexes: [Cp*Fe(η^5^‐As_5_)] (δ = 0.56 mm s^−1^ and ∆E_Q_ = 0.63 mm s^−1^ recorded at T = 90 K)^[^ ^54^ ^]^ and [(Cp'Fe)_2_(μ‐As)_4_] (δ = 0.50 mm s^−1^ and ∆E_Q_ = 3.66 mm s^−1^ recorded at T = 77 K).^[^ ^55^ ^]^

The Mössbauer spectra of the CO elimination products **2** (Figure S32) and **4** (Figure S33) reveal the presence of multiple species. In the case of the phosphorus derivative, the main product **2** accounts for approximately 57% of the total spectral intensity with δ = 0.19 mm s^−1^ and ∆E_Q_ = 1.15 mm s^−1^. The remainder of the spectrum comprises contributions from the starting material **1** (22%) and ferrocene [Cp’_2_Fe] (**XII**) with ca. 8% of the total spectral intensity, as identified by their characteristic isomer shifts and quadrupole splitting values (*cf*., Table 3). Notably, only the starting material **1** was also observed by ^1^H NMR spectroscopy, while [Cp’_2_Fe] (**XII**) was not detected–likely to its minute concentration and overlapping signals. The remaining 16% of the total spectral intensity are attributed to [(Cp'Fe)_2_(μ‐η^4^:η^4^‐P_4_)] (**VI**),^[^ ^30^ ^]^ for which Mössbauer reference data is not available, but it was also detected in the ^31^P{^1^H} NMR spectrum, albeit to a lesser extent.

For the arsenic derivative, the Mössbauer spectrum of the irradiation product **4** shows three components (Table 3), which were also present in the spectrum of **3**. One minor component (7%) is again attributed to ferrocene **XII**. The two intense “main” signals, each accounting for 46% of the total spectral intensity, show Mössbauer parameters (δ = 0.44 and 0.36 mm s^−1^ and ∆E_Q_ = 1,63 and 0,87 mm s^−1^, respectively) that are close to those also observed in **3** (in smaller amounts), suggesting that one of these signals belongs to the CO‐free species **4**, while the remaining signal indicates the presence of an unidentified by‐product. Alternatively, the presence of the two nonequivalent ^57^Fe sites together with an intensity ratio close to 1:1 may also suggest that the two individual Fe sites in compound **4** are chemically not equivalent, e.g., caused by variations of the individual binding situation acting on each iron site. However, this scenario could not be confirmed based on the crystallographic data (*vide supra*). Hence, in the absence of a pure sample of **4,** a definitive assignment of these components remains speculative.

To further evaluate the effect of CO on the product mixture **4**, this mixture was exposed to CO gas, and the Mössbauer spectrum was recorded at T = 100 K (cf., Figure S34 in the Supporting Information) on the crude reaction mixture. The major product (46%) can be assigned to the iron carbonyl **XI** (Figure S35 in the Supporting Information) and a minor component of the spectrum (8%) can be assigned to ferrocene **XII** formation. Interestingly, complex **3** reformed with 8% of the total spectral intensity, and the two doublets also observed in **4** diminished to 20% of the total spectral intensity each (Table 3).

Nevertheless, further studies and more sophisticated synthetic procedures are necessary to confirm these assignments and to explore the reactivity and potential functionalization of these complexes.

### 2.5 Computational Studies

**Figure 3:**
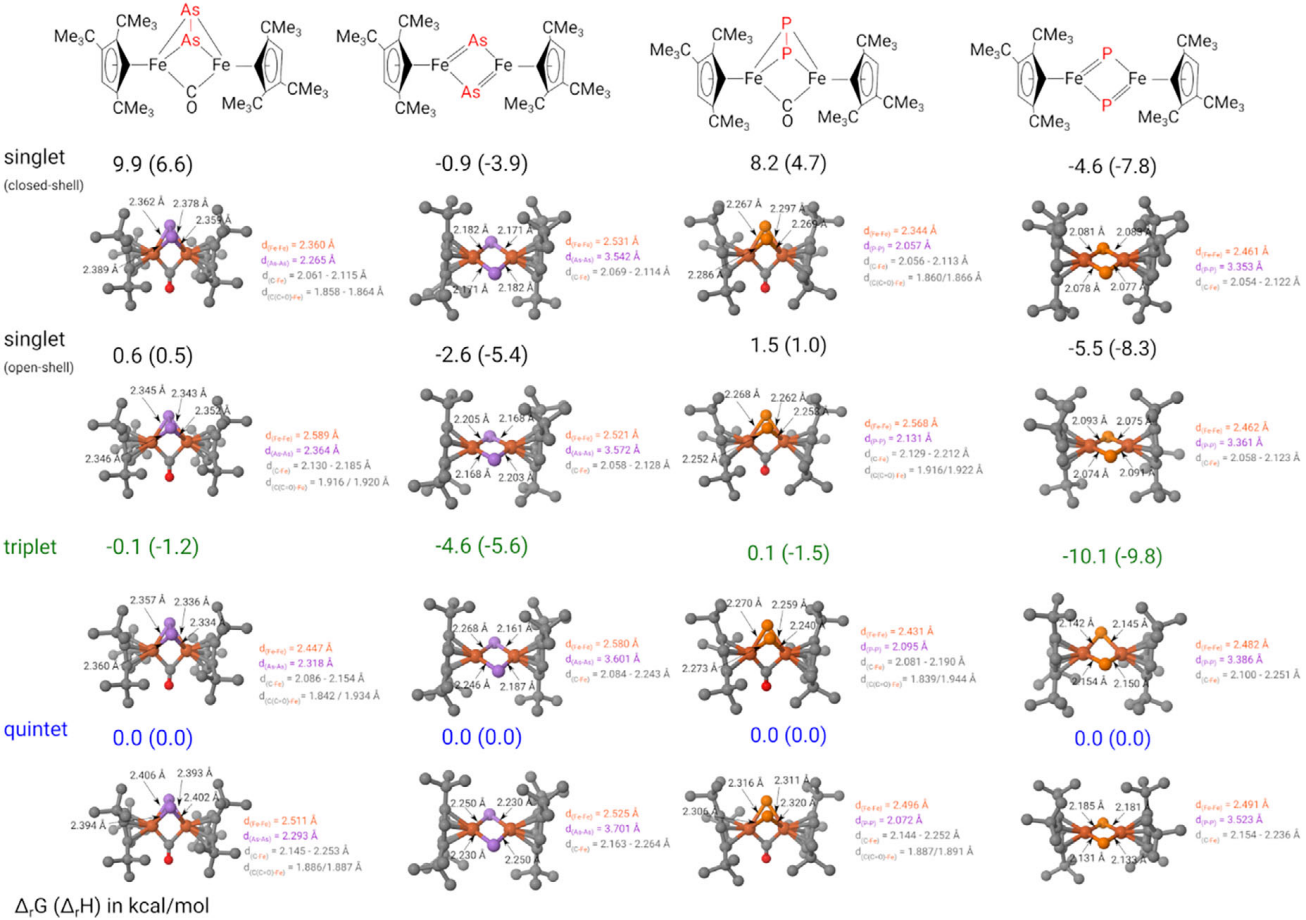
Computed structures and relative energies at the B3PW91 level of theory, including dispersion corrections.

Density functional theory (DFT) computations at the B3PW91 level of theory, including dispersion corrections, were undertaken to probe the electronic structure of complexes **1** to **4**. Structural parameters of and relative energies for various spin states (*M_S_* = 1, 3, 5, 7, and 9) were evaluated (see Supporting Information for details), while Figure 3 presents the relative energies of the lowest‐lying spin states together with their associated structural parameters. In all cases, the closed‐shell singlet state is not the ground state; instead, the triplet state is favored across all complexes **1** to **4**. However, several low‐lying excited states can also be identified computationally. For example, in complex **1**, the quintet and open‐shell singlet states lie within 1.7 kcal mol^−1^ of the triplet ground state, while for complex **3**, both the open‐shell and closed‐shell singlet states are very close in energy. Similarly, for complex **2**, a quintet and an open‐shell singlet state are energetically accessible, and for complex **4**, the closed‐shell and open‐shell singlet states are virtually degenerate. This close energetic spacing allows for the facile population of multiple spin states, which might give rise to the very unusual chemical shifts observed in these complexes.

Comparing the different optimized geometries of these low‐lying spin states with the experimental structures (see Table S2) leads to interesting insights. For complex **1**, the geometry optimized for the different spin states align relatively well with the experimentally observed structure exhibiting maximum deviations of only 0.07 Å for most parameters except for the Fe‒Fe distances. For this distance the best agreement with the experimental value is found for the triplet state. In contrast, the open‐shell singlet and quintet structures exhibit Fe‒Fe distances that are too long by 0.17 Å and 0.10 Å, respectively, likely due to enhanced electronic repulsion from two unpaired electrons on each iron center. However, including dispersion into these computations also affects this distance most, which suggests that deformations along this coordinate require minimal energy and might therefore also be influenced by packing effects in the solid state. This also implies that the Fe···Fe interaction is weak, which is inconsistent with strong Fe‐Fe bonding interactions. In complex **2**, the optimized geometries of the three low‐lying spin states (triplet, closed‐shell singlet, and open‐shell singlet) all match the experimental structure closely (see Table S2). The presence of an energetically accessible excited state may also account for the unusual NMR chemical shifts observed in these complexes. For complexes **3** and **4**, the situation mirrors that of their phosphorus homologs **1** and **2** (see Table S3). In complex **3**, the triplet geometry is lowest in energy and the bond distances agree well with the experiment, but the open‐shell and quintet states are close in energy with similar bond distances except for Fe···Fe distances. It is noteworthy that for complex **4**, the closed‐shell singlet geometry displays an As‒As distance shortened by approximately 0.08 Å relative to the experimentally determined value.

Consistent with the Dewar‐Chatt‐Duncanson model^[^ ^56^ ^]^ σ‐donation from the P_2_ π‐orbitals as well as the π‐backdonation from the two iron atoms into the π*‐systems the P‐P bond in **1** is substantially weakened as shown by the experimental P‐P bond distance (vide supra) and the Wiberg bond index (WBI) of 1.06–1.39 depending on the spin‐state assumed (Figure S37). Its arsenic analog **3** shows a similar trend in the WBI ranging from 0.95–1.30. However, in both cases the values of WBI are significantly lower than the value of 2, which was assumed based on the experimental P‐P and As‐As bond distances of 2.064(2) Å and 2.288(1) Å for **1** and **3**, respectively.

**Scheme 7:**
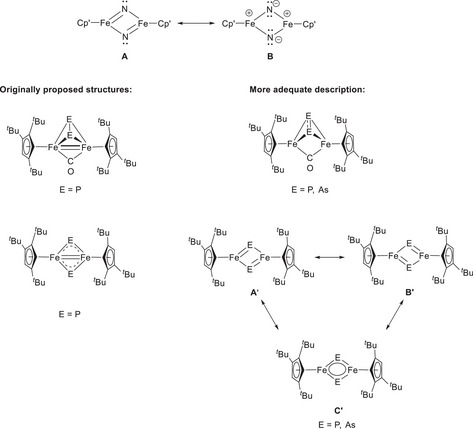
Bonding description within complexes **III** and **1** – **4**.

As pointed above the computed Fe‒Fe distances are very sensitive to dispersion suggesting, that the interaction between the two iron atoms must be rather weak casting doubts on the presence of Fe‐Fe multiple bonds as originally proposed based on the 18‐valence‐electron rule (Scheme 7). Therefore, the simple picture of relating bond distances to the presence of Fe‐Fe bond distances in these molecules should be abandoned.

Indeed, the Natural Bonding Orbital (NBO) analyses on the lowest‐energy (triplet) structures reveal that the computed Fe–Fe Wiberg Bond Indexes (WBI) for all complexes fall within the 0.3–0.4 range (Figure S37), indicating some degree of bonding interaction with relatively low covalency. This also extends to the nitrido‐bridged derivative [{Cp'Fe(μ‐N)}_2_] (**III**) which is included in Table S4, for comparison. As we have previously established for **III**, the bonding within this molecule is best represented by the two resonance structures **A** and **B**, both featuring no Fe‐Fe bond, while resonance structure **B** rationalizes the strong polarization within the Fe‐N bonds and the strong nucleophilicity of the N atoms (Scheme 7).^[^ ^1^ ^]^ For complexes **1** and **3**, no distinct Fe‒Fe bond is detected at the primary level within the NBO analysis; however, second‐order donor–acceptor interactions reveal electron donation from the Fe–E bonds or the Fe‒(CO) bonds to the other Fe center, suggesting delocalization across three centers (Fe‐E‐Fe or Fe‐(CO)‐Fe). This is further corroborated by Quantum Theory of Atoms in Molecules (QTAIM) analysis, which locates ring critical points (RCP) only in the Fe‐E‐Fe and Fe‐(CO)‐Fe fragments, with no Fe‒Fe bond critical points (BCP) identifiable (Figures S39‐S40 and S42‐S43). Such a delocalization is consistent with the observed WBI values. The situation for complexes **2** and **4** is somewhat different. While no direct Fe‒Fe bond is observed at the NBO level, second‐order interactions reveal donor‐acceptor contributions from one iron center to the other in addition to the delocalization over the Fe‐E‐Fe‐E framework (Figures S37). The Fe‐E (E = P, As) are significantly more covalent compared to their N congener (**III**). The WBI values indicate full delocalization within the Fe‐P‐Fe‐P fragment for **2** consistent with resonance structure **C’**, whereas for the As derivative **4** the degree of delocalization is significantly reduced resulting in the resonance structure **A’** or **B’**, which is not resolved in the crystal structure because of the crystallographically imposed inversion center. This additional interaction may explain why the Fe–Fe WBIs for complexes **2** and **4** are slightly higher than those for **1** and **3**. Nevertheless, QTAIM analysis again only indicates the presence of a RCP within the Fe‐E‐Fe‐E system, suggesting that any Fe‒Fe interaction is primarily attributable to electron delocalization rather than a localized bond (Figures S41 and S44).

**Scheme 8:**
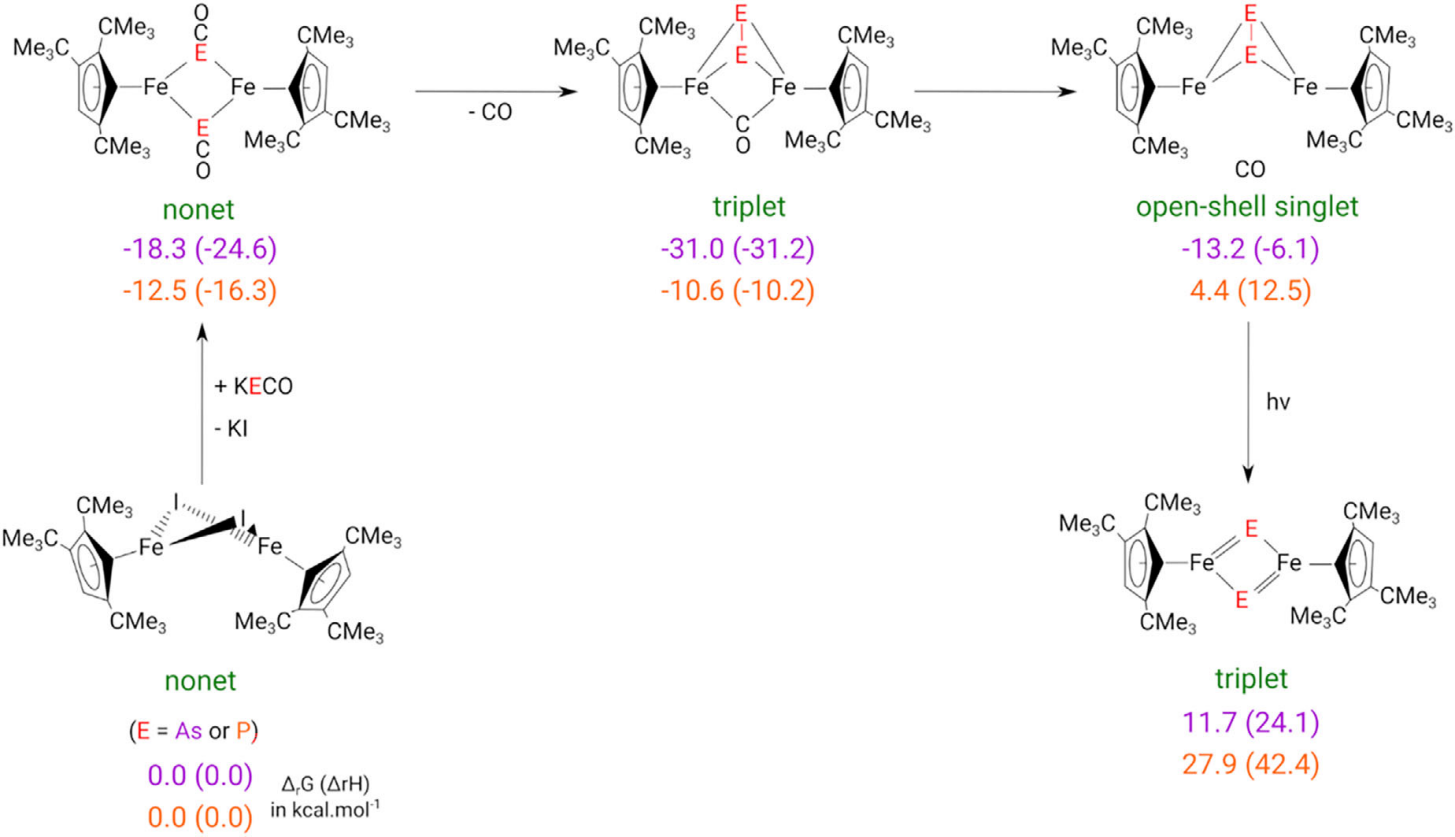
Computed reaction profile (in kcal mol^−1^) for the conversion from **I** and KOCE (E = P, As) to form complexes **3** and **4**, respectively.

Besides the analyses of the bonding situation, we were also interested in the reaction mechanism involved in the formation of the different complexes **1** to **4** (Scheme 8).

The salt metathesis reaction between **I** and OCE^−^ (E = P, As) to form the dimer [{Cp'Fe(μ‐OCE)}_2_], which subsequently loses one CO molecule to yield complexes **1** and **3**, is overall exergonic. However, the computed free energy changes Δ_r_G differ substantially: for E = As, Δ_r_G = −18.3 (with Δ_r_H = −24.6) kcal mol^−1^, whereas for E = P, Δ_r_G = –12.5 (with Δ_r_H = –16.3) kcal mol^−1^. In the case of [{Cp'Fe(μ‐OCE)}_2_] (E = P) the first CO elimination step is endothermic, though this is likely compensated by the increase in entropy associated with CO formation. Notably, the second CO elimination is in both cases thermodynamically disfavored, which rationalizes the experimental difficulties encountered and explains that [{Cp'Fe(μ‐E)}_2_] (E = P (**2**) and As (**4**)) react with CO. Moreover, following CO extrusion, the intermediate [{Cp'Fe}_2_(μ‐E_2_)] might also serve as a potential gateway to other reaction products preventing the formation of a single compound.

### 2.6 Cyclic Voltammetry

The electrochemical properties of the CO complexes **1** and **3** were investigated using cyclic voltammetry in CH_2_Cl_2_ solution (Supporting Information). Both complexes exhibit a quasi‐reversible oxidation process at E = −0.18 V for **1** and E = −0.53 V for **3** (measured at a scan rate of ν = 100 mV·s^−1^ versus the ferrocene/ferrocenium (Fc/Fc^+^) couple), which is likely a one‐electron oxidation (Table 4). In addition, an irreversible reduction event is observed at E = −1.66 V for **1** and E = −1.84 V for **2** (ν = 100 mV·s^−1^) attributed to a one‐electron reduction process. Owing to the limited quantities in which pure compounds **1** and **3** could be isolated, chemical oxidation or reduction experiments were not pursued. Complexes **2** and **4** were not analyzed electrochemically, as satisfactory purification was not achieved.

**Table 4:** Half‐wave potentials (V) and peak‐to‐peak separation (in parenthesis, V) of complexes [(Cp'Fe)_2_(μ‐η^2^:η^2^‐P_2_)(μ‐CO)] (**1**) and [(Cp'Fe)_2_(μ‐η^2^:η^2^‐As_2_)(μ‐CO)] (**3**) referenced against Fc/Fc^+^ (E = 0 V). Cyclic voltammograms were recorded in a 0.1 mol L^−1^ (*n* Bu_4_N)PF_6_ solution in CH_2_Cl_2_ at scan rates of ν = 50, 100 and 200 mV s^−1^ with a glassy carbon working electrode, a platinum wire counter electrode, and a silver wire reference electrode.

Compound	Process	50 mV s^−1^	100 mV s^−1^	200 mV s^−1^
**1**	Oxidation Reduction	−0.18 (0.47) −1.69 (0.47)	−0.18 (0.47) −1.66 (0.71)	−0.17 (0.61) −1.67 (0.90)
**3**	Oxidation Reduction	−0.51 (0.30) −1.85 (0.21)	−0.53 (0.36) −1.84 (0.26)	−0.56 (0.44) −1.89 (0.33)

### 2.7 UV Irradiation of [{Cp'Fe(CO)}_2_(μ‐CO)_2_] (6)

**Scheme 9:**
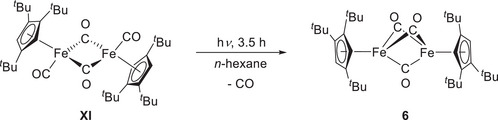
Photolysis of [{Cp'Fe(CO)}_2_(μ‐CO)_2_] (**XI**).

During our efforts to purify the CO‐bridged complex **3**, we initially hypothesized that irradiating the benzene solution would eliminate the [{Cp'Fe(CO)}_2_(μ‐CO)_2_] (**XI**) contamination by converting it into [Cp'Fe(η^5^‐C_6_H_6_)]. This species would dimerize to form the bis‐cyclohexadienyl compound [(Cp'Fe)_2_(η^5^:η^5^‐(C_6_H_6_)_2_)], which would precipitate out of the reaction mixture.^[^ ^57^ ^]^ However, instead of the anticipated dimerization, the reaction proceeded cleanly to yield the mono‐CO elimination product [(Cp'Fe)_2_(μ‐CO)_3_] (**6**) as a deeply colored, purple solid in 95% yield (Scheme 9).

The product was unequivocally identified by single‐crystal X‐ray diffraction analysis, revealing its molecular structure. A representation of the molecular structure along with selected bond parameters is provided in the Supporting Information. The molecule crystallizes in the triclinic space group P $\bar{1}$ and displays Fe‒Cp_cent_ and Fe‒Fe bond distances comparable to those of a related C_5_Me_5_‐substituted carbonyl species.^[^ ^43^ ^]^ The Fe‒Cp_cent_ distances of 1.775(1) Å and 1.778(1) Å are indicative of a low‐spin state (*S*_Fe_ = ½) for the two Fe atoms. Supporting these findings, the ^1^H NMR spectrum shows a clear transition from a diamagnetic starting material to a paramagnetic product, as evidenced by shifted and broadened resonances. Specifically, the protons of the *tert*‐butyl groups appear at δ = 9.1 and 6.9 ppm, respectively, while the CH protons are not detected. Taken together with the X‐ray diffraction data and based on literature precedents, an *S*_tot_ = 1 triplet ground state is proposed for **6** as a result of ferromagnetic coupling of two Fe(I) low‐spin centers.^[^ ^42^, ^43^, ^58^, ^59^, ^60^, ^61^ ^]^ The IR spectrum shows a single band for the CO stretching band at $\tilde{\nu }$ = 1794 cm^−1^. All analytical data align well with the those reported for the analogous C_5_R_5_^−^ substituted carbonyl species (R = H, Me, CH_2_Ph).^[^ ^42^, ^43^, ^58^, ^59^, ^60^, ^61^ ^]^

### 2.8 Epilog

In our study, we have developed a novel synthetic route toward the previously reported diphospha‐diferratetrahedrane **1** ^[^ ^5^ ^]^ and its arsenic homolog **3**, prepared from phospha‐ and arsaethynolates upon reaction with [{Cp'Fe(μ‐I)}_2_], respectively. Although the reaction conditions are relatively mild, the method led to the formation of side‐products that could not be entirely removed by chromatographic purification under an inert atmosphere, as confirmed by NMR and/or ^57^Fe Mössbauer spectroscopy. These purification challenges may also explain the limited attention these compounds have received during the last two decades. Nonetheless, our work provides additional structural and spectroscopic insights into these systems, including solid‐state zero‐field ^57^Fe Mössbauer spectra. The removal of the bridging CO ligand from compounds **1** and **3** is a demanding process, resulting in the formation of multiple reaction products. While CO extrusion from **1** proceeds relatively cleanly yielding **2** as the main product after purification, obtaining the respective As analog **4** proves even more challenging since several species are formed. Nevertheless, we succeeded in structurally authenticating **4** using X‐ray diffraction analysis. The challenges in CO extrusion are reflected in the Gibbs free energy profiles obtained from DFT computations and align with the experimentally observed (at least partially) reversible CO extrusion and the reactivity of **2** and **4** with CO to reform **1** and **3**, respectively.

During the purification of the arsenido complex **3**, we also identified the side product [{Cp'Fe(CO)}_2_(μ‐CO)_2_] (**XI**), which upon UV irradiation releases a CO molecule and forms the paramagnetic complex [(Cp'Fe)_2_(μ‐CO)_3_] (**6**) in good yield. This compound was thoroughly analyzed by ^1^H NMR spectroscopy and X‐ray diffraction analysis. In a separate experiment, thermal CO elimination from **3** intended for the synthesis of **4** resulted, however, in another side product: the Fe_3_As_6_ cluster **5**. Although obtained only in trace amounts, the few single crystals of **5** that were isolated were sufficient for a single‐crystal X‐ray diffraction analysis.

In summary, even though more than two decades have passed since *Scherer* ‘s original report on the P‐containing complexes **1** and **2**, their syntheses remain challenging surpassed only by their As analogs. To fully explore their intrinsic reactivity, further refinement in purification techniques or the development of improved synthetic routes is required. Ongoing studies are addressing these challenges and will be reported in due course.

## 3 Experimental Section

### 3.1 General Considerations

All syntheses were performed under an inert N_2_ atmosphere using a Unilab glovebox (MBraun) or standard Schlenk techniques. Solvents were dried over Na/benzophenone, distilled, and degassed prior to use. [{Cp'Fe(μ‐I)}_2_] (**I**),^[^ ^62^ ^]^ Na(OCP)(1,4‐dioxane)_x_,^[^ ^12^ ^]^ and K(OCAs)^[^ ^63^ ^]^ were prepared according to established literature procedures. All commercially available chemicals were used as received unless otherwise stated. NMR spectra were recorded on Bruker AVII300 (300 MHz), Bruker AVIII400 (400 MHz) and Bruker AVIIIHD500 (500 MHz) spectrometers at ambient temperature. For ^1^H NMR spectra, residual solvent proton signals served as internal standards. Chemical shifts (δ) are reported in parts per million (ppm); and full widths at half maximum (ν_1/2_) were given in Hertz (Hz). Elemental analyses were recorded using the Vario‐Micro‐Cube‐System. Melting points were determined visually with the MPM‐HV2 melting point meter and are uncorrected. UV/vis spectra were measured with a Varian Cary 50 Scan spectrometer, absorption features were categorized as strong (s), weak (w), shoulder (sh) and broad (br). Cyclic voltammetry was conducted with a PARSTAT‐2273 potentiostat in a 0.1 mol L^−1^ (*n*‐Bu_4_N)PF_6_ solution in CH_2_Cl_2_ at scan rates of ν = 50, 100, and 200 mV s^−1^, employing a glassy carbon working electrode, a platinum wire counter electrode, and a silver wire reference electrode. All cyclic voltammograms were referenced against Fc/Fc^+^ (E = 0 V). For X‐ray diffraction analysis, crystals were mounted on glass fibers or hair in inert oil. Data were collected on Oxford diffraction systems using mirror‐focused Cu‐K_α_ or monochromatic Mo‐K_α_ radiation. Structures were refined anisotropically against F^2^ using SHELXL‐97^[^ ^64^, ^65^ ^]^ and the OLEX2 software,^[^ ^66^ ^]^ with hydrogen atoms incorporated either as rigid methyl groups or using the riding model.

### 3.2 Syntheses

$\left[{\left({\mathrm{Cp}}^{\prime }\mathrm{Fe}\right)}_{2}\left(\mu -\eta^{2}:\eta^{2}-P_{2}\right)\left(\mu -\mathrm{CO}\right)\right]$ (**1**). *Method A*. In a pressurizable 25 mL‐glass reactor, [Na(OCP)(1,4‐dioxane)_3.6_] (142 mg, 0.360 mmol, 2.0 equiv.) was suspended in benzene (3 ml). A solution of [{Cp'Fe(μ‐I)}_2_] (**I**; 148 mg, 0.180 mmol, 1.0 equiv.) in benzene (5 ml) was then added under stirring. The reactor was sealed and charged with CO gas to an overall pressure of approximately 8.5 bar. After stirring the mixture at room temperature for 3 hours, the CO atmosphere was replaced with N_2_, and the resultant dark green liquid was transferred into a Schlenk flask. The solvent was removed under reduced pressure, and the residue was extracted with *n*‐hexane (10 ml). Following filtration over celite, the filtrate was dried in vacuum, and preparative thin‐layer chromatography (silica, sample dissolved in Et_2_O, *n*‐hexane as eluent) yielded [(Cp'Fe)_2_(μ‐η^2^:η^2^‐P_2_)(μ‐CO)] (**1**) as a dark green, crystalline solid. Yield: 35 mg (0.052 mmol, 29%).

*Method B*. A mixture of [{Cp'Fe(μ‐I)}_2_] (**I**; 200 mg, 0.240 mmol, 1.0 equiv.) and [Na(OCP)(1,4‐dioxane)_1.2_] (91 mg, 0.485 mmol, 2.0 equiv.) was combined, and THF (15 ml) was added. The reaction mixture was stirred at ambient temperature for 16 hours, after which the solvent was removed under reduced pressure to yield a green residue. This residue was extracted with *n*‐hexane (15 ml) and filtered over celite. Evaporation of the filtrate under reduced pressure afforded a crude product, which was purified by column chromatography on silica using *n*‐hexane or *n*‐pentane as the eluent to yield the product as a dark green, microcrystalline powder. Yield: 40 mg (0.0598 mmol, 25%). R_f_: 0.08 (on silica in *n*‐hexane or *n*‐pentane; on the column, the fraction is extremely broadened). Melting point: 242 °C. Decomposition point: ca. 280 °C. Anal. calc. (%) for C_35_H_58_OP_2_Fe_2_ (668.49 g/mol) C 62.89, H 8.75; found C 62.46, H 8.41. UV/Vis (THF, 22 °C): λ = 308 (s), 390 (sh), 602 (br) nm. ^1^H NMR (500 MHz, C_6_D_6_): δ = 6.02 (s, 4 H, 4 × Cp’‐*H*), 1.27 (s, 36 H, 4 × C(C*H*_3_)_3_), 1.10 (s, 18 H, 2 × C(C*H*_3_)_3_) ppm. ^13^C{^1^H} NMR (126 MHz, C_6_D_6_): δ = 126.0 (C_q_, 2 × *C*C(CH_3_)_3_), 113.3 (br., C_q_, 4 × *C*C(CH_3_)_3_), 81.6 (CH, 4 × Cp’‐*C*H), 38.4 (CH_3_, 2 × C(*C*H_3_)_3_), 36.6 (CH_3_, 4 × C(*C*H_3_) _3_), 32.2 (C_q_, 4 × *C*(CH_3_) _3_), 29.1 (C_q_, 2 × *C*(CH_3_)_3_) ppm. The ^13^C{^1^H} resonance for the carbonyl group (CO) was not observed. ^31^P{^1^H} NMR (203 MHz, C_6_D_6_): δ = 780.3 (br. s) ppm. HRMS (ESI, MeCN/toluene 1:1): m/z = 668.26557 (calc. 668.26574).

$\left[{\left({\mathrm{Cp}}^{\prime }\mathrm{Fe}\right)}_{2}\left(\mu -\eta^{2}:\eta^{2}-P_{2}\right)\right]$ (**2**). This reaction must be conducted on small scale. Consequently, the total amount of starting material [(Cp'Fe)_2_(μ‐η^2^:η^2^‐P_2_)(μ‐CO)] (**1**; 122 mg, 0.183 mmol, 1.0 equiv.) was divided in twelve separate batches. In a 25 mL‐quartz tube, [(Cp'Fe)_2_(μ‐η^2^:η^2^‐ P_2_)(μ‐CO)] (**1**; approximately 9–15 mg per batch) was dissolved in *n*‐hexane (10 ml). The tube was then slightly evacuated until the solvent began to boil at room temperature. The reaction mixture was irradiated at 253 nm for 5 hours at ca. 35 °C, during which the solution gradually changed color from dark green to red‐brown. After removing the solvent under reduced pressure, a brown residue was obtained. The combined residues from all batches were recrystallized from *n*‐hexane at ‐30 °C, affording product **2** as a red‐brown, microcrystalline solid. However, ^1^H NMR spectroscopy indicated that the product was still contaminated by approximately 16% of the starting material. Yield: 80 mg (0.125 mmol, 68%). Melting point: 164 °C. Anal. Calc. (%) for C_34_H_58_P_2_Fe (640.48 g/mol) C 63.76, H 9.13; found C 63.20, H 8.97. ^1^H NMR (300 MHz, C_6_D_6_): δ = 6.55 (s, 4 H, 4 × Cp’‐*H*), 1.33 (s, 36 H, 4 × C(C*H*_3_)_3_), 0.33 (s, 18 H, 2 × C(C*H*_3_)_3_) ppm. ^13^C{^1^H} NMR (76 MHz, C_6_D_6_): δ = 106.2 (C_q_, 2 × *C*C(CH_3_)_3_), 104.5 (C_q_, 4 × *C*C(CH_3_)_3_), 81.6 (CH, 4 × Cp’‐*C*H), 34.2 (CH_3_, 4 × C(*C*H_3_)_3_), 32.3 (C_q_, 4 × *C*(CH_3_)_3_), 30.7 (CH_3_, 2 × C(*C*H_3_)_3_), 27.6 (C_q_, 2 × *C*(CH_3_)_3_) ppm. ^31^P{^1^H} NMR (203 MHz, C_6_D_6_): δ = 1407.1 (s) ppm.

$\left[{\left({\mathrm{Cp}}^{\prime }\mathrm{Fe}\right)}_{2}\left(\mu -\eta^{2}:\eta^{2}-As_{2}\right)\left(\mu -\mathrm{CO}\right)\right]$ (**3**). [Cp'Fe(μ‐I)_2_] (**I**; 100 mg, 0.120 mmol, 1.0 equiv.) was dissolved in Et_2_O (8 ml), and a suspension of K(OCAs) (34 mg, 0.240 mmol, 2.0 equiv.) in Et_2_O (5 ml) was added to the solution. The mixture was stirred at room temperature for 20 hours, during which a dark green suspension formed. Following the reaction, the mixture was dried under reduced pressure and the residue was extracted with *n*‐hexane (20 ml). After filtration over celite and subsequent drying of the filtrate, the resulting dark, brownish‐green solid was purified by column chromatography on silica using *n*‐hexane as the eluent. The product eluted as a broad, dark green fraction with R_f_ values between 0.70 – 0.40, while the third fraction (R_f_ = 0.32 – 0.10) contained the side product [(Cp'Fe(CO))_2_(μ‐CO)_2_], rendering complete separation challenging. After the first purification step, 45 mg of a dark green, crystalline solid consisting predominatly of [(Cp'Fe)_2_(μ‐η^2^:η^2^‐As_2_)(μ‐CO)] (**3**; 88%) and [{Cp'Fe(CO)}_2_(μ‐CO)_2_] (**X**; 12%). A second round of column‐chromatographic purification reduced contamination, leaving the desired product with only 5% of **X**, as determined by ^1^H NMR spectroscopy. Yield: 39 mg (0.0516 mmol, 43%). Melting point: 225 °C. Decomposition point: ca. 255 °C. Anal. calc. (%) for C_35_H_58_OAs_2_Fe_2_ (756.38 g/mol) C 55.58, H 7.73; found C 55.12, H 7.69. UV/Vis (THF, 22 °C): λ = 312 (ss), 368 (sh), 602 (br) nm. ^1^H NMR (500 MHz, C_6_D_6_): δ = 10.48 (br. s, 4 H, 4 × Cp’‐*H*), 2.60 (s, 18 H, 2 × C(C*H*_3_)_3_), 1.87 (s, 36 H, 4 × C(C*H*_3_)_3_) ppm. ^13^C{^1^H} NMR (126 MHz, C_6_D_6_): δ = 267.8 (C_q_, *C*O), 81.0 (CH_3_, 2 × C(*C*H_3_)_3_), 53.6 (CH_3_, 4 × C(*C*H_3_)_3_), 45.9 (CH, 4 × Cp’‐*C*H), 32.5 (C_q_, 4 × *C*(CH_3_)_3_), 18.8 (C_q_, 2 × *C*(CH_3_)_3_) ppm. The ^13^C{^1^H} resonances for the quaternary ring carbon atoms (CC(CH_3_)_3_) were not observed. HRMS (ESI, MeCN/toluene 1:1): m/z = 756.16034 (calc. 756.16141).

$\mathrm{Attempted}\;\mathrm{Synthesis}\;\mathrm{of}[{\left({\mathrm{Cp}}^{\prime }\mathrm{Fe}\right)}_{2}\left(\mu -\eta^{2}:\eta^{2}-As_{2}\right)]$ (**4**). Analogous to the procedure for [(Cp'Fe)_2_(μ‐η^2^:η^2^‐P_2_)] (**2**), the reaction was performed on a small scale. A total amount of starting material [(Cp'Fe)_2_(μ‐η^2^:η^2^‐As_2_)(μ‐CO)] (**3**; 50 mg, 0.0661 mmol, 1 equiv.; contaminated with ca. 7% [{Cp'Fe(CO)}_2_(μ‐CO)_2_] (**X**)) was divided in five separate batches. In each reaction, 10 mg of [(Cp'Fe)_2_(μ‐η^2^:η^2^‐As_2_)(μ‐CO)] (**3**) was dissolved in benzene in a quartz tube. The tube was then slightly evacuated until the solvent began to boil at room temperature. The reaction mixture was subsequently irradiated with 253 nm light for 4 hours while maintaining a temperature of approximately 35 °C, which resulted in a color change from green to dark red. Following irradiation, the solvent was removed under reduced pressure, yielding a dark red solid. Analysis by ¹H NMR and ⁵⁷Fe Mössbauer spectroscopy revealed that the product consisted of a mixture of compounds **3**, **4**, and additional unidentified impurities. Attempts to improve the conversion or purify the desired product by recrystallization or column chromatography were unsuccessful.

$\left[{\left({\mathrm{Cp}}^{\prime }\mathrm{Fe}\right)}_{3}{\left(As_{3}\right)}_{2}\right]$ (**5**). To thermally synthesize [(Cp'Fe)_2_(μ‐η^2^:η^2^‐As_2_)] (**4**), [(Cp'Fe)_2_(μ‐η^2^:η^2^‐As_2_)(μ‐CO)] (**3**; 12 mg, 0.0159 mmol, 1 equiv.) was heated to 600 °C under an inert N_2_ atmosphere, until the solid melted. The flask was then evacuated, and the residue was extracted with *n*‐hexane. A grey solid was removed by filtration over Celite, and the filtrate was concentrated before being stored at ‐30 °C. This procedure yielded a few dark green crystals suitable for X‐ray diffraction analysis, which were identified as the Fe_3_As_6_ cluster **5**. A depiction of its solid‐state molecular structure can be found in the Supporting Information.

$\left[{\left({\mathrm{Cp}}^{\prime }\mathrm{Fe}\right)}_{2}{\left(\mu -\mathrm{CO}\right)}_{3}\right]$ (**6**). In a quartz glass Schlenk tube, [{Cp'Fe(CO)}_2_(μ‐CO)_2_] (**X**; 30 mg, 0.0430 mmol, 1.0 equiv.) was dissolved in *n*‐hexane (100 ml). The flask was then slightly evacuated until the solvent started to boil at room temperature. The reaction mixture was irradiated with 253 nm light for 3.5 hours at ca. 35 °C. The solvent was removed under reduced pressure, and the residue was recrystallized from *n*‐hexane at −30 °C to yield the product **6** as a deeply colored, purple crystalline solid. Yield: 27 mg (0.0410 mmol, 95%). Decomposition point: ca. 185 °C. Anal. calc. (%) for C_37_H_58_O_3_Fe_2_ (662.56 g/mol) C 67.07, H 8.82; found C 66.97, H 8.95. UV/Vis (*n*‐hexane, 22 °C): λ = 524 nm. ^1^H NMR (300 MHz, C_6_D_6_): δ = 9.1 (ν_1/2_ = 180 Hz, 18 H, 2 × Cp’‐*H*), 6.9 (ν_1/2_ = 220 Hz, 36 H, 4 × C(C*H*_3_)_3_) ppm. The resonance for the CH protons was not observed.

### 3.3 Zero‐field ^57^Fe Mössbauer Spectroscopy

Polycrystalline powders of compounds **1**–**4**, “**4 + CO**”, and **XI** were prepared with an area density corresponding to ca. 0.09 – 0.14 mg ^57^Fe/cm^2^ and filled in sample containers made of PEEK (polyether ether ketone) or Teflon. The measurements were performed on a commercial (*WissEl* and *Halder*) transmission spectrometer with sinusoidal velocity sweep. The velocity calibration was done with an α‐Fe foil at ambient temperature. The temperature‐dependent measurements on complex **1** were executed with a (*Janis*) closed‐cycle cryostat with helium exchange gas adjusted at a pressure of ca. 10–50 mbar during the measurement. The temperature was controlled and recorded with a calibrated Si diode, located close to the sample container, providing a temperature stability of better than ± 0.1 K. The minimum experimental line widths (FWHM) were < 0.26 mm s^−1^. The measurements on complexes **2** to “**4 + CO**” were conducted on a (*CryoVac*) continuous‐flow cryostat with comparable specifications regarding the geometry and sample environment as described above in case of the *Janis* closed‐cycle cryostat (but with slightly lower experimental line width (FWHM) of ca. < 0.22 mm s^−1^). The nominal activity of the Mössbauer source was 50 mCi of ^57^Co in a rhodium matrix, which was stored at ambient temperatures during the measurements. The isomer shifts were specified relative to metallic iron at room temperature but were not corrected in terms of the second‐order Doppler shift. The data analyses of the recorded spectra were carried out using the software package NORMOS.^[^ ^67^ ^]^

## Supporting Information

The Supporting Information contains NMR, UV/vis, CV, and Mössbauer spectra, X‐ray crystallographic^[^ ^68^ ^]^ and computational details.

## Conflict of Interest

The authors declare no conflict of interest.

## Supporting information



Supporting Information

## Data Availability

The data that support the findings of this study are available in the supplementary material of this article.
