# Supplementary material for: Reactivity of [{Cp'Fe(μ‐I)}2] toward 3‐phospha‐ and 3‐arsaethynolate
Source: Chemistry. 2025 Jul 4;31(41):e202501339. doi: 10.1002/chem.202501339 (PMC12284617; doi:10.1002/chem.202501339)
Supplement: Supplementary file 1 — Supporting Information [file CHEM-31-e202501339-s001.docx]

Reactivity of [{Cp'Fe(μ-I)}_2_] towards 3-phospha- and 3-arsaethynolate

Katharina Münster,^[a]^ Jasper Mindner,^[a]^ William-Dale Möller,^[a]^ Dirk Baabe,^[a]^ Iker del Rosal,^[b]^ Laurent Maron,^[b]^ and Marc D. Walter*^[a]^

[a] Institut für Anorganische und Analytische Chemie, Technische Universität Braunschweig, Hagenring 30, 38106 Braunschweig (Germany)

[b] Université de Toulouse et CNRS, INSA, UPS, UMR5215, LPCNO, 135 Avenue de Rangueil, 31077 Toulouse (France)

**Table of contents**

1. NMR Spectroscopy S2
2. X-ray Crystallography S12
3. UV/vis Spectroscopy S15
4. Cyclic Voltammetry S17
5. Zero-field ^57^Fe Mössbauer Spectroscopy S20
6. Computational Details S22
7. References S69
8. **NMR Spectroscopy**
   1. **[(Cp’Fe)_2_(μ-η^2^:η^2^-P_2_)(μ-CO)] (1)**


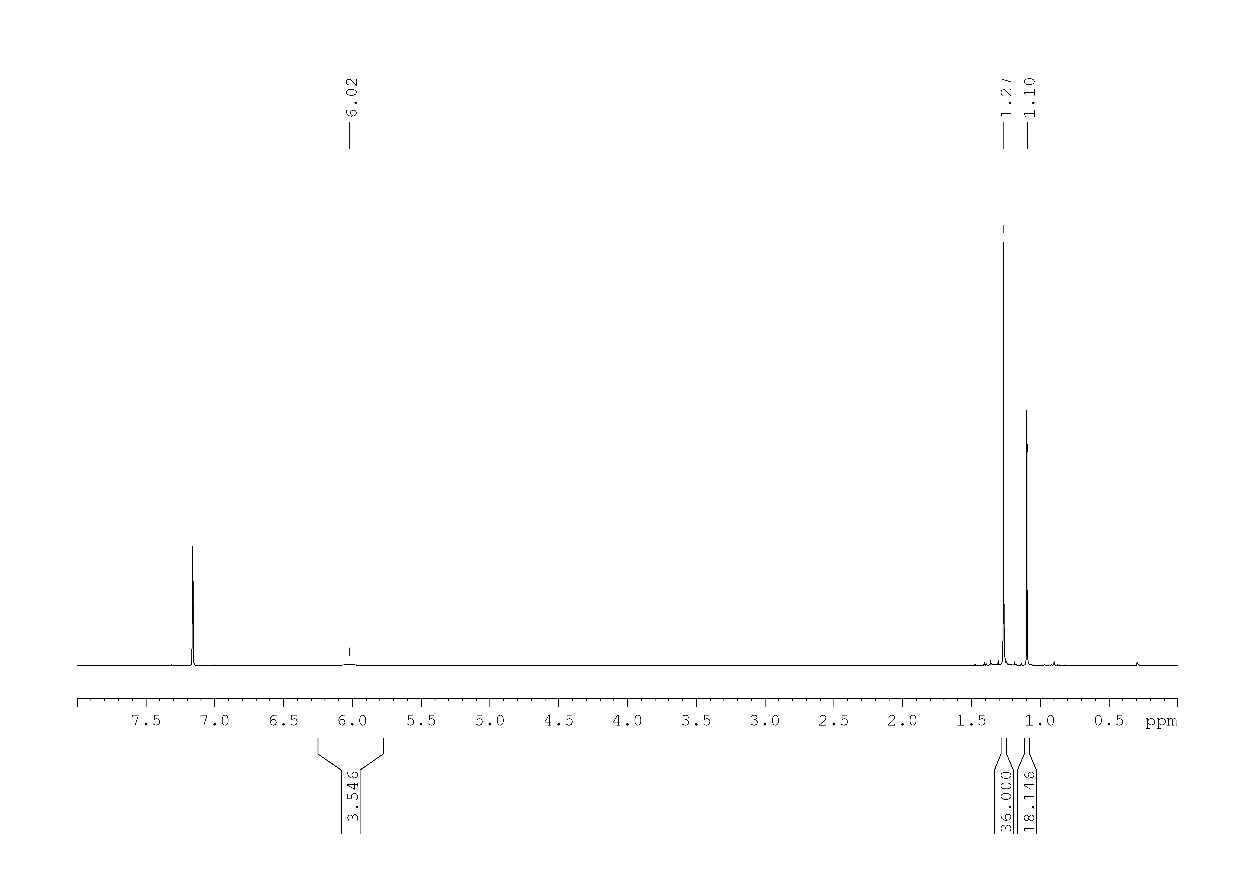


**Figure S1.** ^1^H NMR spectrum (500 MHz, C_6_D_6_) for [(Cp’Fe)_2_(μ-η^2^:η^2^-P_2_)(μ-CO)] (**1**).


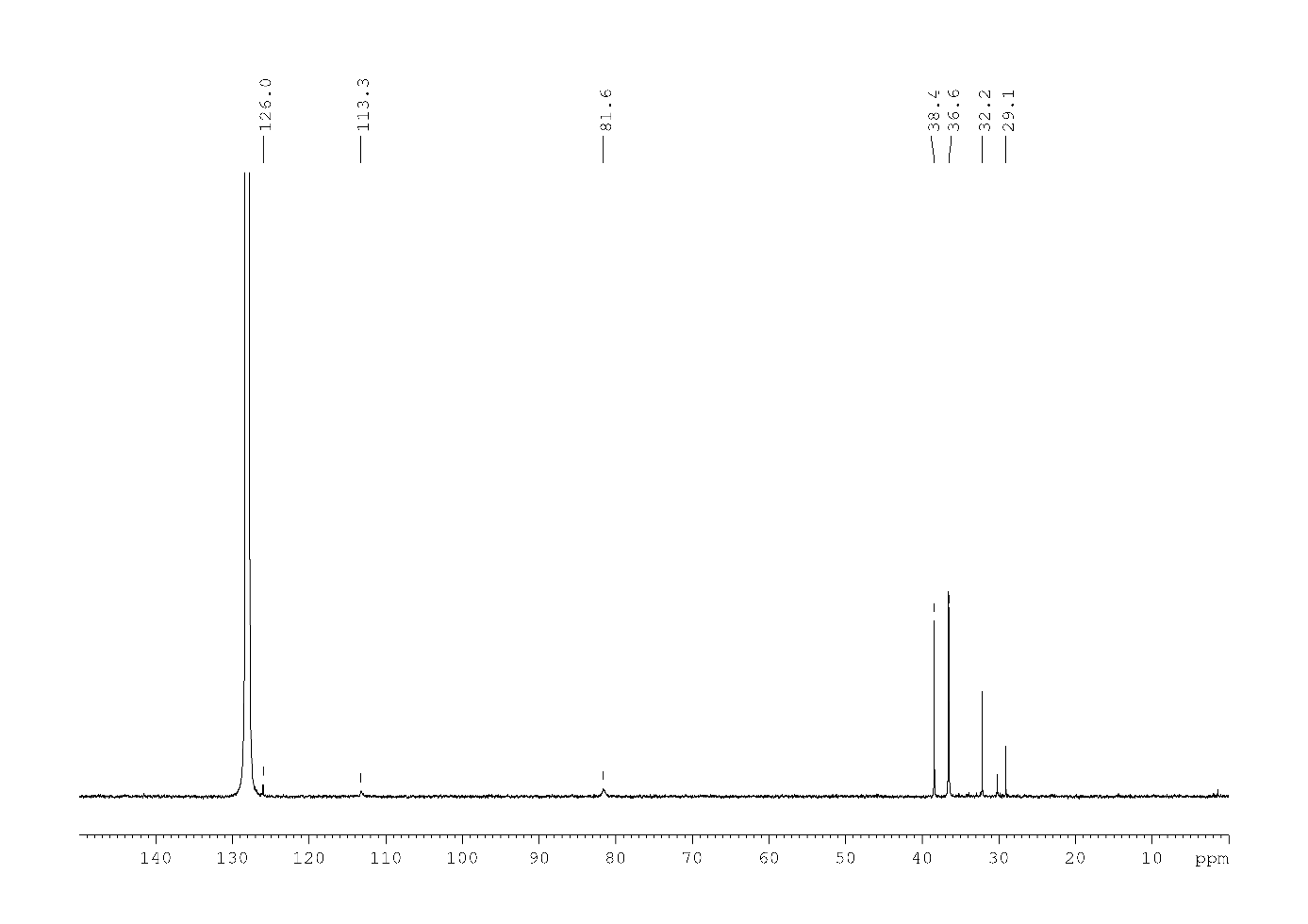


**Figure S2.** ^13^C{^1^H} NMR spectrum (126 MHz, C_6_D_6_) for [(Cp’Fe)_2_(μ-η^2^:η^2^-P_2_)(μ-CO)] (**1**).


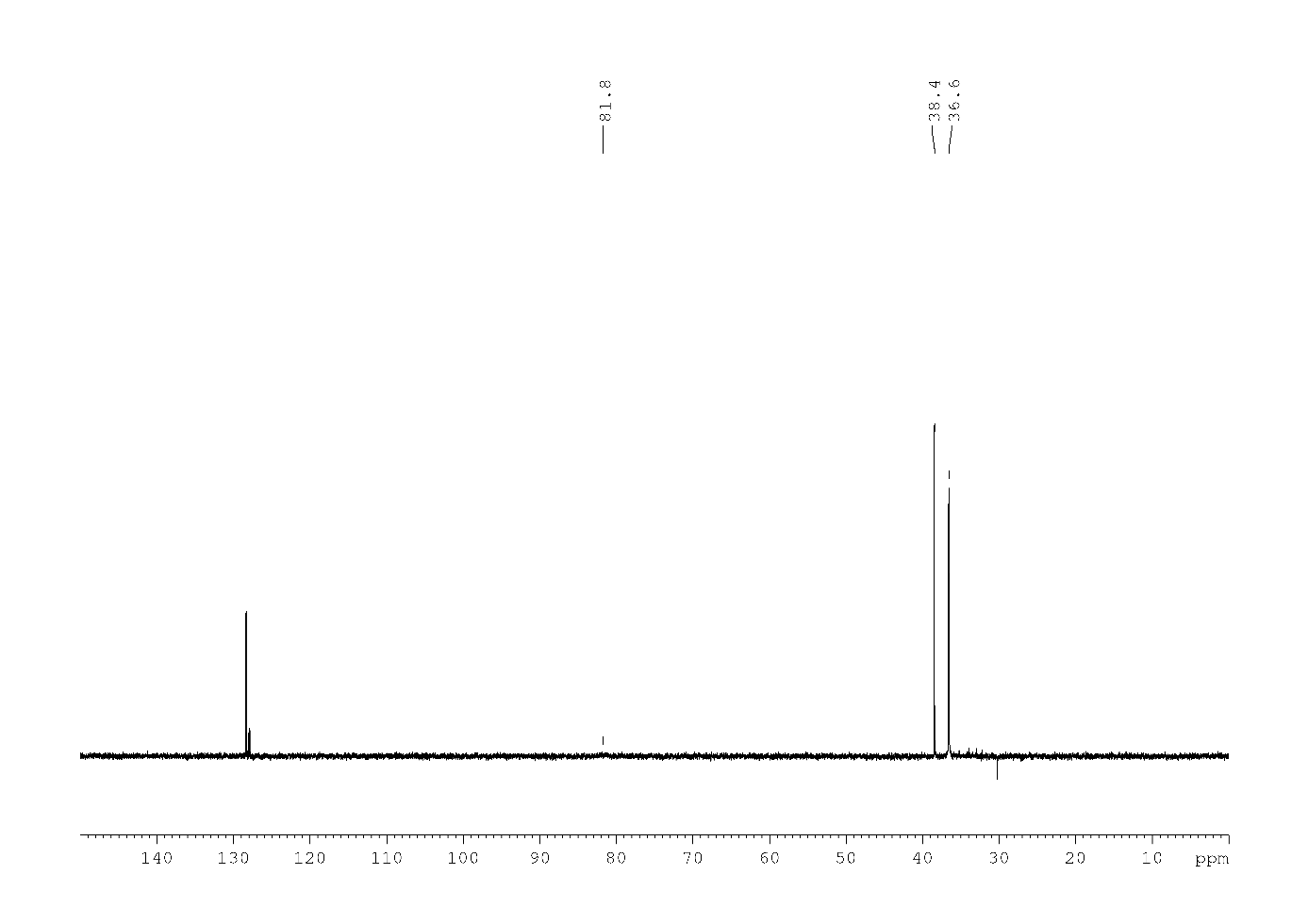


**Figure S3.** ^13^C{^1^H} dept-135 NMR spectrum (126 MHz, C_6_D_6_) for [(Cp’Fe)_2_(μ-η^2^:η^2^-P_2_)(μ-CO)] (**1**).


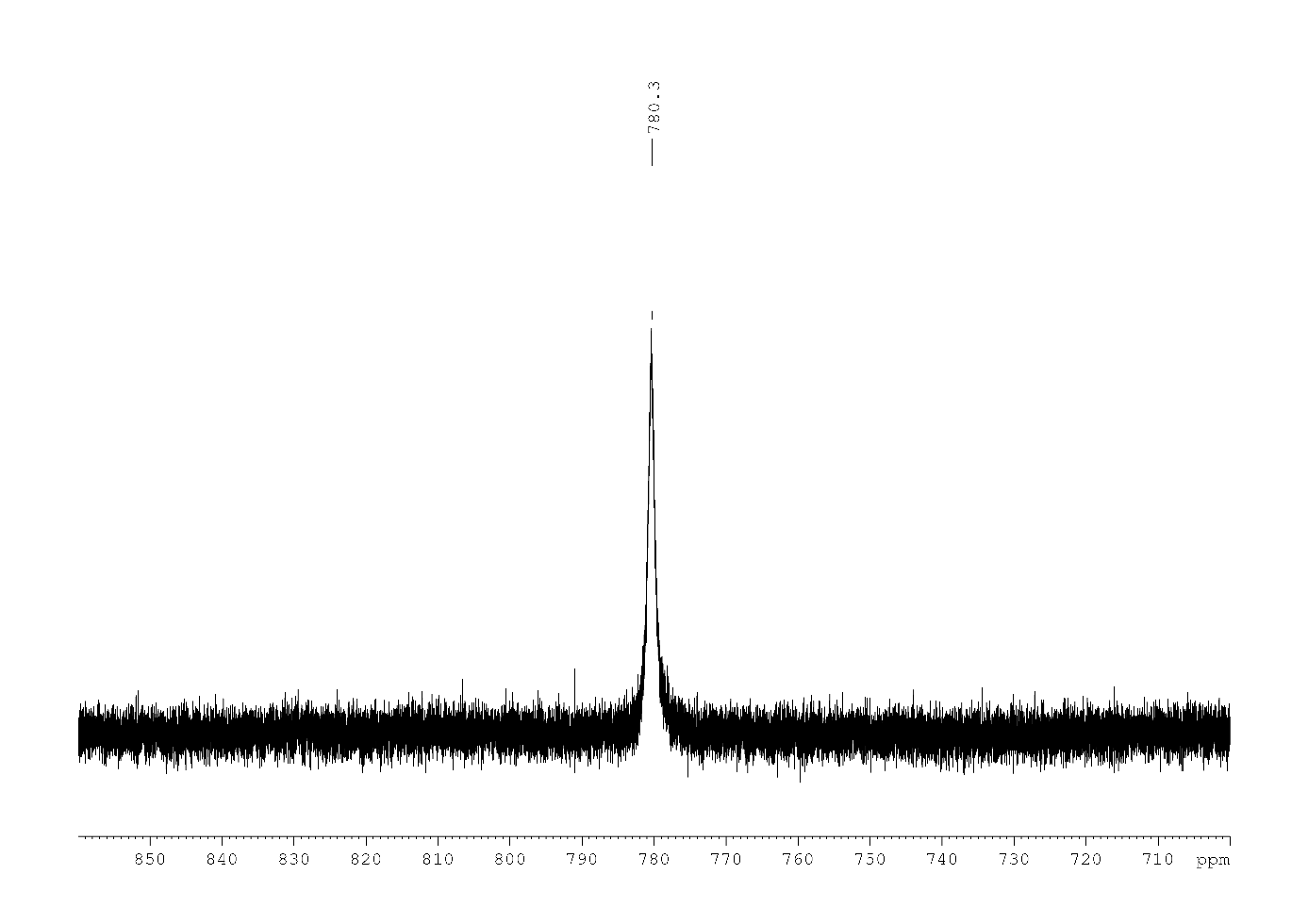


**Figure S4.** ^31^P{^1^H} NMR spectrum (203 MHz, C_6_D_6_) for [(Cp’Fe)_2_(μ-η^2^:η^2^-P_2_)(μ-CO)] (**1**).


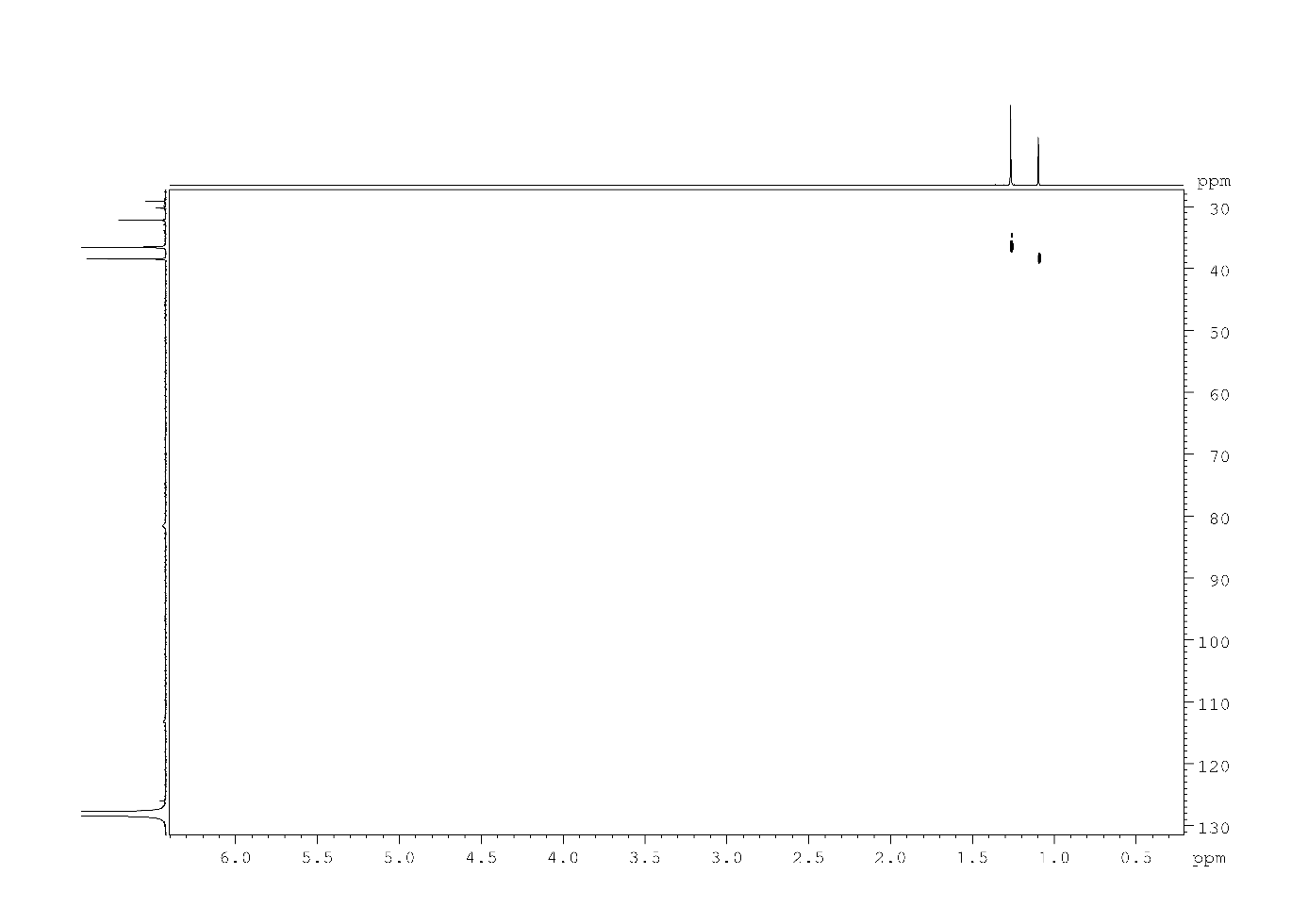


**Figure S5.** ^1^H,^13^C-HSQC NMR spectrum for [(Cp’Fe)_2_(μ-η^2^:η^2^-P_2_)(μ-CO)] (**1**).


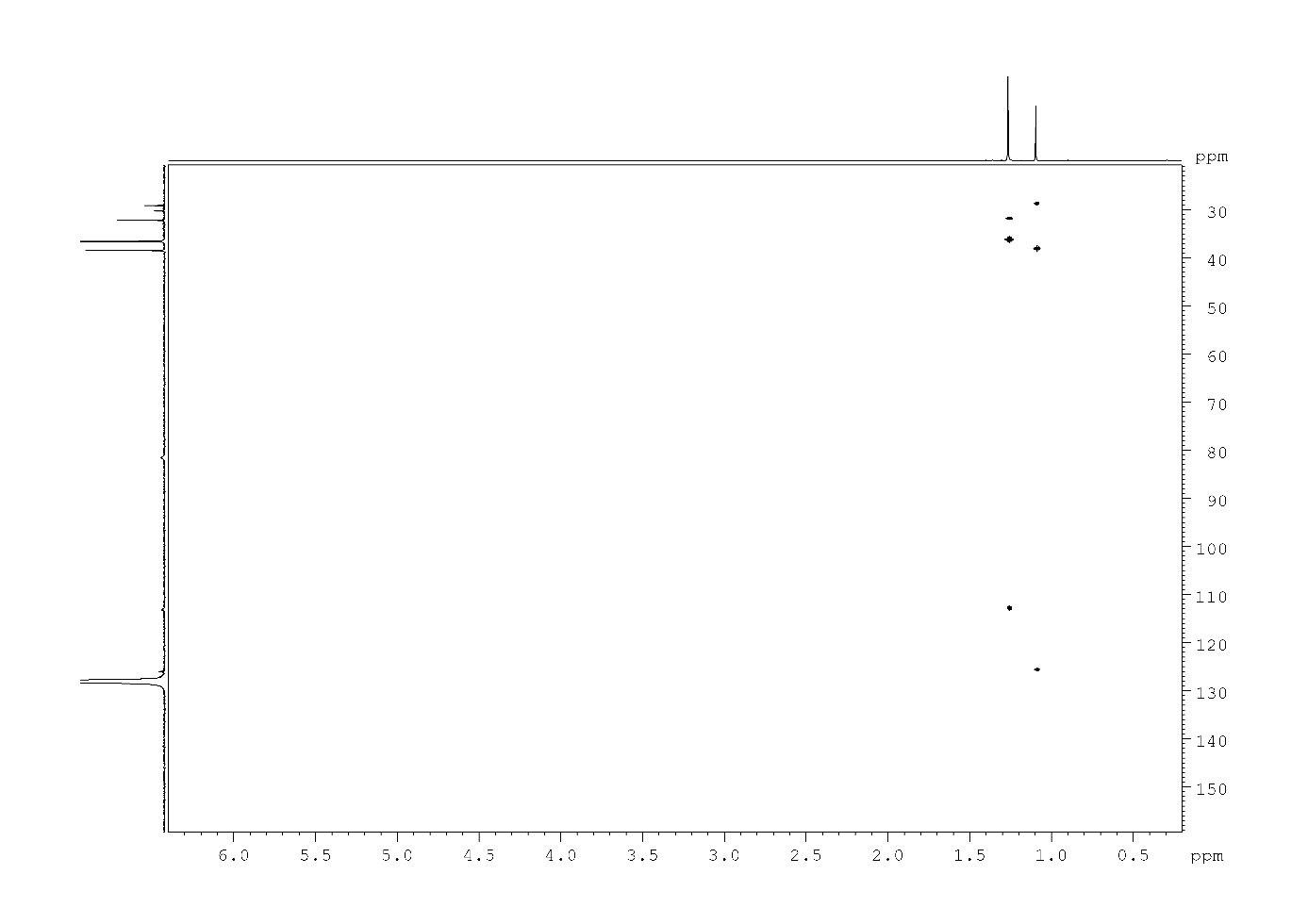


**Figure S6.** ^1^H,^13^C-HMBC NMR spectrum for [(Cp’Fe)_2_(μ-η^2^:η^2^-P_2_)(μ-CO)] (**1**).

- 1. **[(Cp’Fe)_2_(μ-η^2^:η^2^-P_2_)] (2)**

**
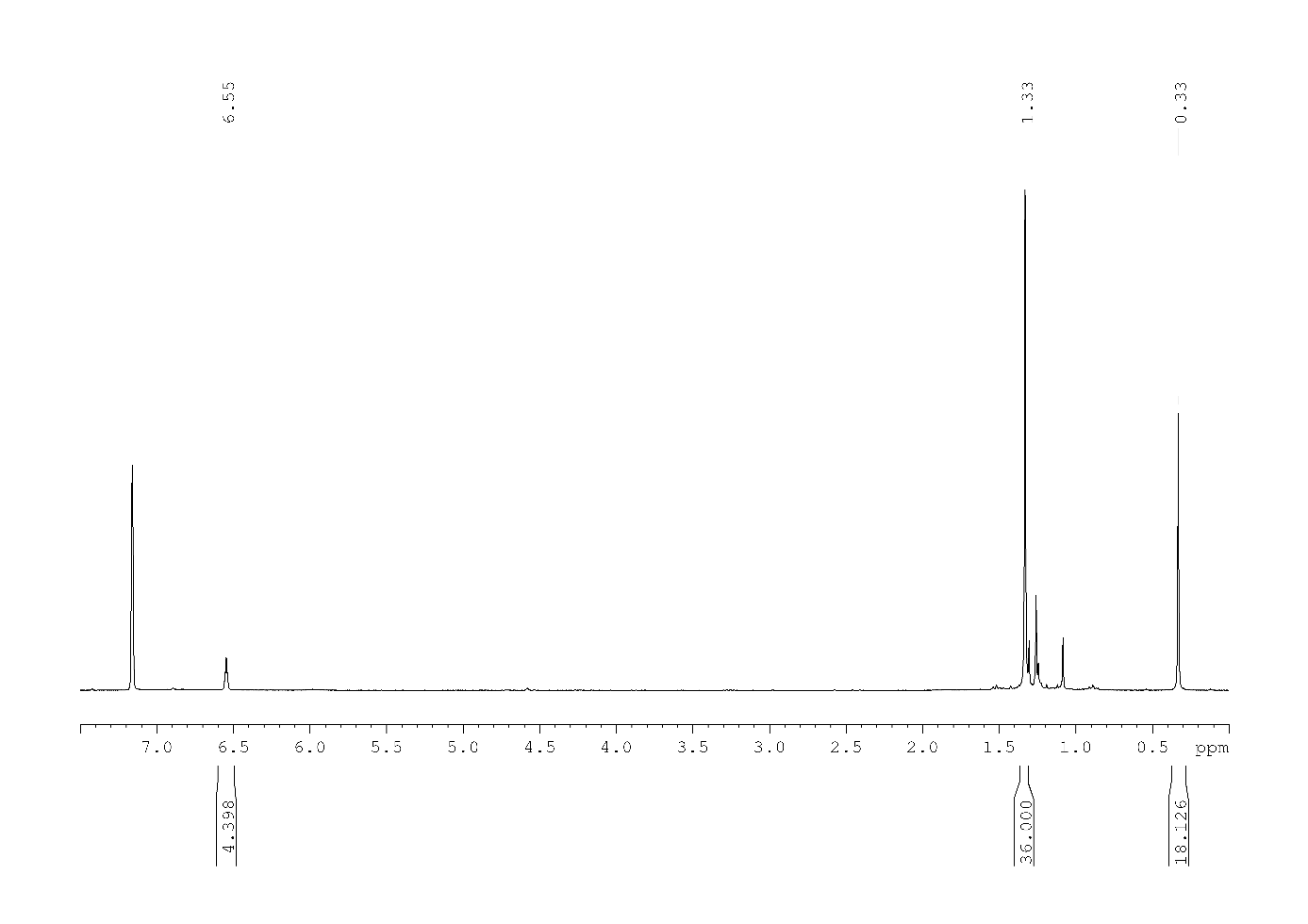
**

**Figure S7.** ^1^H NMR spectrum (300 MHz, C_6_D_6_) for [(Cp’Fe)_2_(μ-η^2^:η^2^-P_2_)] (**2**). The marked resonances correspond to complex **2**.


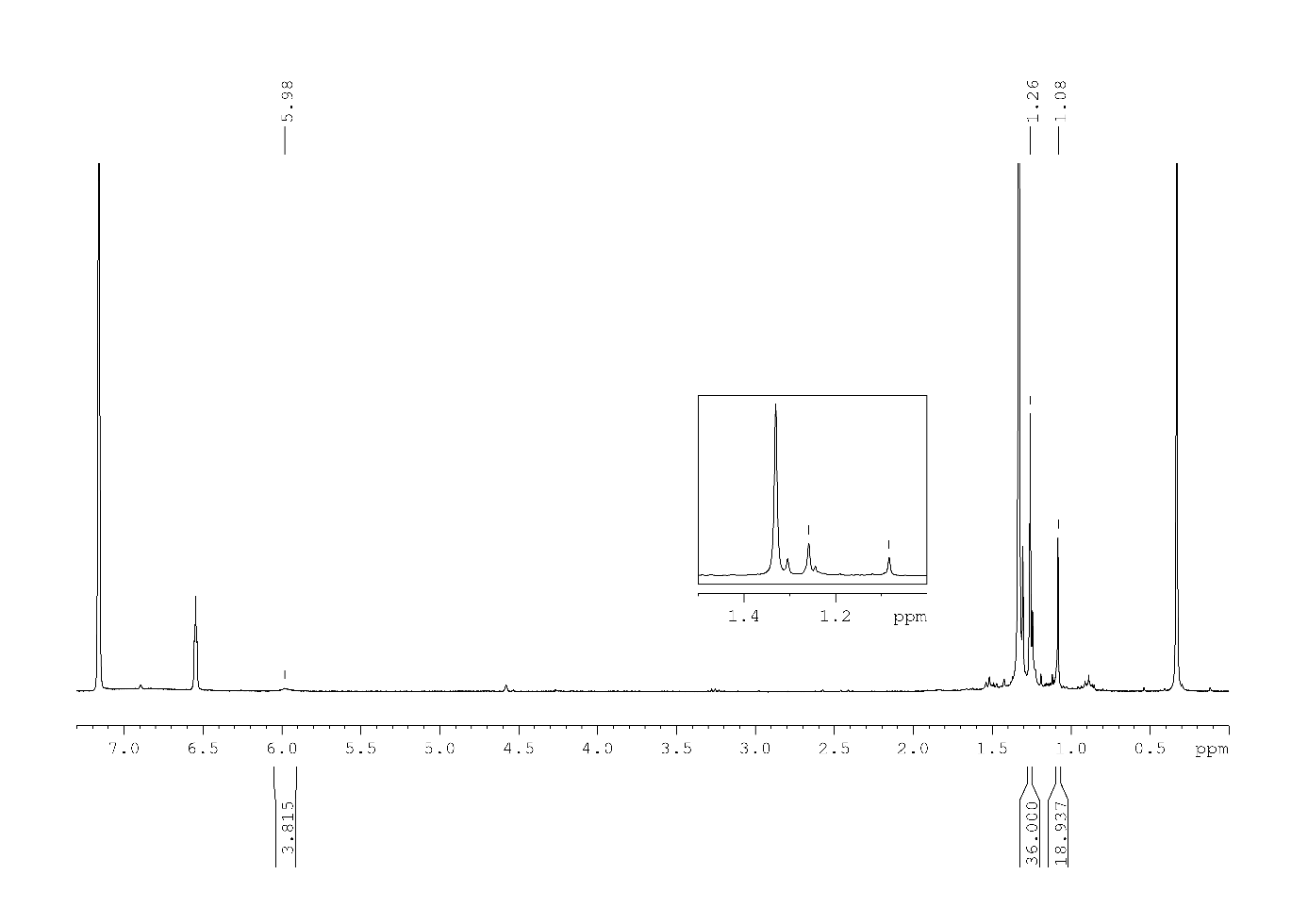


**Figure S8.** ^1^H NMR spectrum (300 MHz, C_6_D_6_) for [(Cp’Fe)_2_(μ-η^2^:η^2^-P_2_)] (**2**). The marked resonances correspond to the starting material **1**.


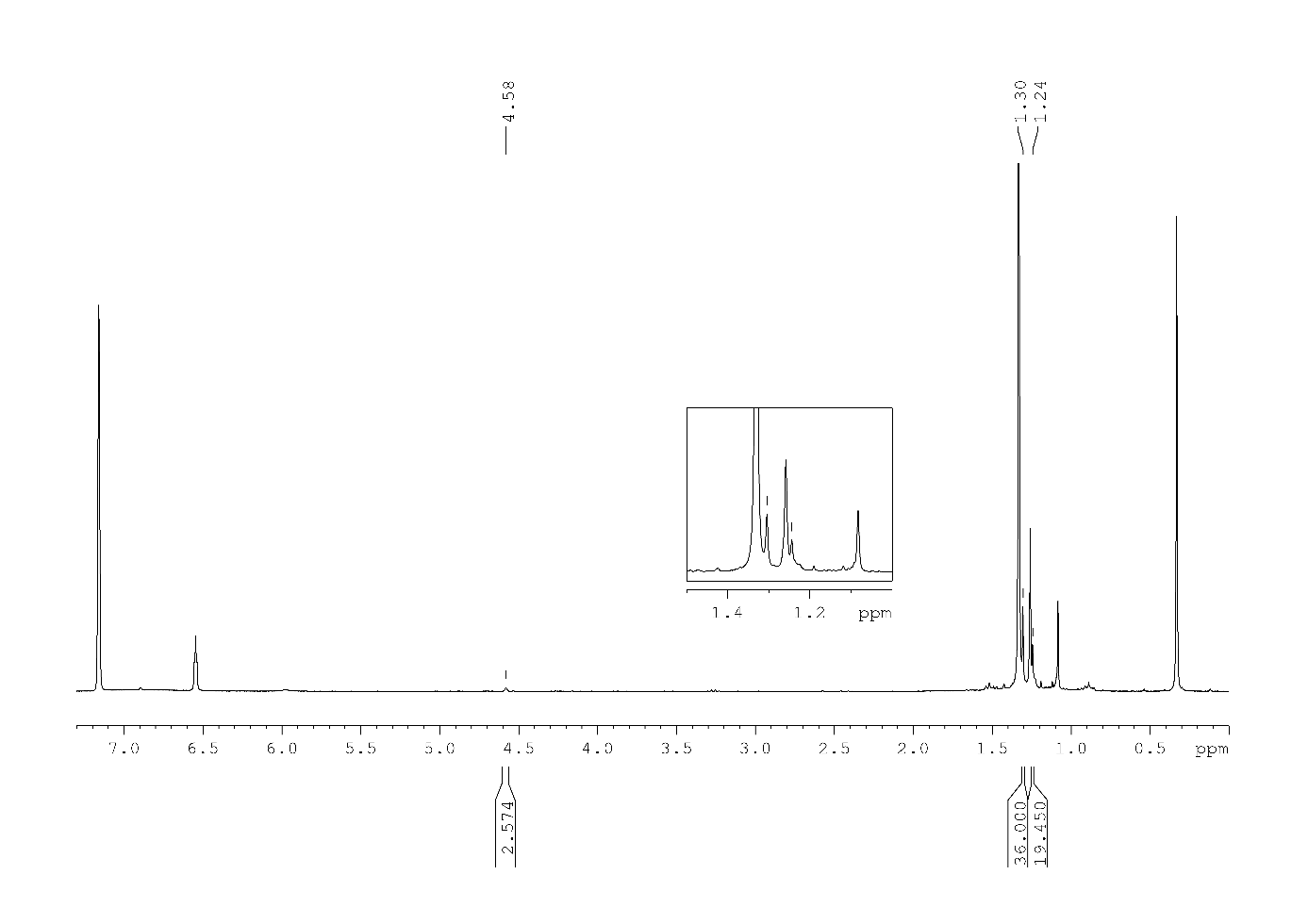


**Figure S9.** ^1^H NMR spectrum (300 MHz, C_6_D_6_) for [(Cp’Fe)_2_(μ-η^2^:η^2^-P_2_)] (**2**). The marked resonances correspond to the side-product **VI**.


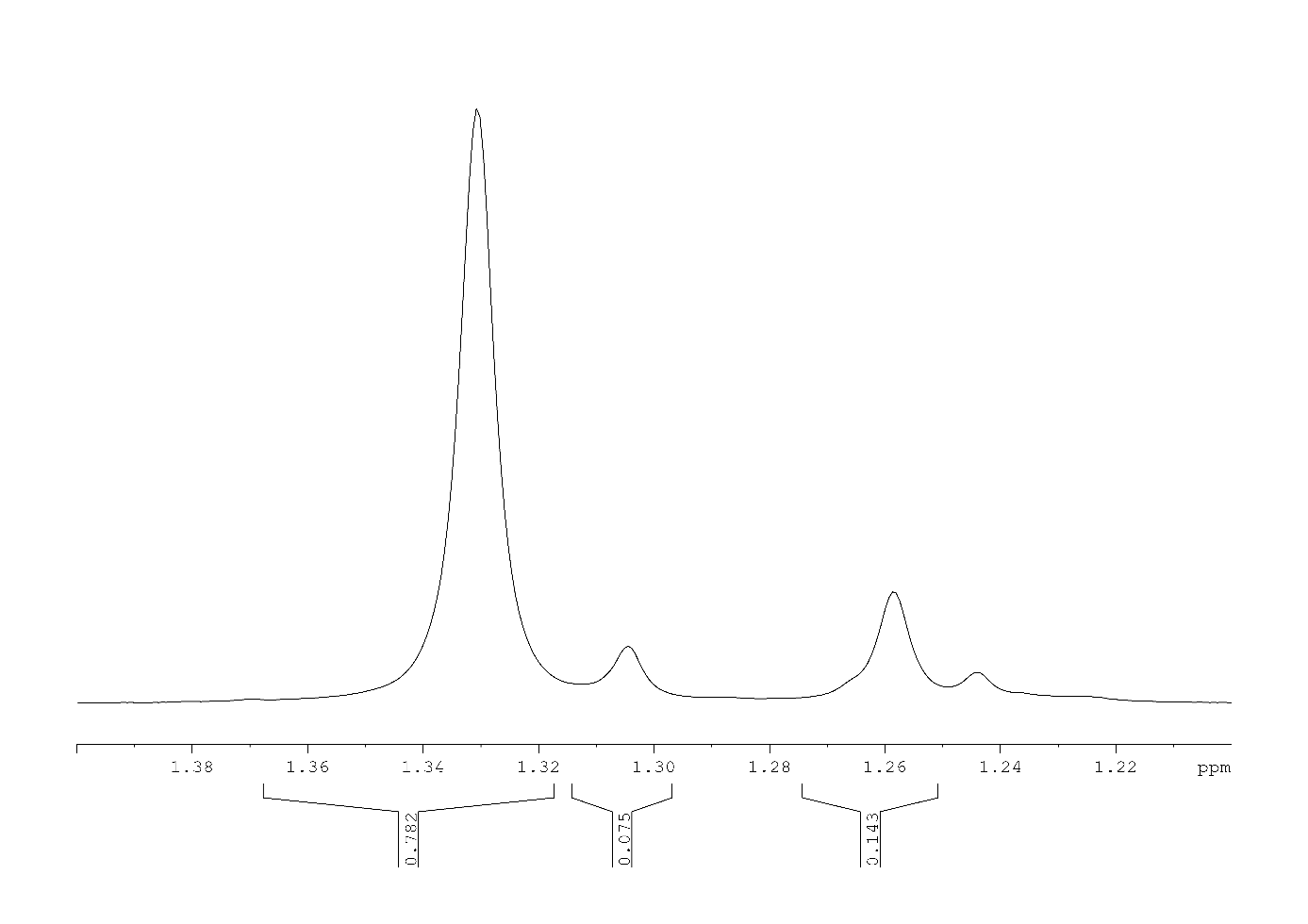


**Figure S10.** ^1^H NMR spectrum (300 MHz, C_6_D_6_) for [(Cp’Fe)_2_(μ-η^2^:η^2^-P_2_)] (**2**) and the impurities **1** and **VI** with integration of the resonances corresponding to two tert-butyl groups, respectively, to demonstrate the compound fractions.

**
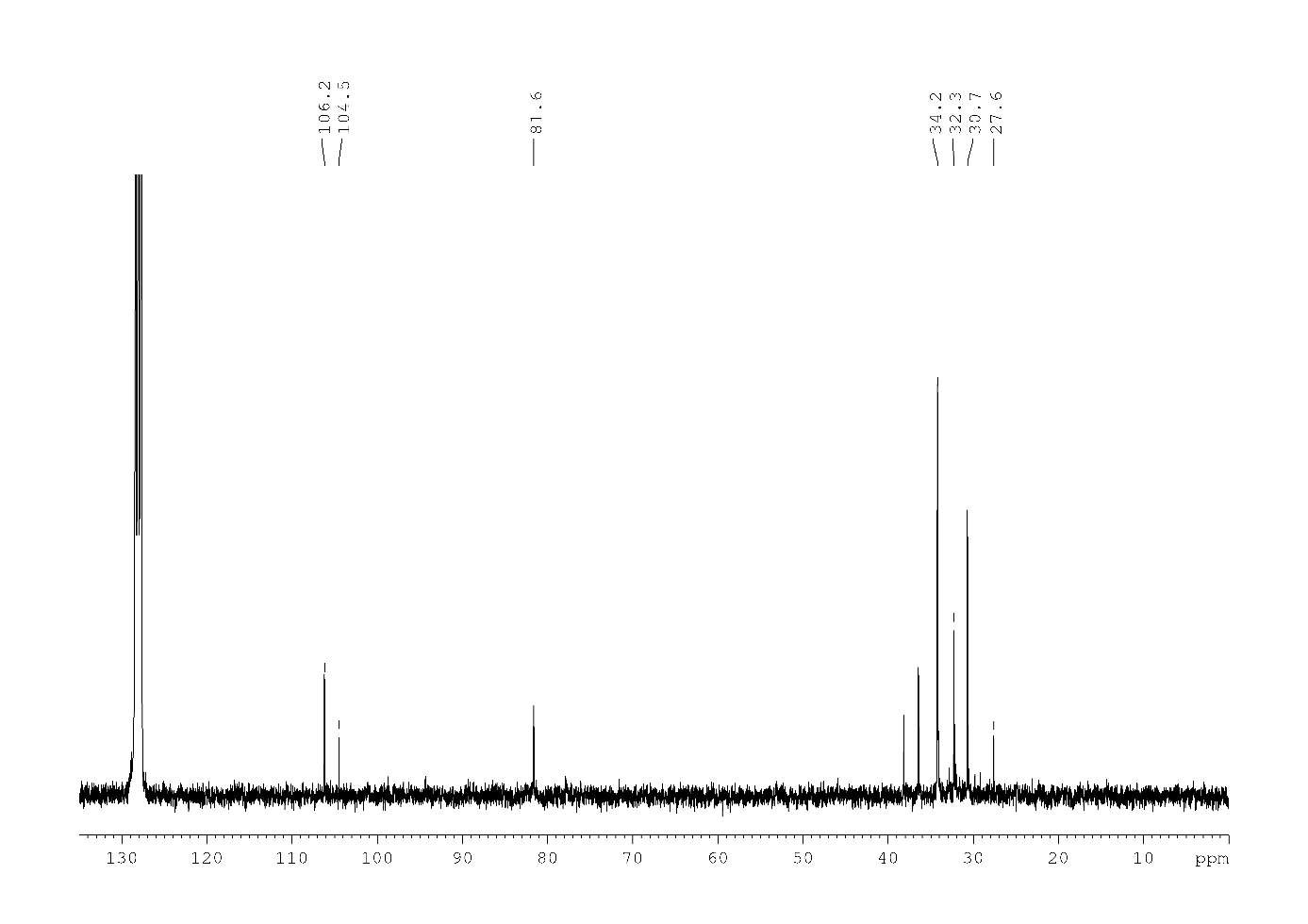
**

**Figure S11.** ^13^C{^1^H} NMR spectrum (76 MHz, C_6_D_6_) for [(Cp’Fe)_2_(μ-η^2^:η^2^-P_2_)] (**2**).


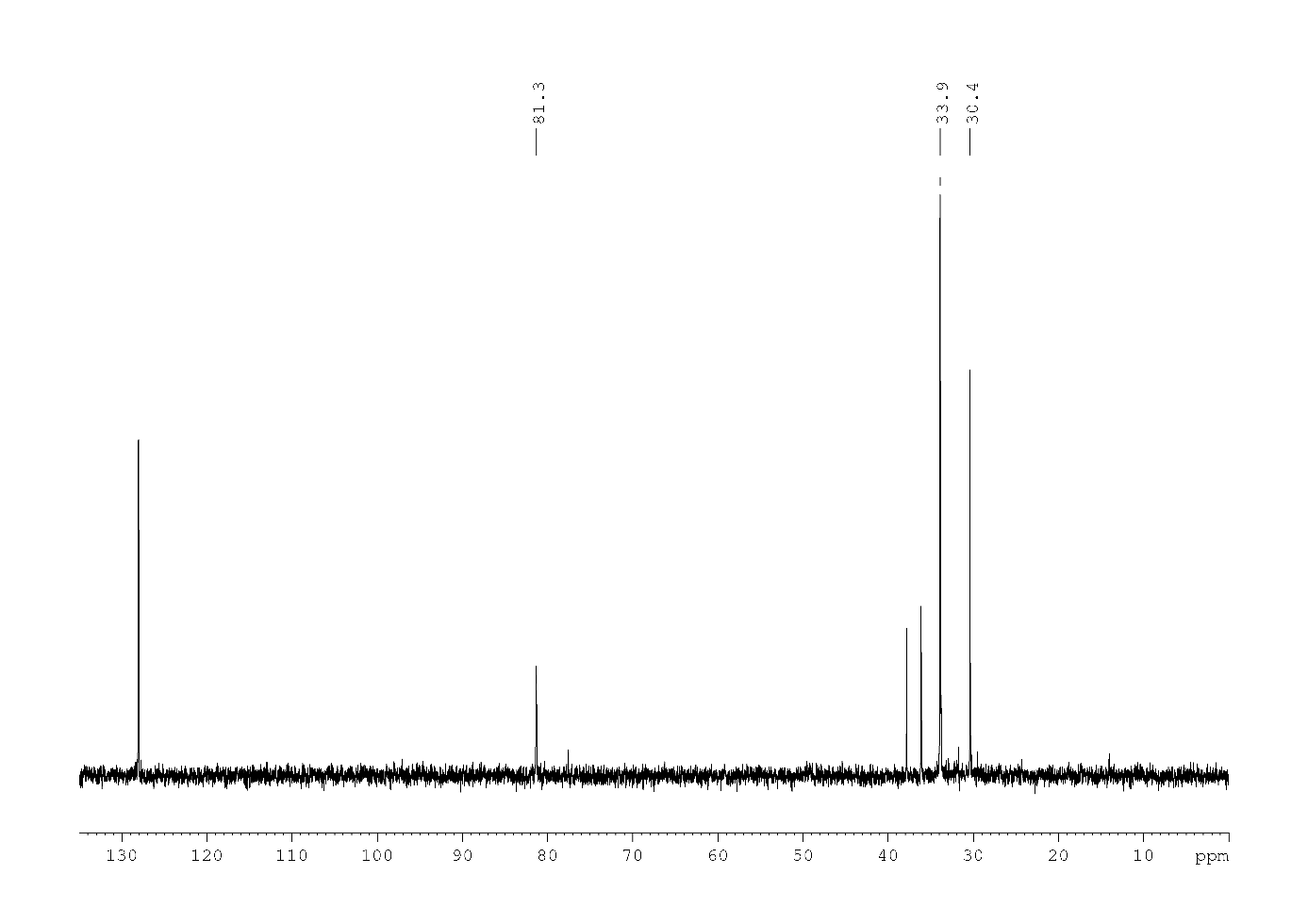


**Figure S12.** ^13^C{^1^H} dept-135 NMR spectrum (76 MHz, C_6_D_6_) for [(Cp’Fe)_2_(μ-η^2^:η^2^-P_2_)] (**2**).


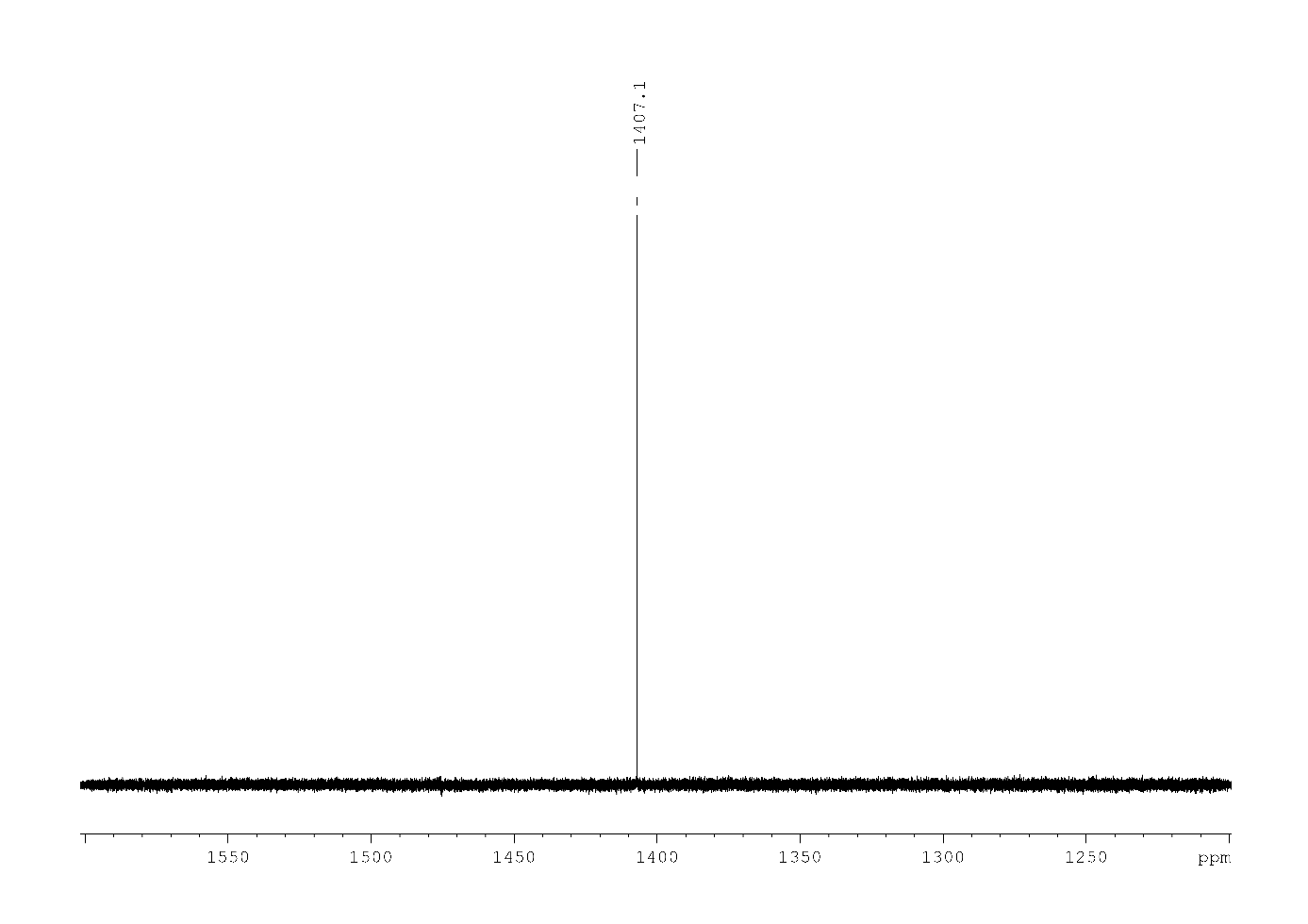


**Figure S13.** ^31^P{^1^H} NMR spectrum (203 MHz, C_6_D_6_) for [(Cp’Fe)_2_(μ-η^2^:η^2^-P_2_)] (**2**).


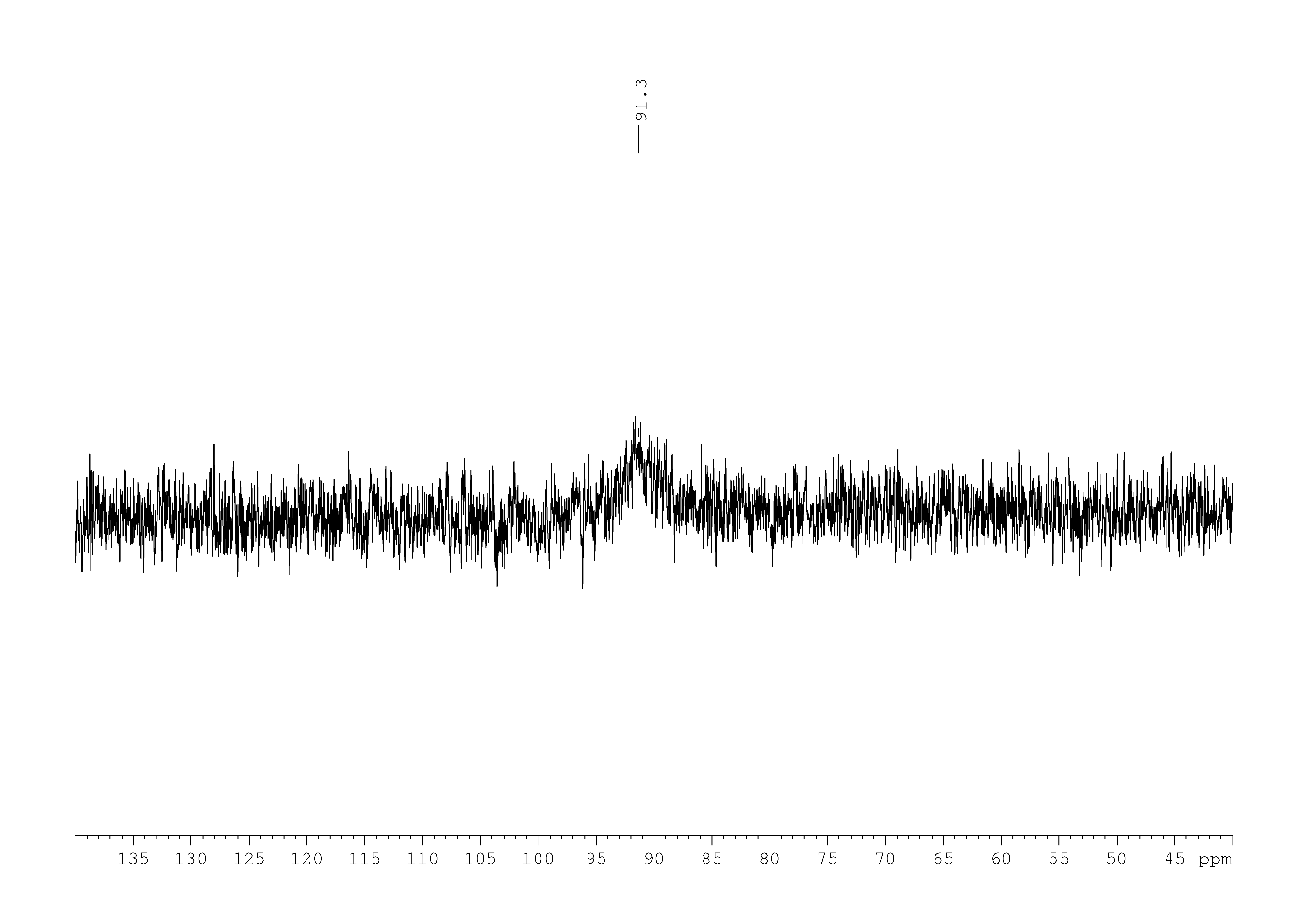


**Figure S14.** ^31^P{^1^H} NMR spectrum (203 MHz, C_6_D_6_) for the side-product **VI**.

- 1. **[(Cp’Fe)_2_(μ-η^2^:η^2^-As_2_)(μ-CO)] (3)**

**
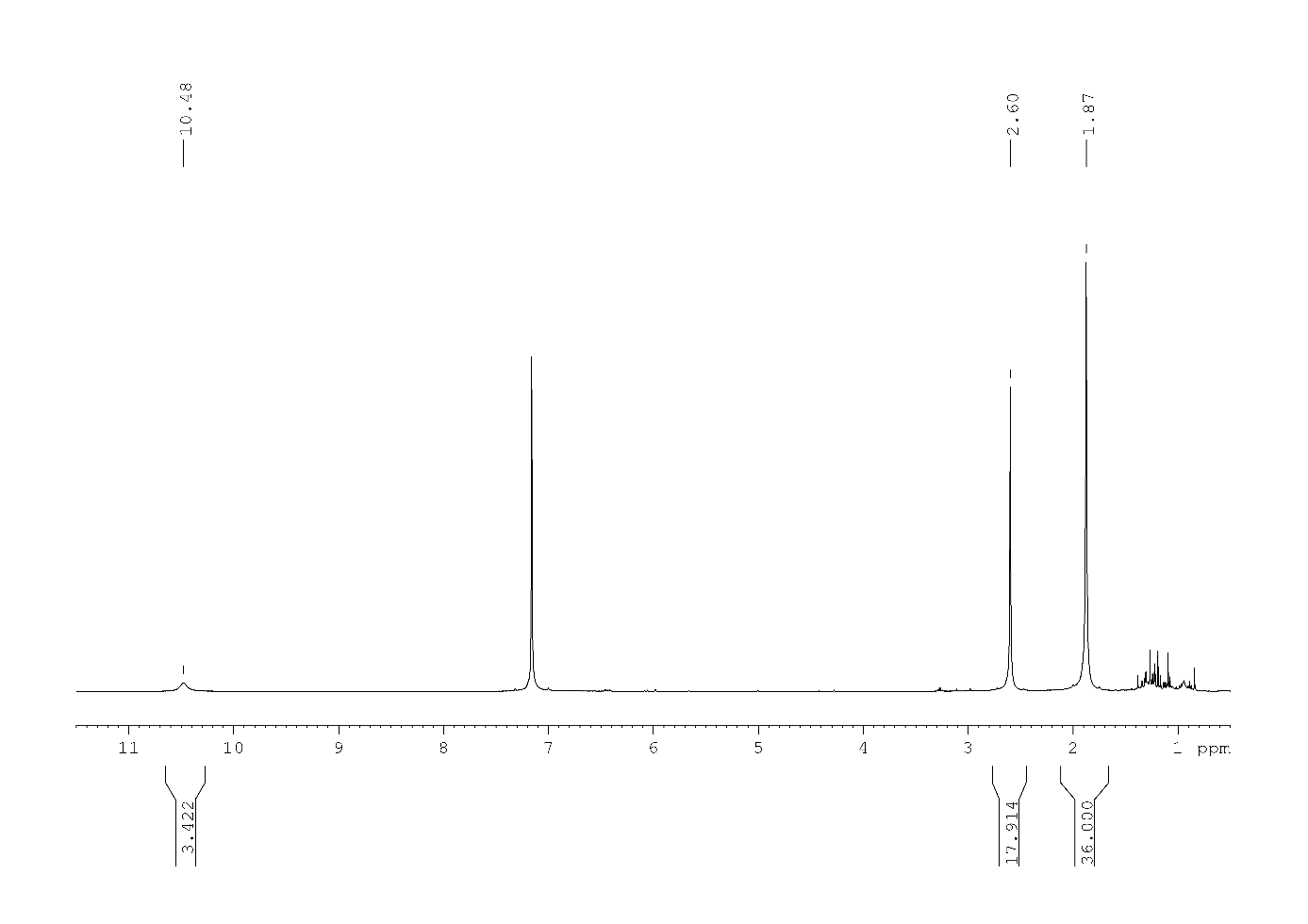
**

**Figure S15.** ^1^H NMR spectrum (500 MHz, C_6_D_6_) for [(Cp’Fe)_2_(μ-η^2^:η^2^-As_2_)(μ-CO)] (**3**).


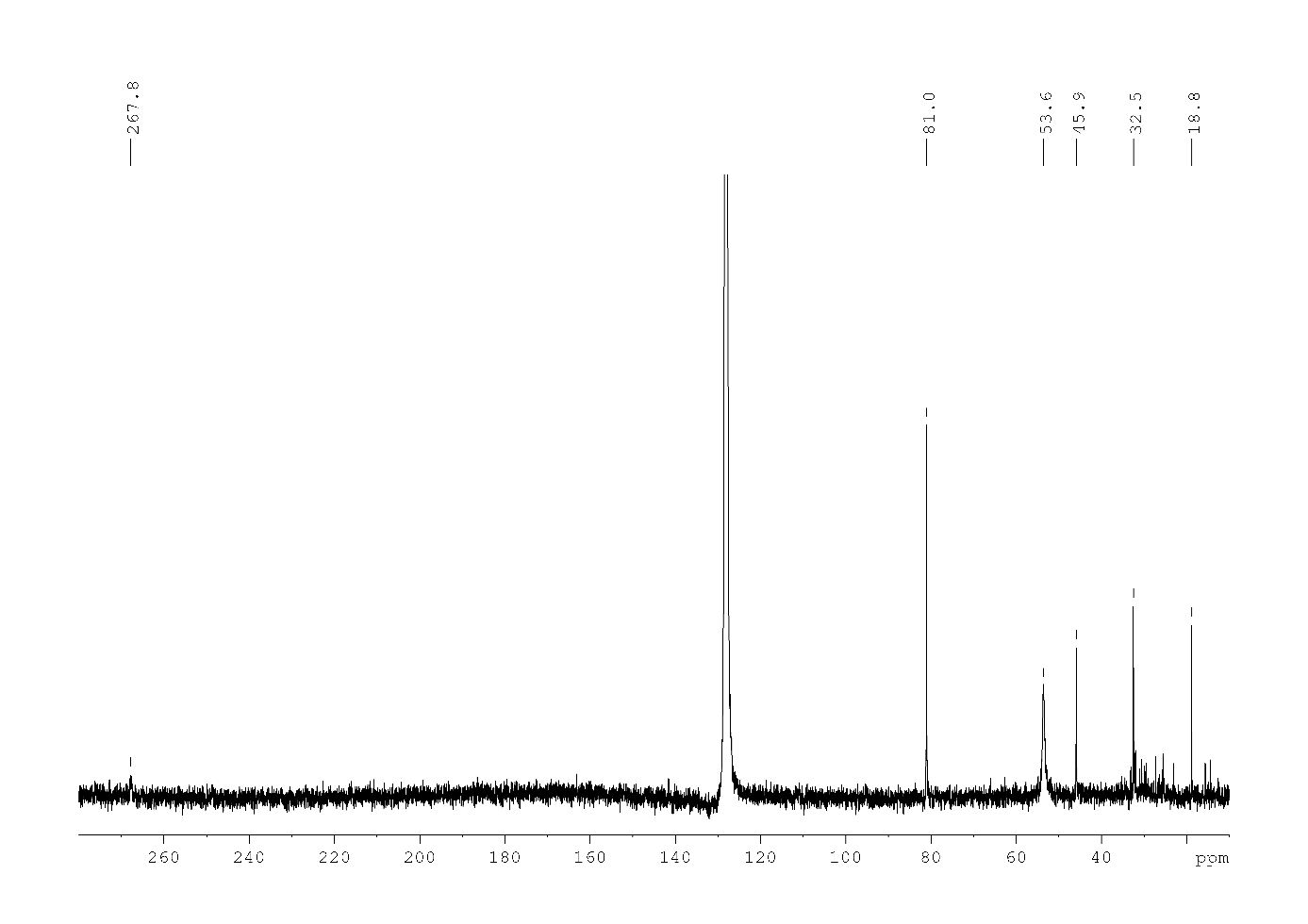


**Figure S16.** ^13^C{^1^H} NMR spectrum (126 MHz, C_6_D_6_) for [(Cp’Fe)_2_(μ-η^2^:η^2^-As_2_)(μ-CO)] (**3**).

**
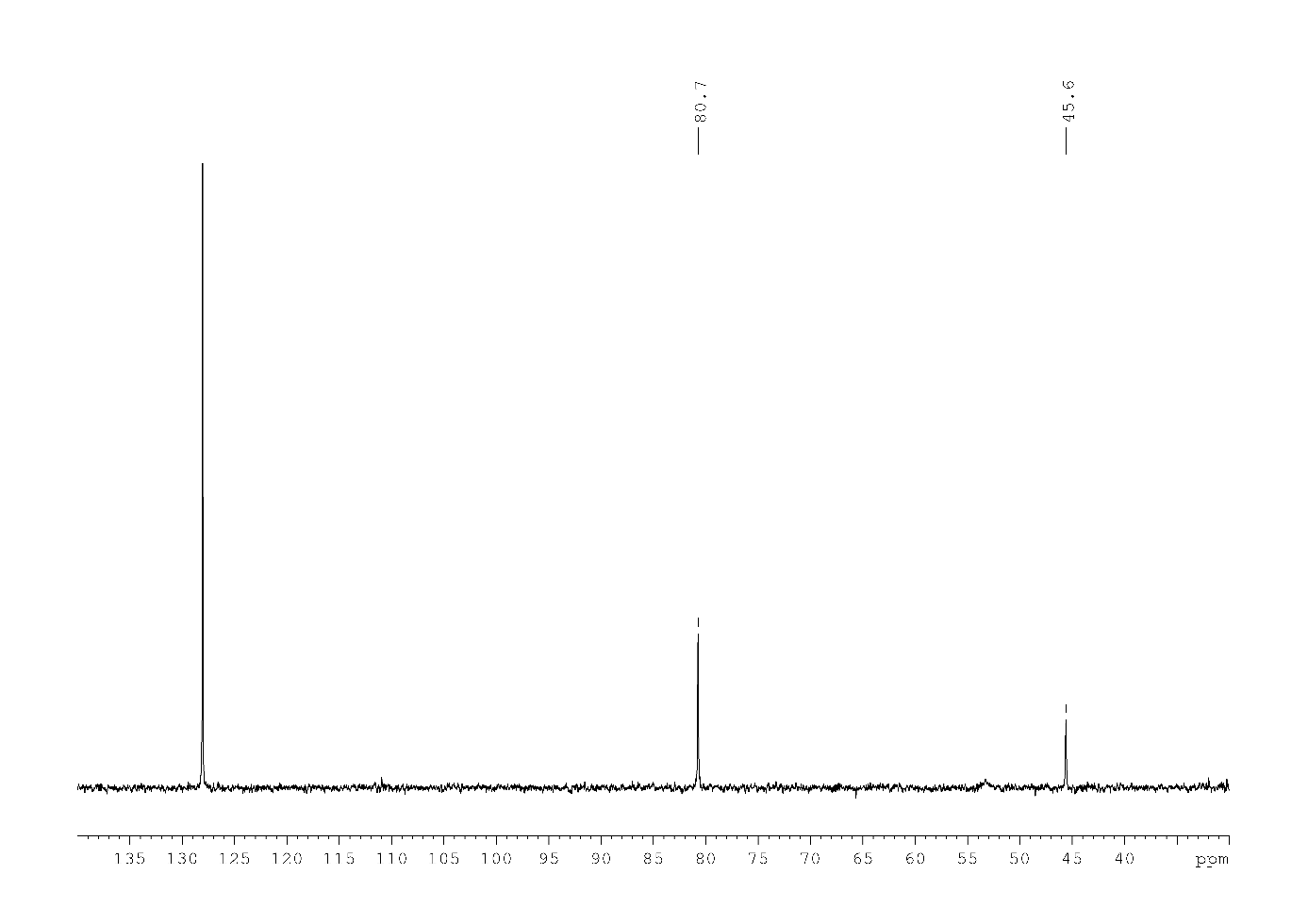
**

**Figure S17.** ^13^C{^1^H} dept-135 NMR spectrum (126 MHz, C_6_D_6_) for [(Cp’Fe)_2_(μ-η^2^:η^2^-As_2_)(μ-CO)] (**3**).


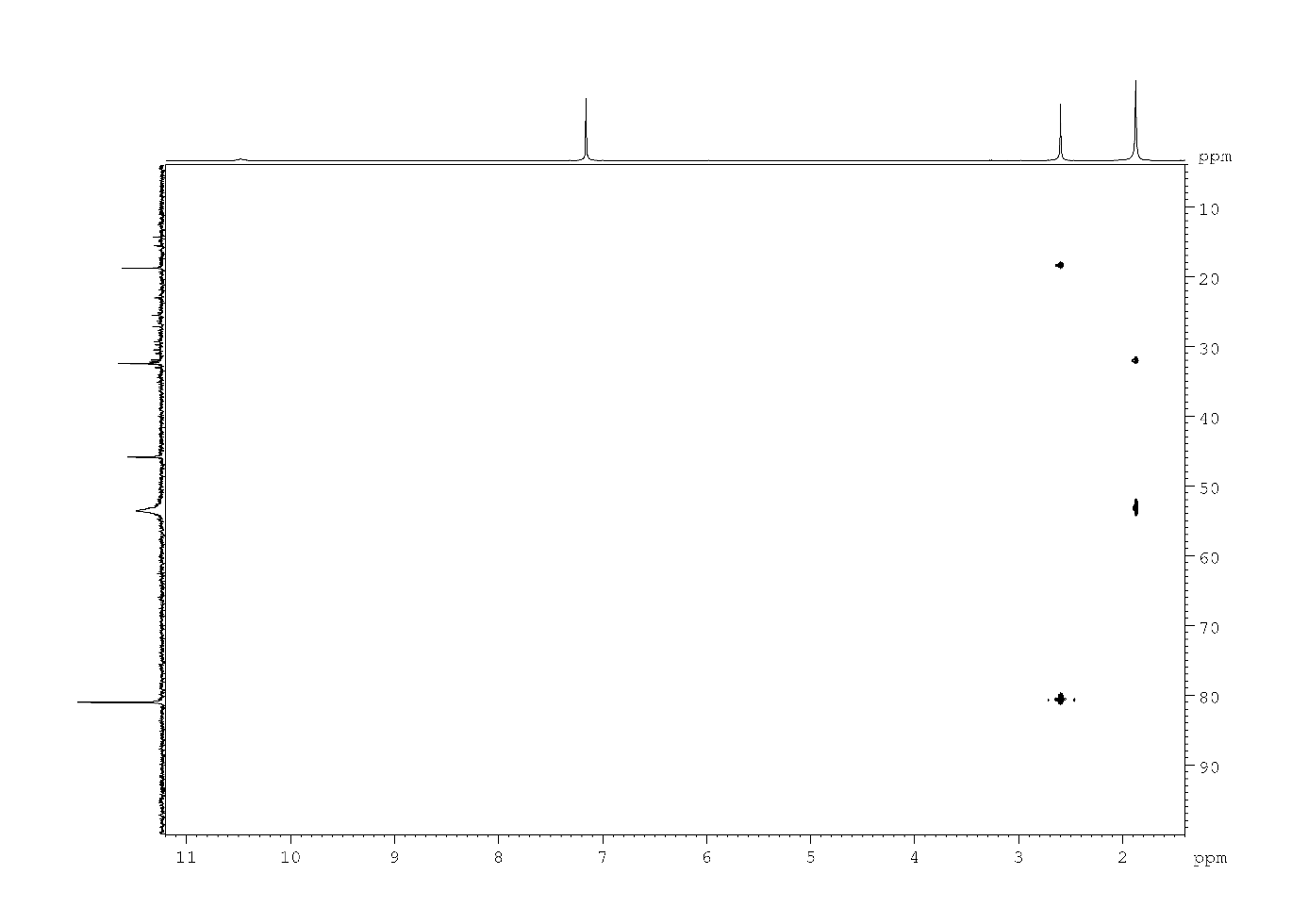


**Figure S18.** ^1^H,^13^C HSQC NMR spectrum (C_6_D_6_) for [(Cp’Fe)_2_(μ-η^2^:η^2^-As_2_)(μ-CO)] (**3**).


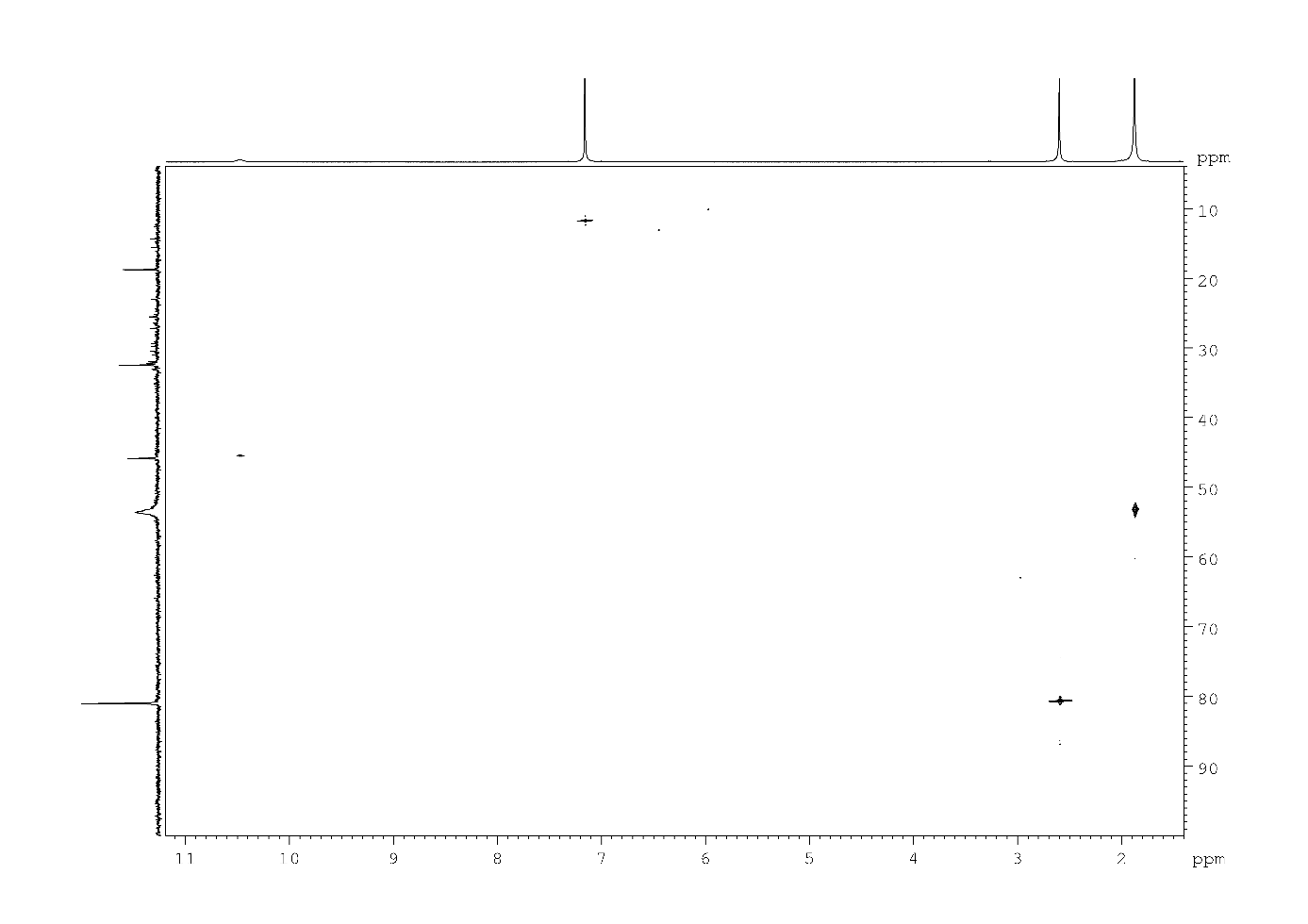


**Figure S19.** ^1^H,^13^C HMBC NMR spectrum (C_6_D_6_) for [(Cp’Fe)_2_(μ-η^2^:η^2^-As_2_)(μ-CO)] (**3**).

- 1. **[(Cp’Fe)_2_(μ-CO)_3_] (6)**

**
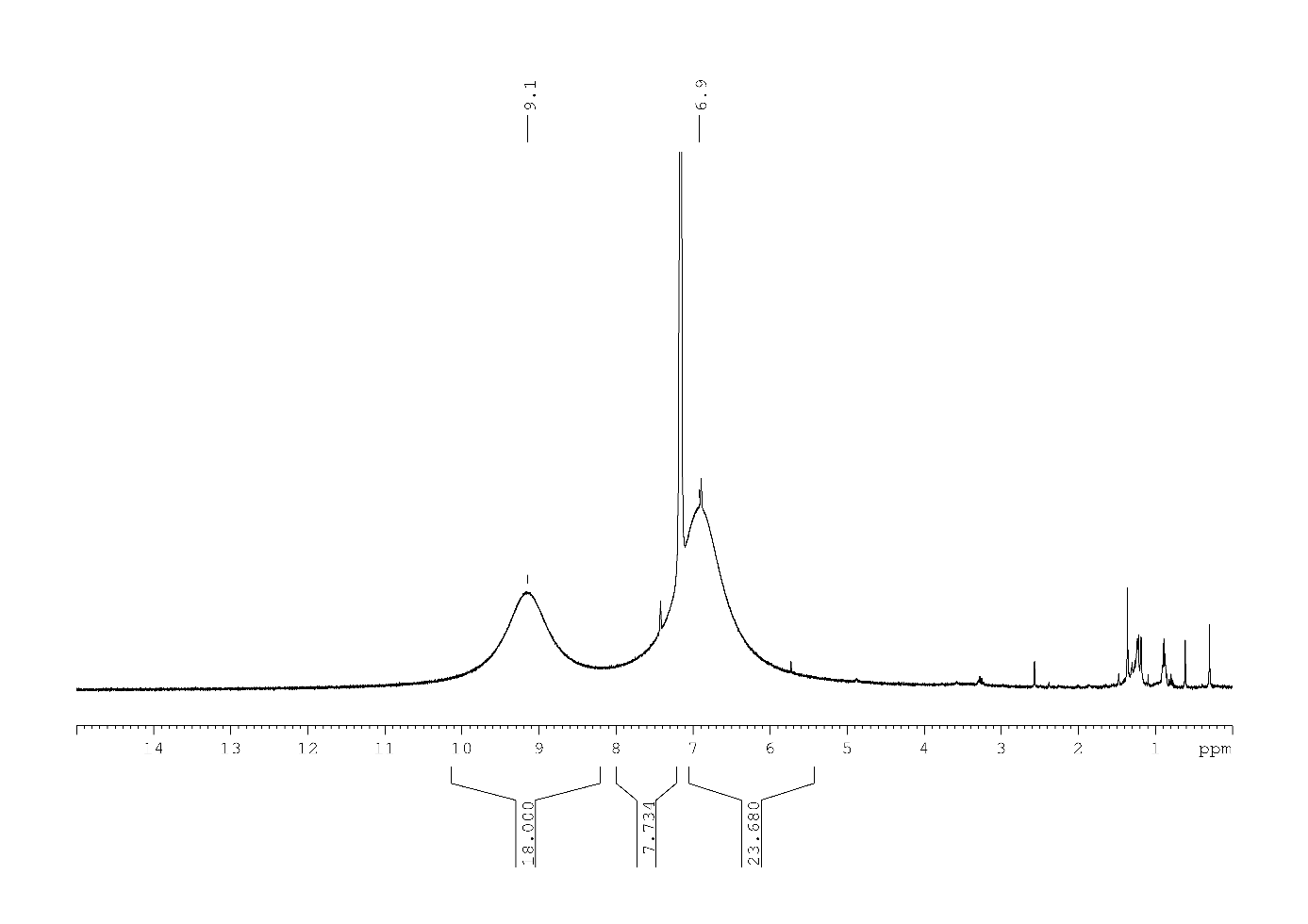
**

**Figure S20.** ^1^H NMR spectrum (300 MHz, C_6_D_6_) for [(Cp’Fe)_2_(μ-CO)_3_] (**6**).

1. **X-ray Crystallography**
   1. **Structure information**

**Table S1.** Solid-state X-ray diffraction data for [(Cp’Fe)_2_(μ-η^2^:η^2^-As_2_)(μ-CO)] (**3**), [(Cp’Fe)_2_(μ-η^2^:η^2^-As_2_)] (**4**), [(Cp’Fe)_3_(μ-η^4^:η^4^:η^4^-As_6_)] (**5**) and [(Cp’Fe)_2_(μ-CO)_3_] (**6**).

|  | [(Cp’Fe)_2_(μ-η^2^:η^2^-As_2_)(μ-CO)] (3) | [(Cp’Fe)_2_(μ-η^2^:η^2^-As_2_)] (4) | [(Cp’Fe)_3_(μ-η^4^:η^4^:η^4^-As_6_)] (5) | [(Cp’Fe)_2_(μ-CO)_3_] (6) |
| --- | --- | --- | --- | --- |
| Chemical formula | C_35_H_58_OAs_2_Fe_2_ | C_34_H_58_As_2_Fe_2_ | C_51_H_87_As_6_Fe_3_ | C_37_H_58_Fe_2_O_3_ |
| Formula mass / g/mol | 756.38 | 728.34 | 1317.27 | 662.53 |
| Crystal system | monoclinic | orthorhombic | triclinic | triclinic |
| *a/*Å | 14.07730(10) | 15.01647(7) | 10.52500(10) | 10.6712(2) |
| *b*/Å | 14.05970(10) | 11.11685(5) | 13.98340(10) | 11.9453(2) |
| *c*/Å | 18.0447(2) | 20.02625(8) | 19.47750(10) | 13.9238(3) |
| *α*/° | 90 | 90 | 93.7850(10) | 90.8917(16) |
| *β*/° | 99.5640(10) | 90 | 101.0410(10) | 90.2559(15) |
| *γ*/° | 90 | 90 | 103.9040(10) | 102.7412(17) |
| Unit cell volume/Å^3^ | 3521.81(5) | 3343.10(2) | 2712.16(4) | 1730.92(6) |
| Temperature/K | 100.0(1) | 99.9(4) | 100.0(4) | 1730.92(6) |
| Space group | *P*2_1_/*n* | *Pbca* | *P*-1 | *P*-1 |
| No. of formula units per unit cell, *Z* | 4 | 4 | 2 | 2 |
| Radiation type | CuK_α_ | CuK_α_ | CuK_α_ | MoK_α_ |
| Absorption coefficient, *μ*/mm^-1^ | 8.867 | 9.294 | 10.647 | 0.871 |
| No. of reflections measured | 96782 | 387168 | 158209 | 20298 |
| No. of independent reflections | 7267 | 3561 | 11510 | 20298 |
| *R_int_* | 0.0436 | 0.0741 | 0.0803 | - |
| Final *R_1_* value (*I* > 2*σ*(*I*)) | 0.0416 | 0.0282 | 0.0359 | 0.0605 |
| Final *wR*_2_(*F*^2^) value (*I* > 2*σ*(*I*)) | 0.1117 | 0.0717 | 0.1000 | 0.1925 |
| Final *R_1_* value (all data) | 0.0441 | 0.0284 | 0.0375 | 0.0662 |
| Final *wR*_2_(*F*^2^) value (all data) | 0.1134 | 0.0719 | 0.1015 | 0.1982 |
| Goodness of fit on *F*^2^ | 1.035 | 1.076 | 1.037 | 1.103 |
| Flack parameter | - | - | - | - |
| Δρ / e Å^-3^ | 3.19 / -0.94 | 0.49 / -0.79 | 0.98 / -1.07 | 1.82 / -0.84 |

- 1. **Refinement special details**
     1. **[(Cp’Fe)_2_(μ-η^2^:η^2^-As_2_)(μ-CO)] (3)**

All H atoms were placed in idealised positions and refined using a riding model, with a common U restrained to be 1.2 (1.5 for methyl groups) times the equivalent isotropic displacement parameter of the parent atom. Methyl groups were treated as rigid bodies and allowed to rotate about the E-CH_3_ bond but not tip. The residual electron density of 3.12 eÅ^-3^ 2.21 Å from C35 arises from a second component of the Fe_2_As_2_(CO) core, which is disordered. However, the second component amounts to less than 10% and could not be refined satisfyingly. A DELU restraint was applied to C35 and O1.

- - 1. **[(Cp’Fe)_2_(μ-η^2^:η^2^-As_2_)] (4)**

All H atoms were placed in idealised positions and refined using a riding model, with a common U restrained to be 1.2 (1.5 for methyl groups) times the equivalent isotropic displacement parameter of the parent atom. Methyl groups were treated as rigid bodies and allowed to rotate about the E-CH_3_ bond but not tip.

- - 1. **[(Cp’Fe)_3_(μ-η^4^:η^4^:η^4^-As_6_)] (5)**

All H atoms were placed in idealised positions and refined using a riding model, with a common U restrained to be 1.2 (1.5 for methyl groups) times the equivalent isotropic displacement parameter of the parent atom. Methyl groups were treated as rigid bodies and allowed to rotate about the E-CH_3_ bond but not tip.


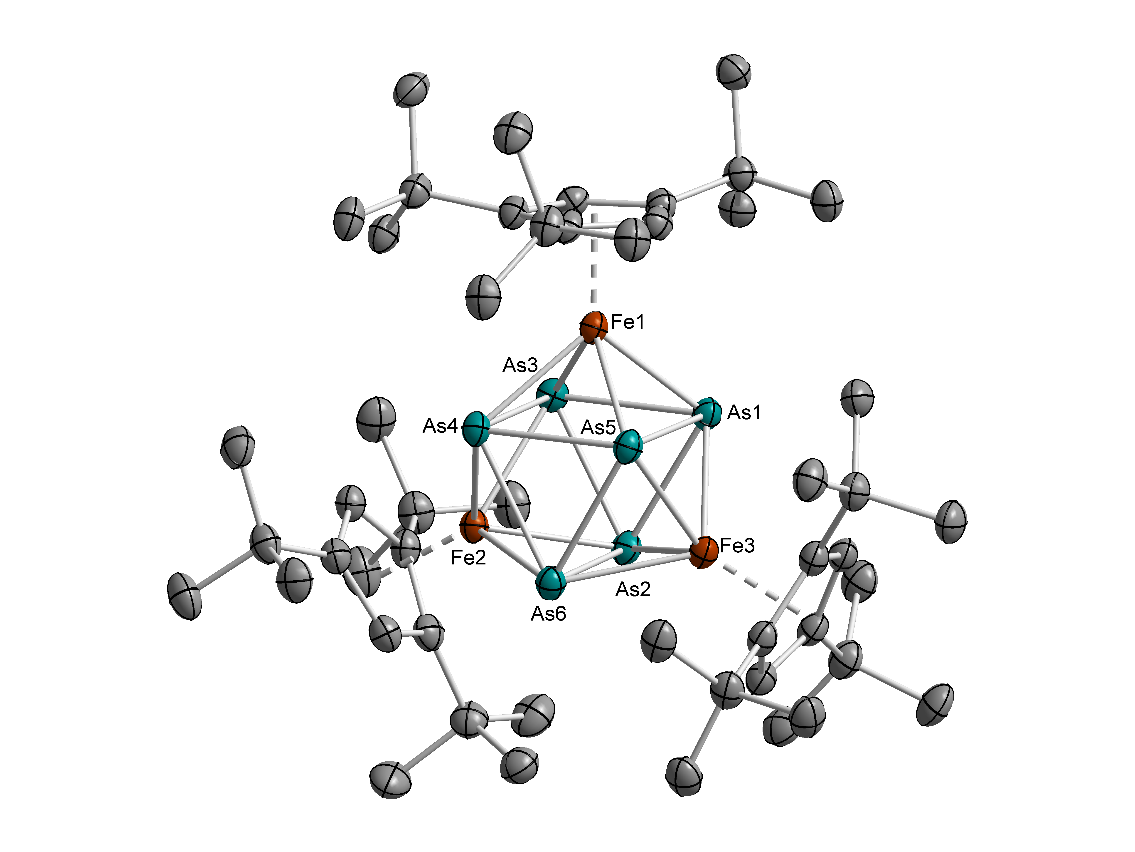


**Figure S21.** Diamond representation of the solid-state molecular structure of [(Cp’Fe)_3_(μ-η^4^:η^4^:η^4^-As_6_)] (**5**). Thermal ellipsoids are drawn at the 50% probability level. Hydrogen atoms are omitted for clarity.

- - 1. **[(Cp’Fe)_2_(μ-CO)_3_] (6)**

All H atoms were placed in idealised positions and refined using a riding model, with a common U restrained to be 1.2 (1.5 for methyl groups) times the equivalent isotropic displacement parameter of the parent atom. Methyl groups were treated as rigid bodies and allowed to rotate about the E-CH_3_ bond but not tip. Refined as a 2-component twin with components of 51% and 49%. High angle data from 2Theta = 64° were not used because of low signal-to-noise ratio. The ratio of maximum / minimum residual electron density is 2.18.


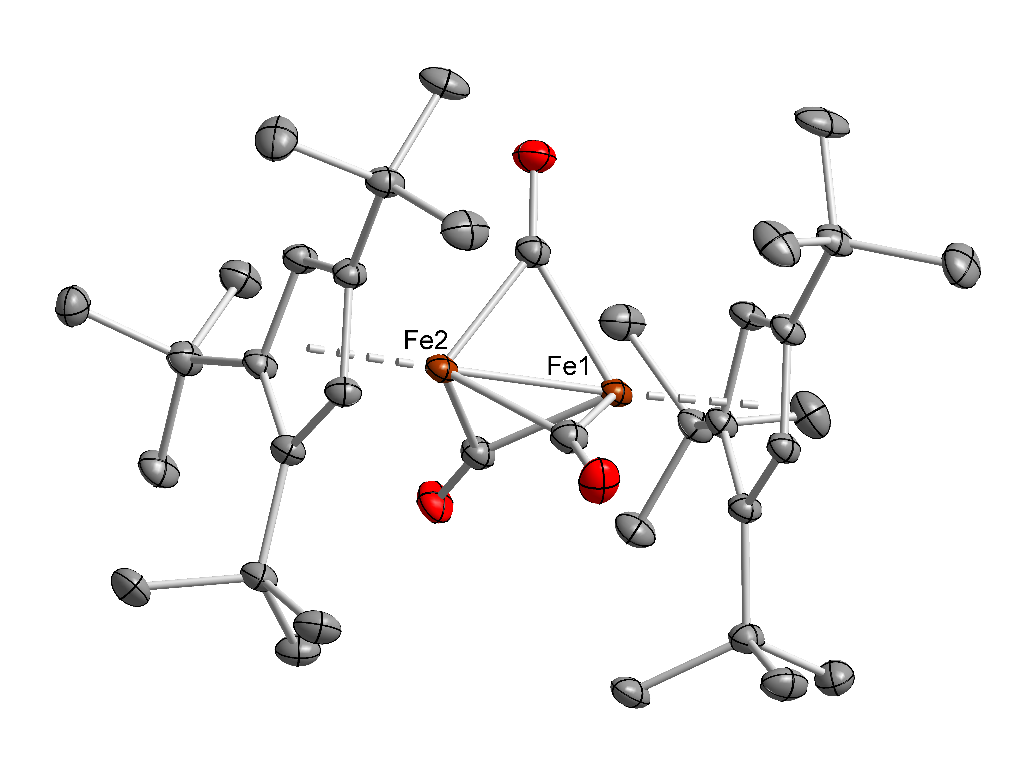


**Figure S22.** Diamond representation of the solid-state molecular structure of [(Cp’Fe)_2_(μ-CO)_3_] (**6**). Thermal ellipsoids are drawn at the 50% probability level. Hydrogen atoms are omitted for clarity. Selected bond distances (Å): Fe1–Cp_cent_ 1.775(1), Fe2–Cp_cent_ 1.778(1), Fe1–Fe2 2.274(1), Fe1–C35(O) 1.940(3), Fe2–C35(O) 1.931(3), Fe1–C36(O) 1.927(3), Fe2–C36(O) 1.959(3), Fe1–C37(O) 1.939(3), Fe2–C37(O) 1.921(3). Angles (°) between planes: (Fe1-Fe2-C35)–(Fe1-Fe2-C36) 68.3(2), (Fe1-Fe2-C35)–(Fe1-Fe2-C37) 53.1(2), (Fe1-Fe2-C36)–(Fe1-Fe2-C37) 58.6(2). Angle (°) between Cp_cent_–Fe1 and Cp_cent_–Fe2 7.4(1).

**3. UV/vis Spectroscopy**

- 1. **[(Cp’Fe)_2_(μ-η^2^:η^2^-P_2_)(μ-CO)] (1)**





**Figure S23.** UV/Vis spectrum of [(Cp’Fe)_2_(μ-η^2^:η^2^-P_2_)(μ-CO)] (**1**) (THF, ambient temperature).

- 1. **[(Cp’Fe)_2_(μ-η^2^:η^2^-As_2_)(μ-CO)] (3)**





**Figure S24.** UV/Vis spectrum of [(Cp’Fe)_2_(μ-η^2^:η^2^-As_2_)(μ-CO)] (**3**) (THF, ambient temperature).

- 1. **[(Cp’Fe)_2_(μ-CO)_3_] (6)**





**Figure S25.** UV/Vis spectrum of [(Cp’Fe)_2_(μ-CO)_3_] (**6**) (n-hexane, ambient temperature).

1. **Cyclic Voltammetry**





**Figure S26.** [(Cp’Fe)_2_(μ-η^2^:η^2^-As_2_)(μ-CO)] (**3**).





**Figure S27.** [(Cp’Fe)_2_(μ-η^2^:η^2^-As_2_)(μ-CO)] (**3**).





**Figure S28.** [(Cp’Fe)_2_(μ-η^2^:η^2^-As_2_)(μ-CO)] (**3**).





**Figure S29.** [(Cp’Fe)_2_(μ-η^2^:η^2^-P_2_)(μ-CO)] (**1**).





**Figure S30.** [(Cp’Fe)_2_(μ-η^2^:η^2^-P_2_)(μ-CO)] (**1**).





**Figure S31.** [(Cp’Fe)_2_(μ-η^2^:η^2^-P_2_)(μ-CO)] (**1**).

1. **Zero-field ^57^Fe Mössbauer spectroscopy**

**Figure S32**. Zero-field ^57^Fe Mössbauer spectrum of **2** (at T = 100 K). Symbols: experimental data. Solid lines: fit with doublets of Lorentzian lines. The black line represents the superposition of the different sub-spectra used in the fit, suggesting the presence of non-equivalent ^57^Fe sites in this specimen. The main signal (red) is attributed to complex **2**, while the additional components (green and dark yellow) can be associated with **1** and an unidentified impurity (presumably **VI**), respectively. The remaining sub-spectrum (orange) revealed $\delta$ and $\Delta E_{Q}$ values similar to those for ferrocene **XII**. The parameters of the fit are summarised in **Table 3** in the main text.

**Figure S33**. Zero-field ^57^Fe Mössbauer spectrum of **4** (at T = 100 K). Symbols: experimental data. Solid lines: fit with doublets of Lorentzian lines. The black line represents the superposition of the different sub-spectra used in the fit, suggesting the presence of non-equivalent ^57^Fe sites in this specimen. The main signals (green and dark yellow) can be associated with **4** (or, alternatively, with **4** and a yet unidentified by-product). The remaining sub-spectrum (orange) revealed $\delta$ and $\Delta E_{Q}$ values similar to those for ferrocene **XII**. The parameters of the fit are summarised in **Table 3** in the main text.

**Figure S34**. Zero-field ^57^Fe Mössbauer spectrum of **5** (at T = 100 K). Symbols: experimental data. Solid lines: fit with doublets of Lorentzian lines. The black line represents the superposition of the different sub-spectra used in the fit, suggesting the presence of non-equivalent ^57^Fe sites in this specimen. The main signal (red) is attributed to complex **XI**. The additional components (green and dark yellow) can be associated with **4** (or, alternatively, with **4** and a yet unidentified by-product), while the formation of compound **3** was indicated by the brownish line in the figure. The remaining sub-spectrum (orange) revealed $\delta$ and $\Delta E_{Q}$ values similar to those for ferrocene **XII**. The parameters of the fit are summarised in **Table 3** in the main text.

**Figure S35**. Zero-field ^57^Fe Mössbauer spectrum of **XI** (at T = 100 K). Symbols: experimental data. Solid lines: fit with a doublet of Lorentzian lines. The parameters of the fit are summarised in **Table 3** in the main text.

1. **Computational Studies**

**6.1 Computational details**

All DFT calculations were carried out with the Gaussian 16 suite of programs.^[1]^ Geometries were fully optimized in the gas phase without symmetry constraints using the B3PW91 functional.^[2, 3]^ The nature of the extrema was verified by analytical frequency calculations. The calculations of electronic energies and enthalpies of the extrema of the potential energy surface (minima and transition states) were performed at the same level of theory as the geometry optimizations. Intrinsic reaction coordinate (IRC) calculations were performed to confirm the connections of the optimized transition states. Iron and Iodine atoms were treated with a Stuttgart effective core potential augmented with a polarization function (ζ_f_ = 2,462 and ζ_d_ = 0,730 respectively for Fe and I atoms).^[4, 5]^ For the other elements (H, C, As, F and O), Pople’s double-ζ basis set 6-31G(d,p) was used.^[6, 7]^ The electronic charges (at the DFT level) were computed using the natural population analysis technique.^[8]^ Dispersion corrections were treated with the D3 version of Grimme’s dispersion with Becke-Johnson damping.^[9]^

**Table S2.** Comparison of selected distances (in Å) for complexes **1** and **3**: experimental values *vs.* the most stable DFT-derived structures for each spin multiplicity, with and without dispersion corrections.

|  | exp | 2S +1 = 1, closed shell | | 2S +1 = 1, open shell | | 2S +1 = 3 | | 2S +1 = 5 | |
| --- | --- | --- | --- | --- | --- | --- | --- | --- | --- |
| **Complex 1** |  | **without dispersion** | | | | | | | |
| ΔrG (kcal/mol) | - | 15.3 | | -0.6 | | 4.3 | | 0.0 | |
| ΔrH (kcal/mol) | - | 11.6 | | -0.2 | | 2.5 | | 0.0 | |
|  |  | Distance | Δd_(theo-exp)_ | Distance | Δd_(theo-exp)_ | Distance | Δd_(theo-exp)_ | Distance | Δd_(theo-exp)_ |
| d(Fe,Fe) | 2.394 | 2.379 | -0.02 | 2.738 | 0.34 | 2.473 | 0.08 | 2.547 | 0.15 |
| d(P,P) | 2.064 | 2.057 | -0.01 | 2.13 | 0.07 | 2.094 | 0.03 | 2.069 | 0.00 |
| d(Fe,P)* | 2.291 | 2.29 | 0.00 | 2.307 | 0.02 | 2.27 | -0.02 | 2.33 | 0.04 |
| d(Fe,C=O)* | 1.920 | 1.872 | -0.05 | 2.016 | 0.10 | 1.906 | -0.01 | 1.9 | -0,02 |
|  |  | **with dispersion** | | | | | | | |
| Δ_r_G (kcal/mol) | - | 8.2 | | 1.5 | | 0.1 | | 0.0 | |
| Δ_r_H (kcal/mol) | - | 4.7 | | 1.0 | | -1.5 | | 0.0 | |
|  |  | Distance | Δd_(theo-exp)_ | Distance | Δd_(theo-exp)_ | Distance | Δd_(theo-exp)_ | Distance | Δd_(theo-exp)_ |
| d(Fe,Fe) | 2.394 | 2.344 | -0.05 | 2.568 | 0.17 | 2.431 | 0.04 | 2.496 | 0.10 |
| d(P,P) | 2.064 | 2.057 | -0.01 | 2,095 | 0.03 | 2,095 | 0.03 | 2,072 | 0.01 |
| d(Fe,P)* | 2.291 | 2.27975 | -0.01 | 2,2605 | -0.03 | 2.261 | -0.03 | 2.313 | 0.02 |
| d(Fe,C=O)* | 1.920 | 1.863 | -0.06 | 1,8915 | -0.03 | 1.892 | -0.03 | 1.889 | -0.03 |

|  | exp | 2S +1 = 1, closed shell | | 2S +1 = 1, open shell | | 2S +1 = 3 | | 2S +1 = 5 | |
| --- | --- | --- | --- | --- | --- | --- | --- | --- | --- |
| **Complex 3** |  | **without dispersion** | | | | | | | |
| Δ_r_G (kcal/mol) | - | 16.8 | | -1.4 | | 5.2 | | 0.0 | |
| Δ_r_H (kcal/mol) | - | 13.4 | | -1.0 | | 2.9 | | 0.0 | |
|  |  | Distance | Δd_(theo-exp)_ | Distance | Δd_(theo-exp)_ | Distance | Δd_(theo-exp)_ | Distance | Δd_(theo-exp)_ |
| d(Fe,Fe) | 2.410 | 2.396 | -0.01 | 2.756 | 0,35 | 2.491 | 0.08 | 2.564 | 0.15 |
| d(As,As)) | 2.288 | 2.264 | -0.02 | 2.357 | 0,07 | 2.313 | 0.03 | 2.289 | 0.00 |
| d(Fe,As)* | 2.397 | 2.39 | -0.01 | 2.399 | 0,00 | 2.36 | -0.04 | 2.42 | 0.02 |
| d(Fe,C=O)* | 1.940 | 1.871 | -0.07 | 2.01 | 0,07 | 1.9035 | -0.04 | 1.904 | -0.04 |
|  |  | **with dispersion** | | | | | | | |
| Δ_r_G (kcal/mol) | - | 9.9 | | 0.6 | | -0.1 | | 0.0 | |
| Δ_r_H (kcal/mol) | - | 6.6 | | 0.5 | | -1.2 | | 0.0 | |
|  |  | Distance | Δd_(theo-exp)_ | Distance | Δd_(theo-exp)_ | Distance | Δd_(theo-exp)_ | Distance | Δd_(theo-exp)_ |
| d(Fe,Fe) | 2.410 | 2.36 | -0.05 | 2.589 | 0.18 | 2.447 | 0.04 | 2.511 | 0.10 |
| d(As,As)) | 2.288 | 2.265 | -0.02 | 2.364 | 0.08 | 2.318 | 0.03 | 2.293 | 0.01 |
| d(Fe,As)* | 2.397 | 2.372 | -0.02 | 2.347 | -0.05 | 2.347 | -0.05 | 2.399 | 0.00 |
| d(Fe,C=O)* | 1.940 | 1.861 | -0.08 | 1.919 | -0.02 | 1.889 | -0.05 | 1.887 | -0.05 |

* averaged values

**Table S3.** Comparison of selected distances (in Å) for complexes **2** and **4**: experimental values *vs.* the most stable DFT-derived structures for each spin multiplicity, with and without dispersion corrections.

|  | exp | 2S +1 = 1, closed shell | | 2S +1 = 1, open shell | | 2S +1 = 3 | | 2S +1 = 5 | |
| --- | --- | --- | --- | --- | --- | --- | --- | --- | --- |
| **Complex 2** |  | **without dispersion** | | | | | | | |
| Δ_r_G (kcal/mol) | - | -1.1 | | -16.6 | | -11.5 | | 0.0 | |
| Δ_r_H (kcal/mol) | - | -3.4 | | -15.7 | | -10.4 | | 0.0 | |
|  |  | Distance | Δd_(theo-exp)_ | Distance | Δd_(theo-exp)_ | Distance | Δd_(theo-exp)_ | Distance | Δd_(theo-exp)_ |
| d(Fe,Fe) | 2.501 | 2.498 | 0.00 | 2.739 | 0.24 | 2.545 | 0.04 | 2.533 | 0.03 |
| d(P,P) | 3.383 | 3.339 | -0.04 | 3.372 | -0.01 | 3.421 | 0.04 | 3.521 | 0.14 |
| d(Fe,P)* | 2.104 | 2.085 | -0.02 | 2.173 | 0.07 | 2.163 | 0.06 | 2.169 | 0.07 |
|  |  | **with dispersion** | | | | | | | |
| Δ_r_G (kcal/mol) | - | -4.6 | | -5.5 | | -10.1 | | 0.0 | |
| Δ_r_H (kcal/mol) | - | -7.8 | | -8.3 | | -9.8 | | 0.0 | |
|  |  | Distance | Δd_(theo-exp)_ | Distance | Δd_(theo-exp)_ | Distance | Δd_(theo-exp)_ | Distance | Δd_(theo-exp)_ |
| d(Fe,Fe) | 2.501 | 2.461 | -0.04 | 2.462 | -0.04 | 2.482 | -0.02 | 2.491 | -0.01 |
| d(P,P) | 3.383 | 3.353 | -0.03 | 3.361 | -0.02 | 3.386 | 0.00 | 3.523 | 0.14 |
| d(Fe,P)* | 2.104 | 2.080 | -0.02 | 2.083 | -0.02 | 2.148 | 0.04 | 2.158 | 0.05 |
|  |  |  |  |  |  |  |  |  |  |
| **Complex 4** |  | **without dispersion** | | | | | | | |
| Δ_r_G (kcal/mol) | - | 5.1 | | -16.3 | | -1.7 | | 0.0 | |
| Δ_r_H (kcal/mol) | - | 0.1 | | -16.5 | | -4.1 | | 0.0 | |
|  |  | Distance | Δd_(theo-exp)_ | Distance | Δd_(theo-exp)_ | Distance | Δd_(theo-exp)_ | Distance | Δd_(theo-exp)_ |
| d(Fe,Fe) | 2.567 | 2.57 | 0.00 | 2.863 | 0.30 | 2.649 | 0.08 | 2.593 | 0.03 |
| d(As,As)) | 3.617 | 3.534 | -0.08 | 3.525 | -0.09 | 3.583 | -0.03 | 3.686 | 0.07 |
| d(Fe,As)* | 2.218 | 2.185 | -0.03 | 2.271 | 0.05 | 2.229 | 0.01 | 2.254 | 0.04 |
|  |  | **with dispersion** | | | | | | | |
| ΔrG (kcal/mol) | - | -0.9 | | -2,6 | | -4,6 | | 0,0 | |
| ΔrH (kcal/mol) | - | -3.9 | | -5,4 | | -5,6 | | 0,0 | |
|  |  | Distance | Δd_(theo-exp)_ | Distance | Δd_(theo-exp)_ | Distance | Δd_(theo-exp)_ | Distance | Δd_(theo-exp)_ |
| d(Fe,Fe) | 2.567 | 2.531 | -0,04 | 2.521 | -0.05 | 2.58 | 0,01 | 2.525 | -0.04 |
| d(As,As)) | 3.617 | 3.542 | -0,08 | 3.572 | -0.04 | 3.601 | -0,02 | 3.701 | 0.08 |
| d(Fe,As)* | 2.218 | 2.177 | -0,04 | 2.186 | -0.03 | 2.216 | 0,00 | 2.24 | 0.02 |


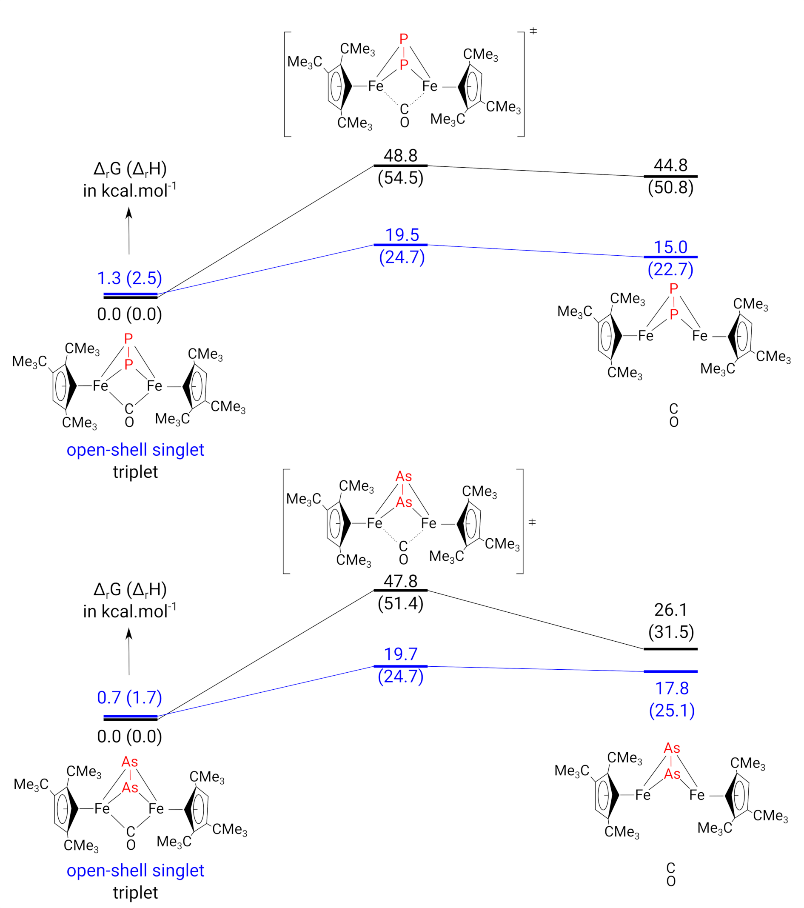


**Figure S36.** Computed profile (in kcal/mol) for the CO release from complexes **1** and **3**.

**
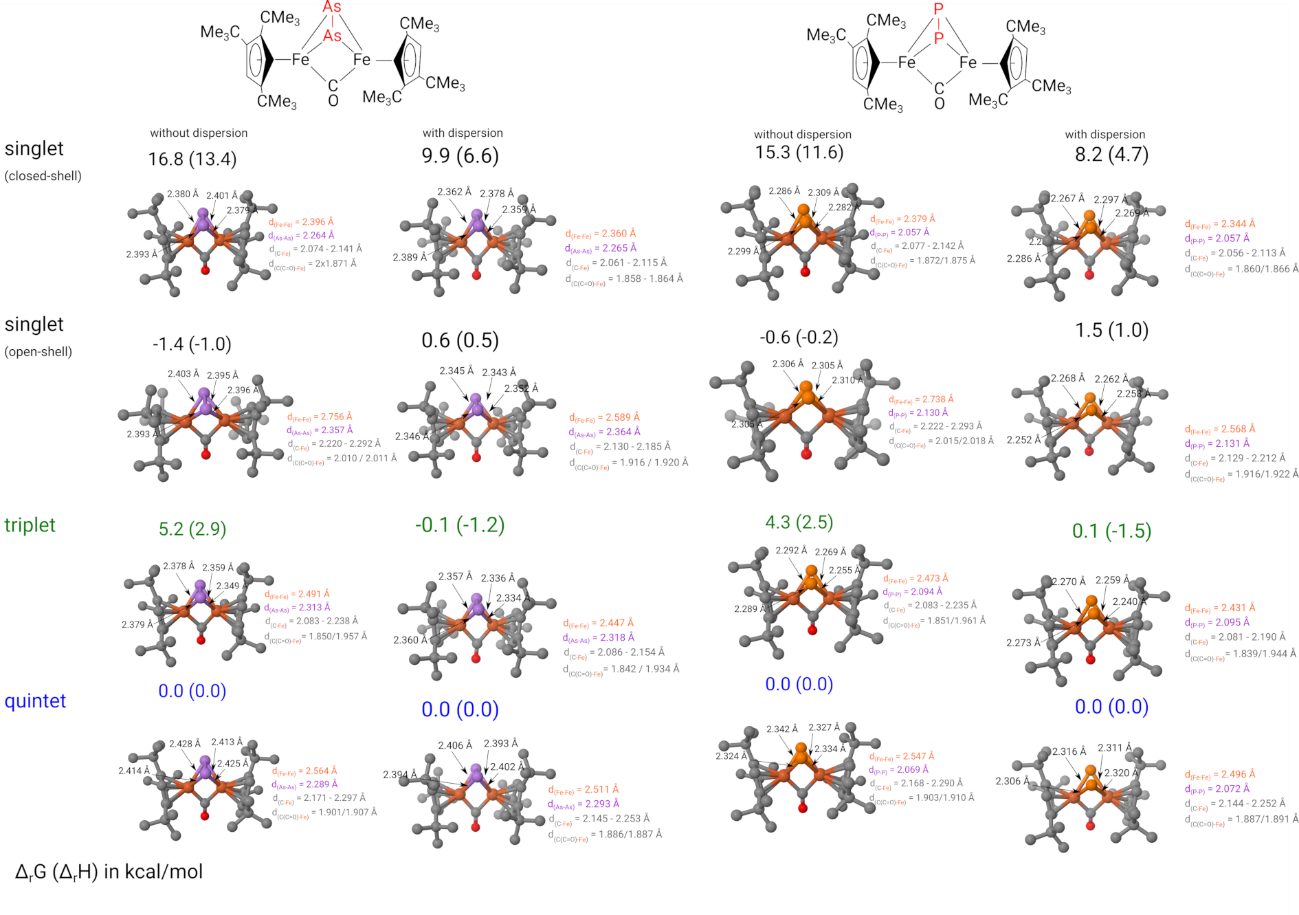
**

**Figure S37.** Dispersion effects on the computed structures and relative stabilities of complexes **1** to **4**.

**
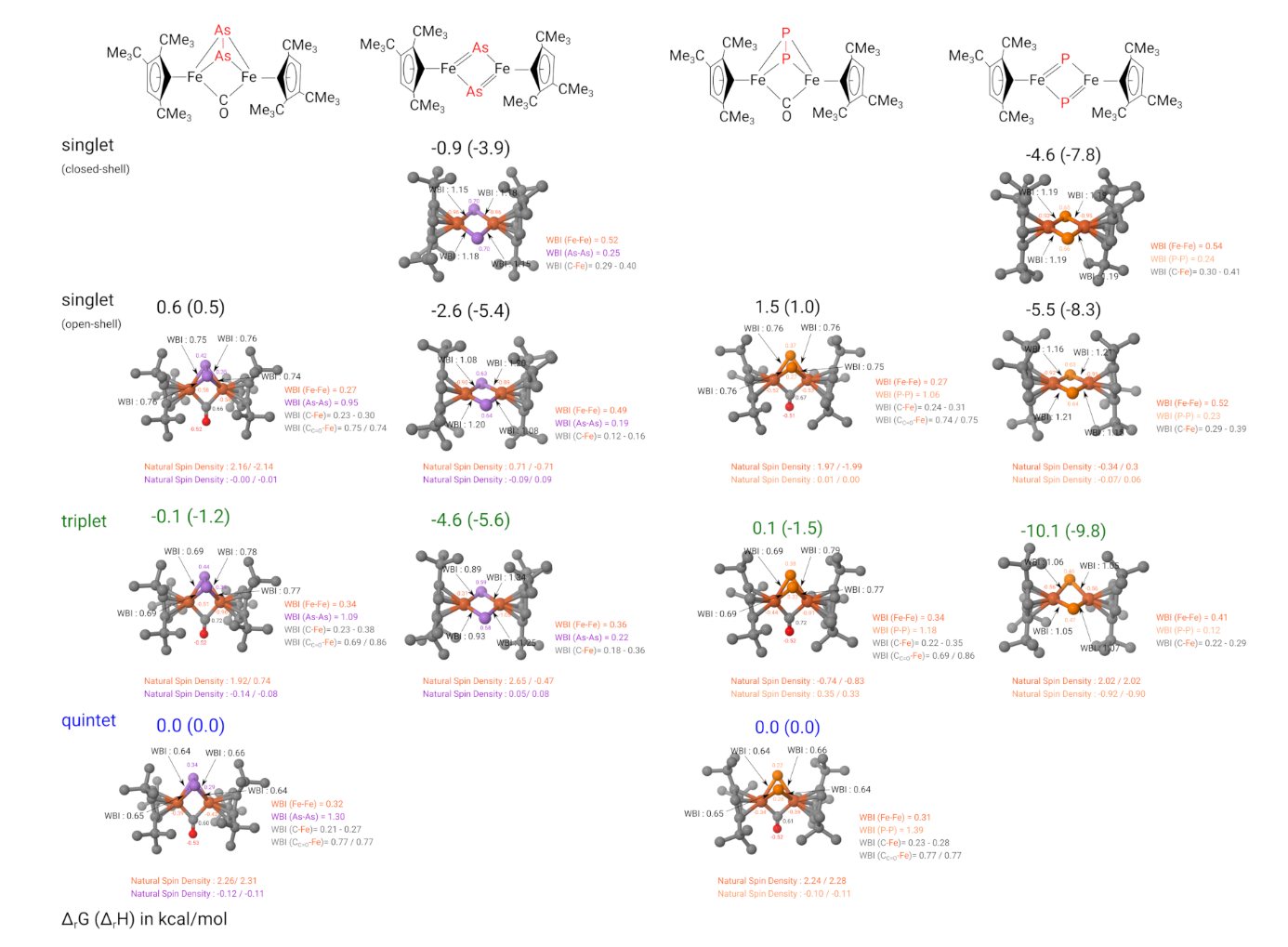
**

**Figure S38.** Wiberg bond indexes (WBI) and natural spin densities of complexes **1** to **4.**

**
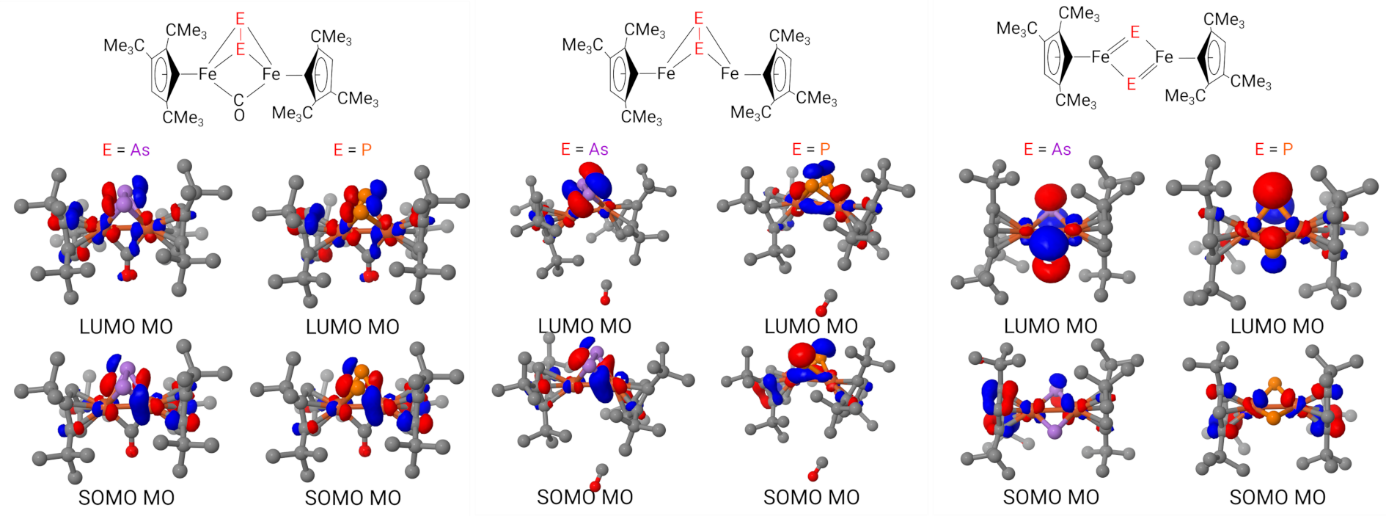
**

**Figure S39.** SOMO and LUMO orbitals for complexes **1** to **4**.

**
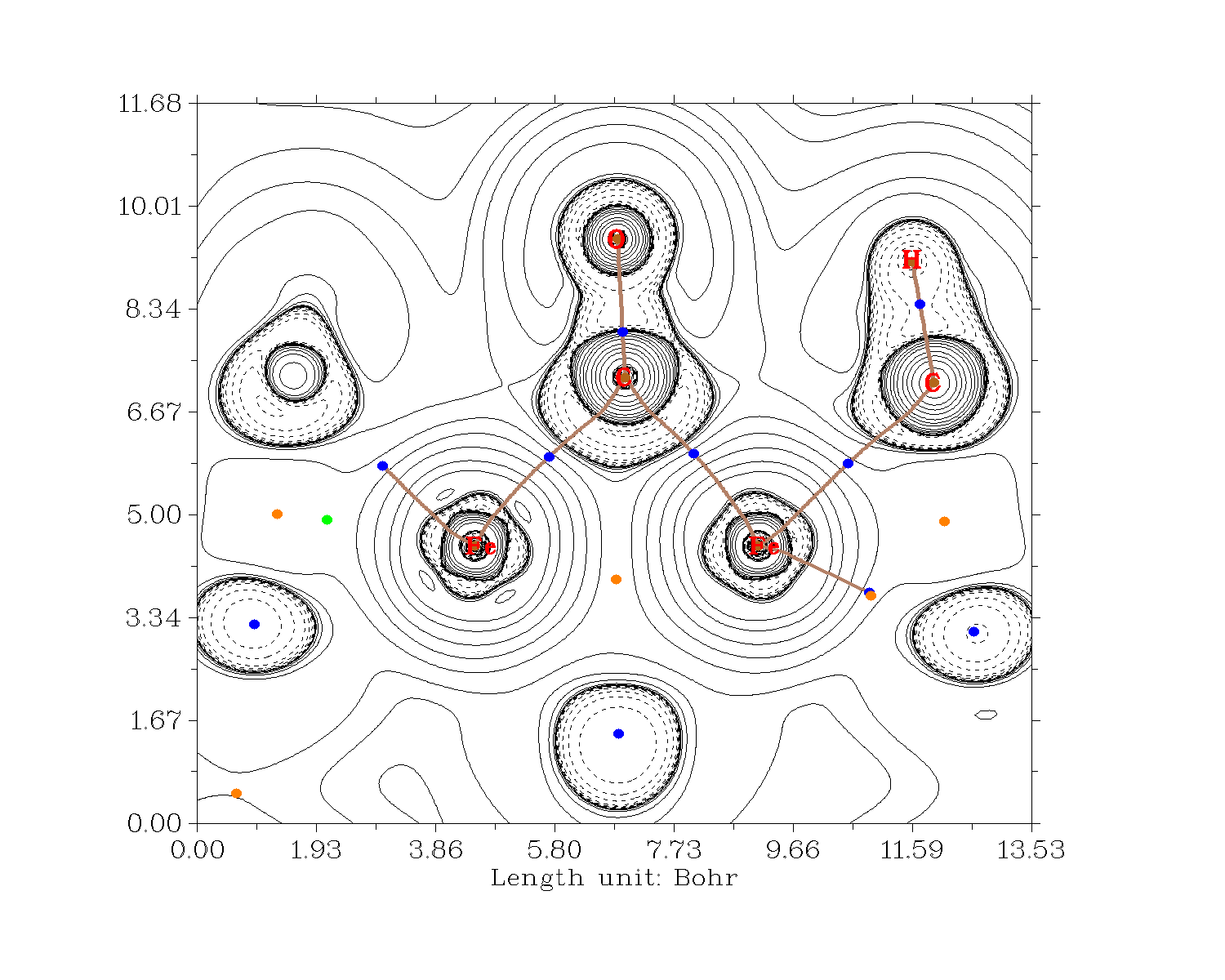
**

**Figure S39.** QTAIM for complex **1** on the Fe-CO-Fe plane

**
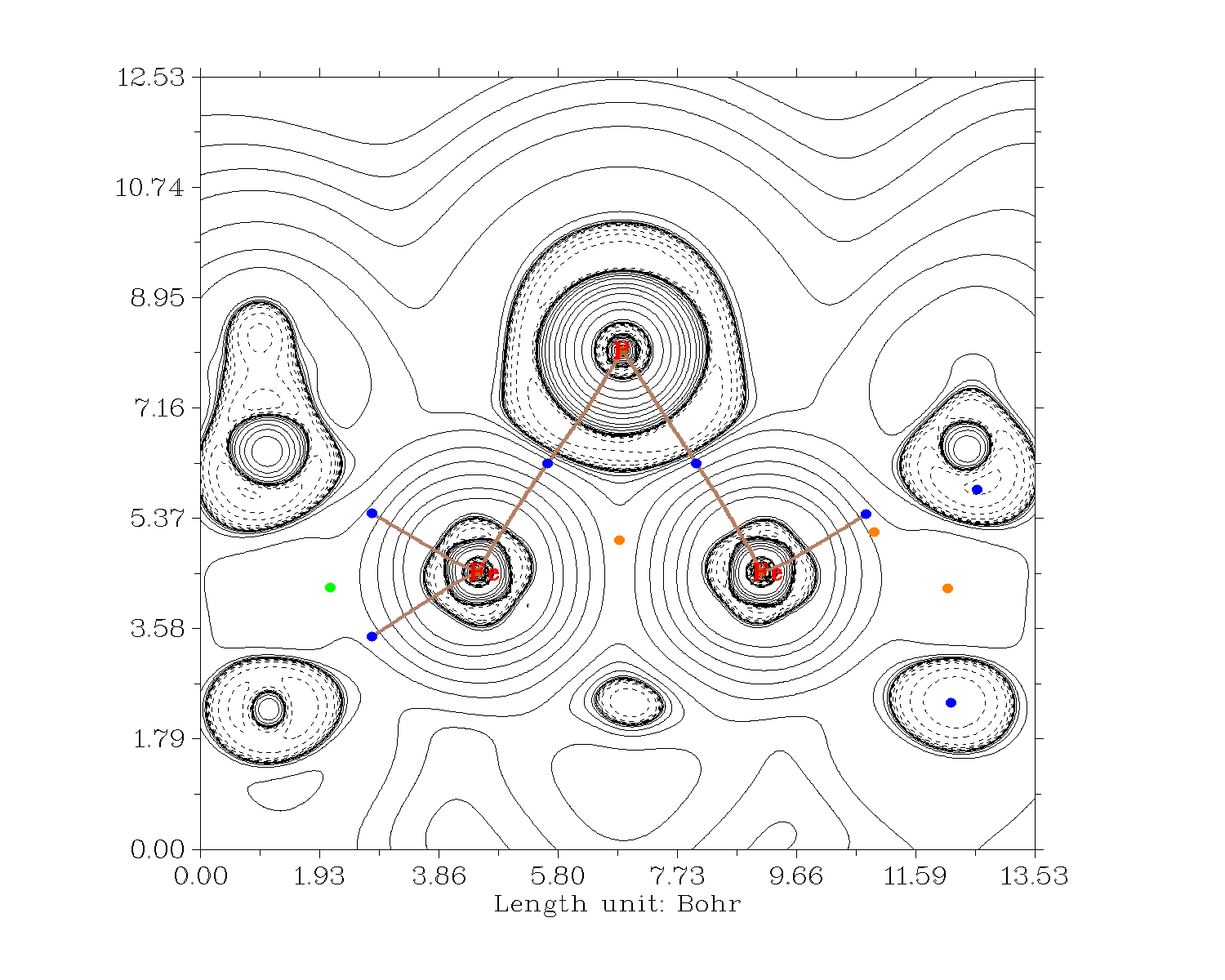
**

**Figure S40.** QTAIM for complex **1** on the Fe-P-Fe plane

**
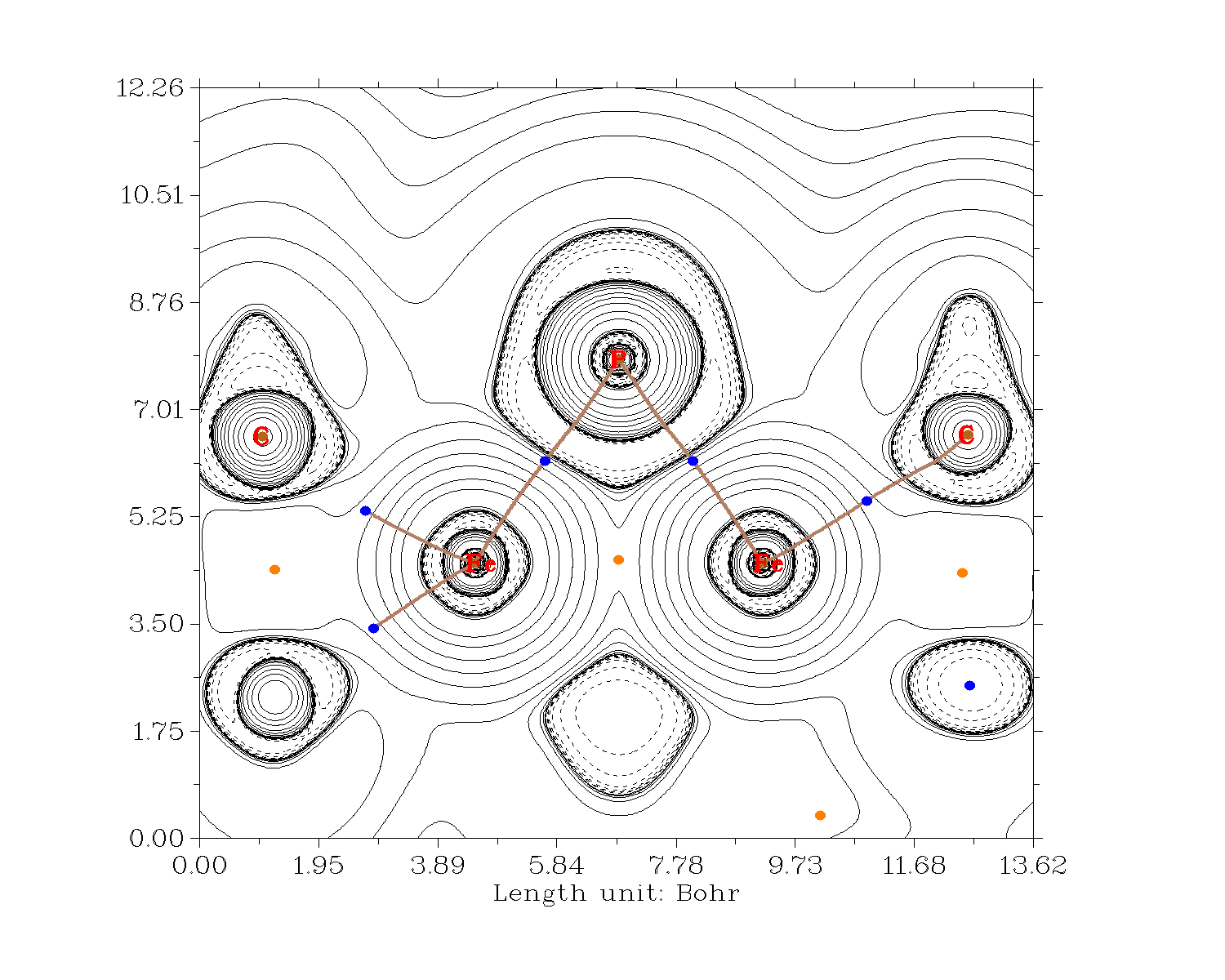
**

**Figure S41.** QTAIM for complex **2** on the Fe-P-Fe plane

**
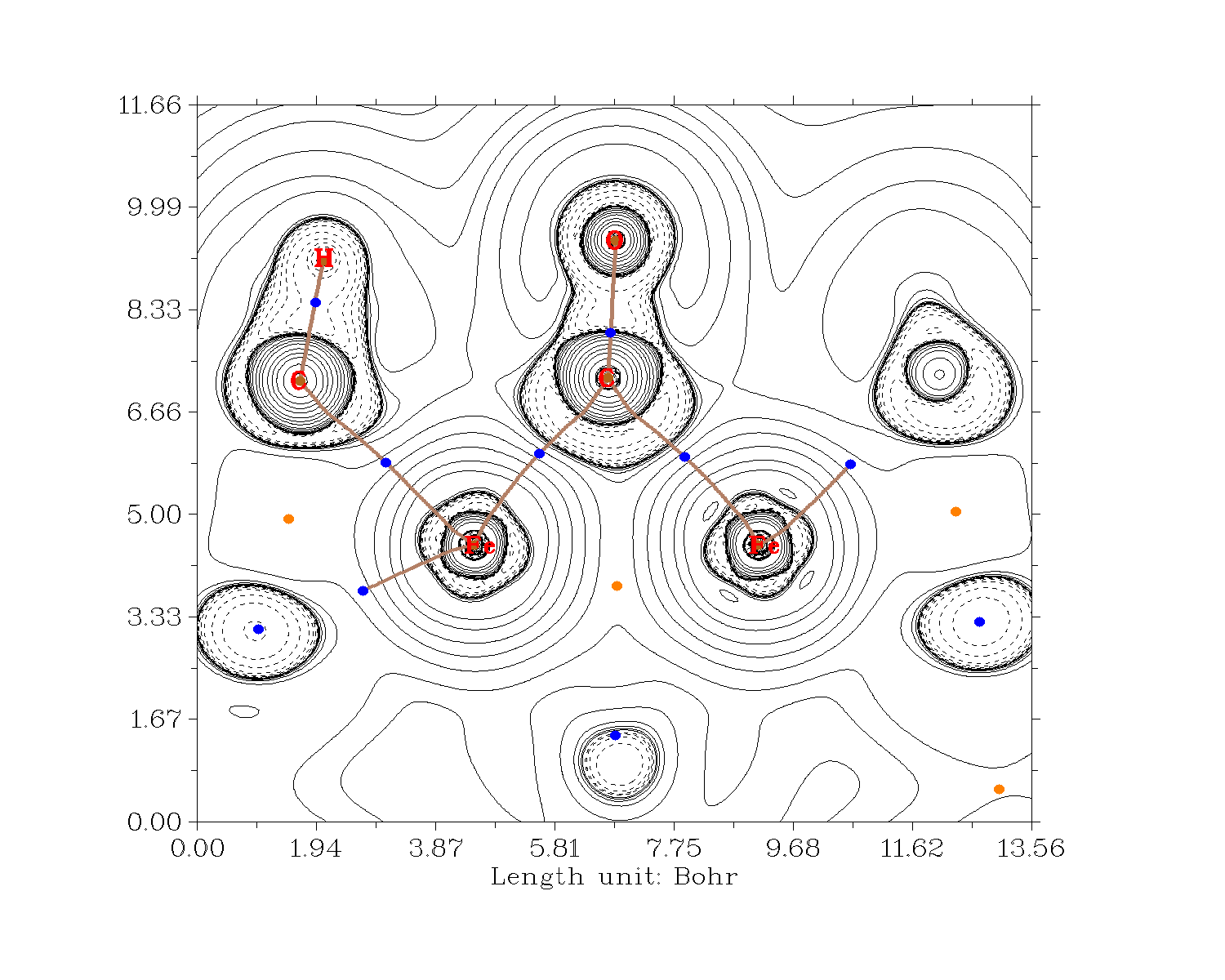
**

**Figure S42.** QTAIM for complex **3** on the Fe-CO-Fe plane

**
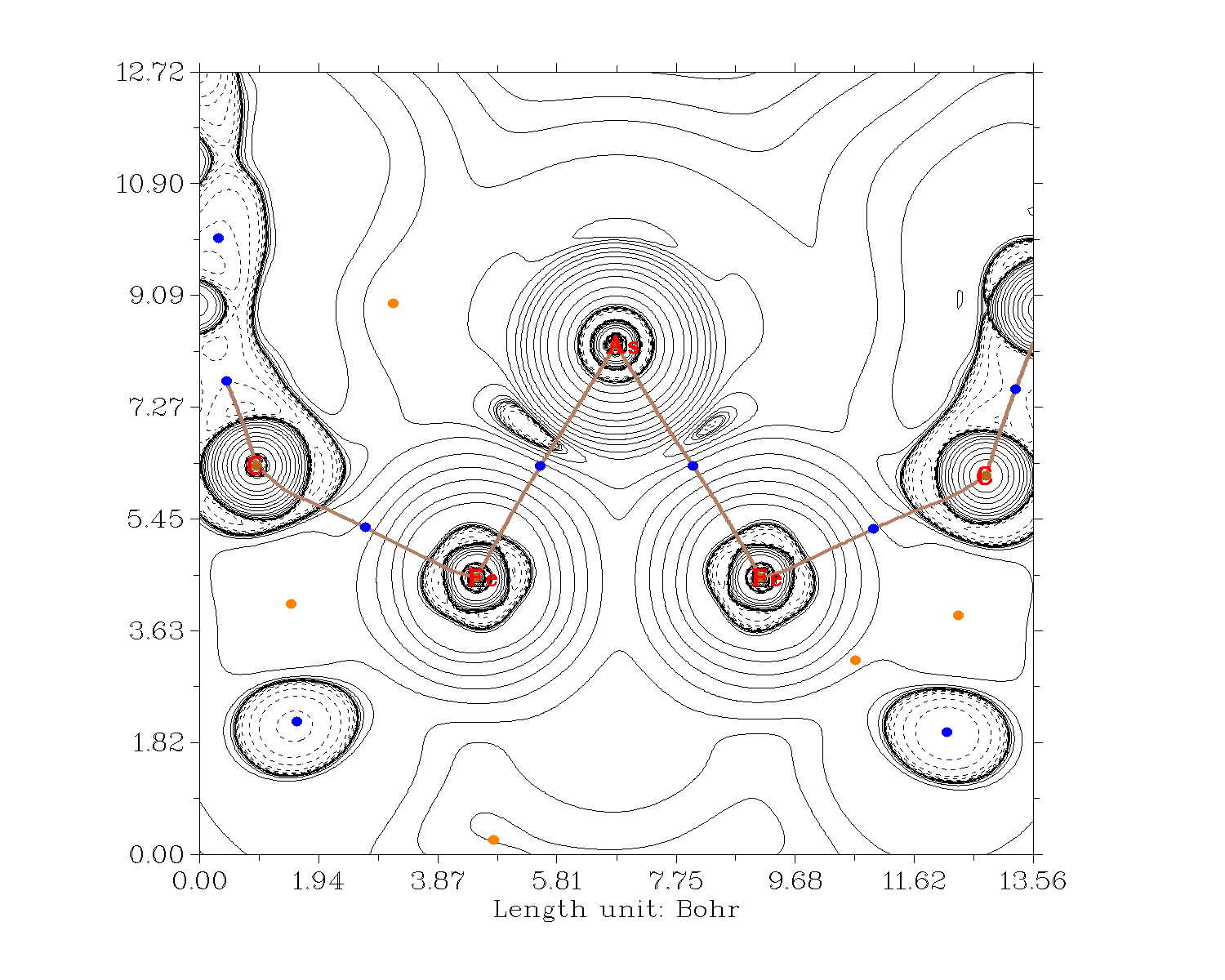
**

**Figure S43.** QTAIM for complex **3** on the Fe-As-Fe plane

**
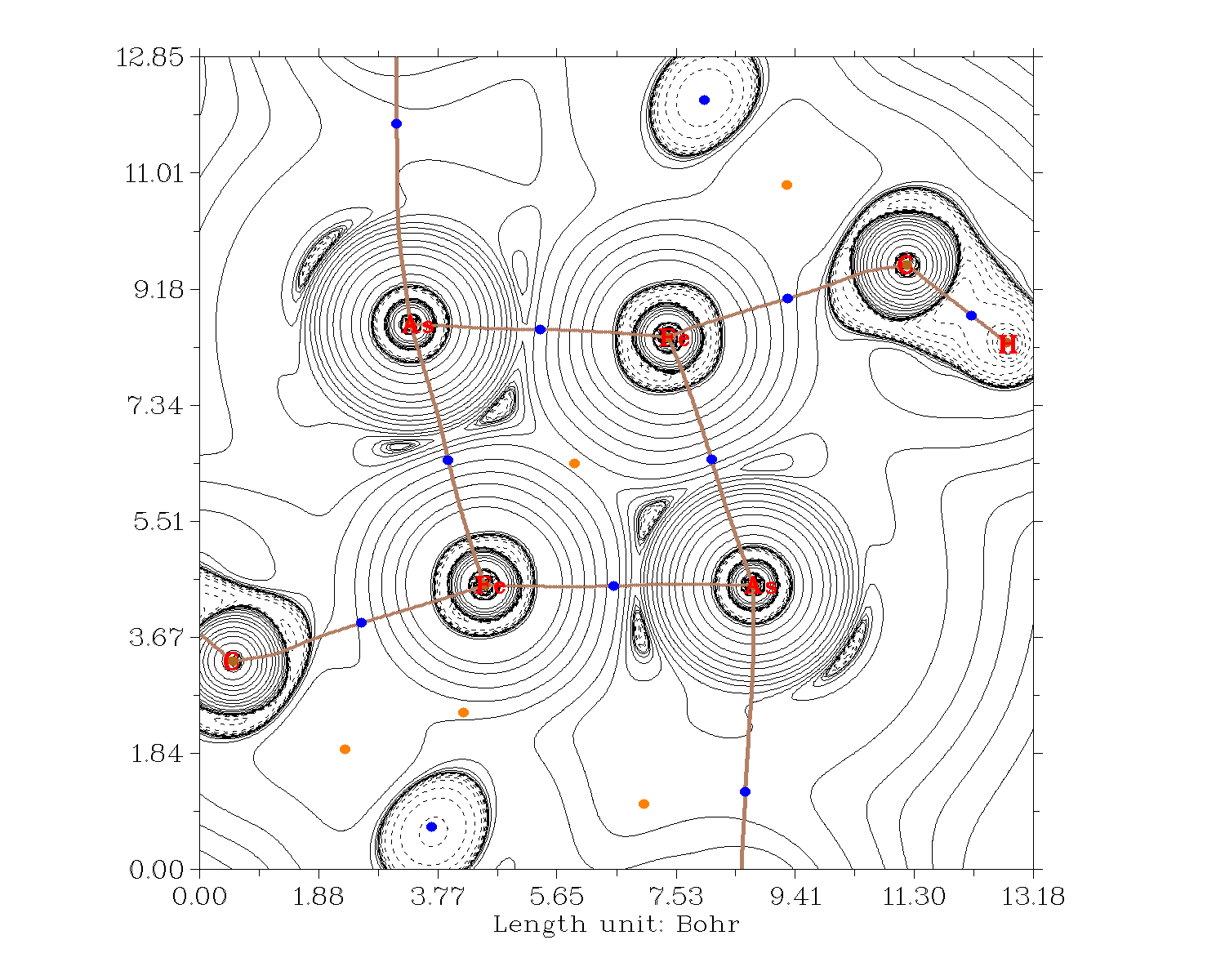
**

**Figure S44.** QTAIM for complex **4** on the Fe-As-Fe-As plane

**Table S4.** Comparison of the open-shell singlet and triplet spin states of [{Cp’Fe(μ-X)}_2_] (x = P, As or N) at the DFT level of theory.

| **[{Cp′Fe(μ-X)}_2_]** | **X = P** | | **X = As** | | **X = N** | |
| --- | --- | --- | --- | --- | --- | --- |
|  | open shell singlet | triplet | open shell singlet | triplet | open shell singlet | triplet |
| **Relative Stability in kcal mol^-1^** | | | | | | |
| Δ_r_G | 4.6 | 0.0 | 2.0 | 0.0 | 3.4 | 0.0 |
| Δ_r_H | 1.5 | 0.0 | 0.2 | 0.0 | 0.5 | 0.0 |
| **Distances in Å** | | | | | | |
| d(Fe - Fe) | 2.462 | 2.482 | 2.521 | 2.580 | 2.196 | 2.299 |
| d(X - X) | 3.361 | 3.386 | 3.572 | 3.601 | 2.629 | 2.600 |
| d(Fe - X) | 2.093 / 2.075  2.074 / 2.091 | 2.142 / 2.145  2.154 / 2.150 | 2.205 / 2.168  2.168 / 2.203 | 2.268 / 2.161  2.246 / 2.187 | 1.717 / 1.710  1.710 / 1.714 | 1.707 / 1.763  1.706 / 1.765 |
| **Wiberg Bond Indices** | | | | | | |
| Fe - Fe | 0.52 | 0.41 | 0.49 | 0.36 | 0.58 | 0.35 |
| X - X | 0.23 | 0.12 | 0.19 | 0.22 | 0.15 | 0.10 |
| Fe - X | 1.16 / 1.21  1.21 / 1.15 | 1.06 / 1.05  1.05 / 1.07 | 1.08 / 1.20  1.20 / 1.08 | 0.89 / 1.34  0.93 / 1.25 | 1.31 / 1.29  1.29 / 1.31 | 1.37 / 1.36  1.04 / 1.04 |
| **Natural Charges** | | | | | | |
| Fe | -0.92 / -0.91 | -0.56 /  -0.56 | -0.90 / -0.89 | -0.31 /  -1.05 | -0.12 / -0.12 | -0.06 / 0.50 |
| X | 0.63 / 0.64 | 0.46 / 0.47 | 0.63 / 0.64 | 0.59 / 0.58 | -0.10 / -0.10 | -0.26 /  -0.26 |
| **Spin Density** | | | | | | |
| Fe | -0.34 / 0.30 | 2.02 / 2.02 | 0.71 / -0.71 | 2.65 / -0.47 | 0.66 / -0.67 | -1.29 / 2.86 |
| X | -0.07 / 0.06 | -0.92 / 0.90 | -0.09 / 0.09 | 0.05 / 0.08 | 0.01 / -0.01 | 0.07 / 0.09 |

**6.2 Cartesian coordinates**

96

Cp2Fe2I2, 2S+1 = 7, dispersion

C -1.12216 12.76760 15.04142

C -0.26843 13.11882 13.93695

C -0.59270 14.48480 13.61028

H -0.16467 15.02866 12.78252

C -1.54430 15.02776 14.50719

C -1.91238 13.95802 15.34069

H -2.66857 14.00575 16.11040

C -5.28840 11.35325 8.97404

C -6.38379 11.22906 9.92496

C -6.05245 10.14376 10.79150

H -6.63666 9.83903 11.64558

C -4.78788 9.60987 10.46064

C -4.32479 10.35286 9.34249

H -3.39318 10.17303 8.82685

C -1.16070 11.57791 16.00334

C 0.03041 11.71291 16.97256

H 0.99086 11.66491 16.45959

H -0.00011 10.91090 17.71877

H -0.01842 12.67017 17.50159

C -1.13995 10.21076 15.31291

H -1.96970 10.12586 14.60450

H -1.25270 9.41958 16.06163

H -0.20808 10.02005 14.78139

C -2.43404 11.61321 16.86798

H -2.46053 12.48631 17.52593

H -2.45293 10.72705 17.50982

H -3.34022 11.60552 16.25794

C 0.75951 12.33630 13.10893

C 0.09445 11.22356 12.28090

H -0.46339 10.52117 12.89676

H 0.85438 10.65916 11.72797

H -0.59983 11.65524 11.55433

C 1.86991 11.74756 13.99196

H 2.32312 12.52190 14.61914

H 2.65605 11.31996 13.36033

H 1.50780 10.95096 14.64235

C 1.44528 13.27653 12.10275

H 0.73023 13.68911 11.38460

H 2.19023 12.71174 11.53366

H 1.96139 14.10271 12.60259

C -2.17035 16.40566 14.47741

C -3.69585 16.27593 14.33912

H -3.95821 15.76959 13.40348

H -4.16692 17.26521 14.33758

H -4.12554 15.69739 15.16241

C -1.63169 17.21705 13.29575

H -0.54659 17.34919 13.36170

H -2.08919 18.21138 13.28423

H -1.86425 16.72859 12.34384

C -1.83115 17.12714 15.79177

H -2.22027 16.57644 16.65403

H -2.26984 18.13102 15.80377

H -0.74779 17.22486 15.91508

C -5.10941 12.13658 7.67021

C -3.63764 12.08957 7.21950

H -3.31897 11.07587 6.96143

H -2.96685 12.47943 7.99103

H -3.51481 12.70401 6.32223

C -5.94159 11.44096 6.57584

H -5.79453 11.93974 5.61084

H -7.00831 11.44910 6.80468

H -5.63107 10.39653 6.47188

C -5.48320 13.62146 7.75068

H -5.25824 14.10528 6.79423

H -4.90191 14.12838 8.52730

H -6.54131 13.78494 7.95128

C -7.64422 12.06807 10.16048

C -8.53576 12.13421 8.91198

H -8.78706 11.12658 8.56564

H -8.06575 12.66641 8.08541

H -9.47039 12.65484 9.14778

C -8.49544 11.43810 11.27593

H -8.80195 10.41760 11.02549

H -9.40082 12.03593 11.42047

H -7.95953 11.41877 12.22992

C -7.27498 13.48260 10.64638

H -8.17944 14.08322 10.79863

H -6.63220 14.01509 9.94688

H -6.75059 13.42532 11.60735

C -4.03701 8.48936 11.14822

C -2.63738 8.98322 11.54486

H -2.71320 9.82003 12.24834

H -2.06841 9.32537 10.67511

H -2.06420 8.18606 12.03144

C -4.77610 8.03343 12.40902

H -4.88112 8.85718 13.12218

H -4.22051 7.22814 12.90058

H -5.77464 7.65350 12.16971

C -3.90766 7.30694 10.17447

H -3.36542 6.47779 10.64302

H -3.36565 7.59987 9.26982

H -4.89520 6.94429 9.87227

Fe -4.42454 11.83313 10.98493

Fe -2.34898 13.29480 13.38349

I -2.62723 13.84595 10.80472

I -4.63645 12.05087 13.68980

96

Cp2Fe2I2, 2S+1 = 9, dispersion

C -1.12558 13.05102 15.26048

C -0.03917 13.22122 14.30600

C -0.39262 14.32872 13.47672

H 0.18203 14.67014 12.63008

C -1.66264 14.83244 13.83200

C -2.10844 14.04543 14.92712

H -3.03912 14.19500 15.45407

C -5.43504 11.51232 9.06360

C -6.26151 11.22556 10.22826

C -6.00565 9.86740 10.58643

H -6.42069 9.37249 11.45026

C -5.03182 9.30091 9.73474

C -4.69265 10.31161 8.79574

H -3.98482 10.19047 7.98922

C -1.27760 12.23527 16.54781

C -0.45426 12.93155 17.64865

H 0.61066 12.95273 17.41167

H -0.58205 12.41199 18.60533

H -0.78865 13.96625 17.77383

C -0.86604 10.76223 16.43921

H -1.44361 10.25052 15.66335

H -1.06637 10.25932 17.39121

H 0.19334 10.62952 16.22373

C -2.74713 12.23707 17.00800

H -3.08838 13.23658 17.29133

H -2.84953 11.59972 17.89168

H -3.41324 11.84820 16.23191

C 1.23093 12.40810 14.03587

C 0.87458 10.99992 13.52261

H 0.24554 10.44388 14.21613

H 1.78454 10.41422 13.34735

H 0.34034 11.07181 12.56767

C 2.13913 12.32924 15.27161

H 2.38276 13.33346 15.63297

H 3.07686 11.82474 15.01421

H 1.68657 11.77681 16.09457

C 2.05765 13.07570 12.92395

H 1.50784 13.11103 11.97835

H 2.96826 12.49321 12.75244

H 2.35545 14.09355 13.19477

C -2.43028 15.96881 13.19050

C -3.81279 15.46675 12.74652

H -3.70895 14.68263 11.98847

H -4.40198 16.28013 12.30852

H -4.38016 15.05326 13.58595

C -1.68265 16.50721 11.96788

H -0.69683 16.89564 12.24349

H -2.24917 17.32475 11.51038

H -1.54790 15.72564 11.21351

C -2.60062 17.09535 14.22268

H -3.15310 16.74555 15.10035

H -3.15093 17.93719 13.78759

H -1.62619 17.46058 14.56198

C -5.38348 12.67827 8.07192

C -4.13095 12.56769 7.18334

H -4.15400 11.67711 6.54934

H -3.21363 12.54942 7.77883

H -4.08356 13.43557 6.51838

C -6.60583 12.57644 7.13968

H -6.57170 13.36693 6.38109

H -7.54730 12.66597 7.68278

H -6.60833 11.61005 6.62571

C -5.32446 14.06630 8.72120

H -5.21058 14.82861 7.94314

H -4.46362 14.14395 9.39333

H -6.22550 14.31269 9.28172

C -7.19597 12.09820 11.07346

C -8.30915 12.73747 10.23082

H -8.85824 11.97277 9.67227

H -7.92995 13.46962 9.51811

H -9.01937 13.25533 10.88467

C -7.88961 11.24156 12.14626

H -8.47856 10.43293 11.70213

H -8.56903 11.87299 12.72739

H -7.16704 10.80819 12.84445

C -6.40257 13.17846 11.83027

H -7.07944 13.81541 12.41162

H -5.82898 13.82094 11.16492

H -5.70492 12.71041 12.53320

C -4.42374 7.91582 9.80162

C -2.89256 8.03386 9.88126

H -2.59141 8.57258 10.78839

H -2.48609 8.57049 9.01837

H -2.42762 7.04211 9.91120

C -4.92854 7.16237 11.03499

H -4.66508 7.69241 11.95599

H -4.47769 6.16572 11.07821

H -6.01587 7.03732 11.00819

C -4.81272 7.13939 8.53325

H -4.38557 6.13043 8.55150

H -4.44842 7.64643 7.63429

H -5.90062 7.05035 8.45187

Fe -4.00724 11.09440 10.74014

Fe -1.98429 12.61482 13.24488

I -1.73868 12.56183 10.49846

I -3.67105 10.49285 13.40996

4

KAsCO, dispersion

C 0.13813 0.40106 0.64429

O 0.27921 -0.46074 -0.18403

As 0.05610 1.56251 1.93696

K 2.07717 -1.02846 2.05485

2

KI, dispersion

K 1.06674 -0.00484 1.66090

I 3.02926 -0.60305 4.13348

4

KPCO, dispersion

C 0.14523 0.43348 0.69582

O 0.33320 -0.46273 -0.09866

P 0.02132 1.54963 1.86434

K 2.05086 -1.04602 1.99058

100

Cp2Fe2As2CO2, 2S+1=7, dispersion

As 5.75975 5.05725 11.03656

Fe 6.69092 6.90186 9.54060

C 5.44068 7.73348 7.84662

C 6.76365 8.30780 7.66020

C 7.02136 9.12399 8.80288

H 7.94977 9.63965 8.99239

C 5.94070 9.06922 9.70987

C 4.97014 8.22401 9.10968

H 4.00983 7.98735 9.54254

C 4.47693 6.97065 6.93362

C 3.36160 6.31737 7.77161

H 3.76877 5.68104 8.56156

H 2.73517 5.69644 7.12404

H 2.70820 7.06071 8.23609

C 3.79902 7.98495 5.99205

H 3.29093 8.76011 6.57413

H 3.05307 7.48326 5.36474

H 4.51772 8.47940 5.33653

C 5.11574 5.84592 6.11243

H 5.83851 6.21222 5.38412

H 4.33713 5.31557 5.55401

H 5.61086 5.11722 6.76035

C 7.80091 8.17030 6.54096

C 7.22285 8.62067 5.19019

H 8.01306 8.63554 4.43136

H 6.80631 9.62992 5.26756

H 6.43597 7.95801 4.82973

C 8.35661 6.74019 6.43620

H 8.91010 6.48642 7.34416

H 9.05244 6.66324 5.59294

H 7.58071 5.98970 6.30410

C 9.00976 9.07669 6.82952

H 8.72211 10.13072 6.89121

H 9.73981 8.97384 6.02038

H 9.50849 8.79558 7.76255

C 5.77926 9.82454 11.01118

C 7.12282 10.38808 11.48251

H 7.85574 9.59051 11.63253

H 6.99814 10.91463 12.43418

H 7.53243 11.10050 10.75933

C 5.21285 8.90057 12.09809

H 4.25928 8.45820 11.79411

H 5.04310 9.45903 13.02511

H 5.90957 8.08726 12.32025

C 4.79875 10.98619 10.77157

H 5.17448 11.65548 9.99113

H 4.65863 11.57038 11.68826

H 3.82117 10.61169 10.45188

As 9.15102 6.48949 10.12315

Fe 8.15187 4.39456 11.03821

C 9.58711 3.34405 12.22742

C 8.30388 2.69768 12.31789

C 7.98197 2.24438 10.97441

H 7.06844 1.73520 10.70756

C 9.02055 2.54004 10.07522

C 9.95533 3.28826 10.82758

H 10.86133 3.71442 10.42211

C 10.62917 3.81383 13.24647

C 11.60470 4.81301 12.59618

H 11.07988 5.66263 12.15187

H 12.29111 5.19496 13.35804

H 12.21768 4.34808 11.81922

C 11.45345 2.58074 13.66696

H 11.90777 2.10742 12.79056

H 12.25810 2.87754 14.34934

H 10.83937 1.83111 14.16947

C 10.06553 4.50637 14.48976

H 9.45048 3.84532 15.09960

H 10.89490 4.84478 15.11979

H 9.47283 5.38362 14.21760

C 7.39028 2.35867 13.50132

C 8.13376 1.44626 14.49131

H 7.43996 1.08266 15.25714

H 8.55662 0.57813 13.97534

H 8.94610 1.96298 15.00344

C 6.84332 3.60250 14.22296

H 6.10674 4.10653 13.59253

H 6.34047 3.30458 15.14996

H 7.61349 4.32814 14.47127

C 6.16004 1.57625 13.01092

H 6.43502 0.61977 12.55516

H 5.50712 1.36364 13.86322

H 5.58005 2.15996 12.28890

C 9.11803 2.21835 8.59894

C 8.03755 1.21116 8.19475

H 7.03455 1.62110 8.32981

H 8.14931 0.94748 7.13809

H 8.11962 0.29104 8.78294

C 8.96126 3.50168 7.76989

H 9.74089 4.22725 8.02200

H 9.03461 3.28490 6.69838

H 7.99065 3.96967 7.95656

C 10.50019 1.60494 8.32097

H 10.65150 0.69740 8.91394

H 10.59271 1.34165 7.26178

H 11.30536 2.30544 8.56301

C 8.53626 6.86053 11.75916

C 5.62427 4.05598 9.56623

O 8.18272 7.11789 12.84947

O 5.45618 3.36381 8.63815

100

Cp2Fe2As2CO2, 2S+1=9, dispersion

As 5.76825 4.66918 10.31888

Fe 6.68331 6.85755 9.32539

C 5.31339 7.83855 7.79670

C 6.62008 8.46051 7.65130

C 6.89241 9.14297 8.87810

H 7.81378 9.65686 9.10441

C 5.83858 8.94665 9.79703

C 4.87627 8.14732 9.12571

H 3.93697 7.83080 9.55420

C 4.30465 7.23377 6.81355

C 3.26979 6.38234 7.57380

H 3.75114 5.61028 8.18125

H 2.60672 5.88878 6.85663

H 2.63778 6.98639 8.23005

C 3.55122 8.40882 6.15852

H 3.07799 9.03282 6.92294

H 2.76952 8.03457 5.48736

H 4.22302 9.04540 5.57823

C 4.87754 6.33027 5.71745

H 5.55802 6.85242 5.04610

H 4.05326 5.95094 5.10402

H 5.39704 5.46822 6.14092

C 7.62377 8.47450 6.49603

C 7.02267 9.16967 5.26350

H 7.77798 9.25660 4.47456

H 6.68432 10.17785 5.52326

H 6.17216 8.62924 4.84810

C 8.11544 7.05938 6.14344

H 8.68493 6.64125 6.97930

H 8.78427 7.09541 5.27610

H 7.30727 6.36775 5.92005

C 8.87491 9.27702 6.89068

H 8.63575 10.32172 7.11198

H 9.58783 9.26585 6.06015

H 9.37276 8.83961 7.76207

C 5.68592 9.50945 11.19334

C 6.96606 10.22056 11.63837

H 7.81997 9.53924 11.64788

H 6.84437 10.61688 12.65148

H 7.20058 11.06035 10.97628

C 5.35418 8.37866 12.17934

H 4.44314 7.84841 11.88448

H 5.20035 8.77768 13.18794

H 6.17046 7.65191 12.23009

C 4.52827 10.52309 11.17912

H 4.73100 11.33253 10.47073

H 4.38877 10.96284 12.17312

H 3.58955 10.04434 10.88355

As 9.24764 6.45081 9.71537

Fe 8.33297 4.26115 10.70609

C 9.70123 3.27642 12.23275

C 8.39514 2.65225 12.37439

C 8.12539 1.97336 11.14504

H 7.20511 1.45875 10.91592

C 9.18036 2.17370 10.22831

C 10.14066 2.97220 10.90343

H 11.08013 3.29133 10.47724

C 10.70786 3.87957 13.21906

C 11.74246 4.73488 12.46285

H 11.26085 5.50813 11.85715

H 12.40384 5.22716 13.18246

H 12.37627 4.13386 11.80555

C 11.46231 2.70371 13.87147

H 11.93763 2.08292 13.10574

H 12.24247 3.07715 14.54488

H 10.79080 2.06419 14.44884

C 10.13200 4.77883 14.31711

H 9.45187 4.25336 14.98623

H 10.95481 5.15815 14.93250

H 9.61121 5.64100 13.89539

C 7.39051 2.63212 13.52880

C 7.99204 1.93306 14.75890

H 7.23618 1.84098 15.54672

H 8.33307 0.92681 14.49517

H 8.84082 2.47382 15.17741

C 6.89598 4.04479 13.88711

H 6.32533 4.46499 13.05313

H 6.22753 4.00383 14.75453

H 7.70275 4.73722 14.11304

C 6.14107 1.82906 13.12990

H 6.38213 0.78558 12.90487

H 5.42735 1.83590 13.95979

H 5.64337 2.26901 12.25970

C 9.33617 1.61520 8.83060

C 8.05889 0.90089 8.38246

H 7.20272 1.57934 8.37349

H 8.18308 0.50733 7.36857

H 7.82650 0.05877 9.04234

C 9.66516 2.74970 7.84793

H 10.57405 3.28226 8.14528

H 9.82154 2.35384 6.83847

H 8.84644 3.47376 7.79812

C 10.49716 0.60535 8.84323

H 10.29648 -0.20661 9.54930

H 10.63894 0.16869 7.84819

H 11.43408 1.08636 9.14085

C 8.83323 6.92381 11.38153

C 6.18253 4.19370 8.65341

O 8.61508 7.27442 12.47925

O 6.40030 3.84117 7.55620

100

Cp2Fe2P2CO2, 2S+1=7, dispersion

P 5.82759 5.07045 10.99740

Fe 6.71942 6.88004 9.56472

C 5.49084 7.71442 7.85559

C 6.80755 8.31046 7.68844

C 7.03954 9.12139 8.84034

H 7.95691 9.65147 9.04356

C 5.94752 9.04738 9.73266

C 4.99653 8.19164 9.11536

H 4.03278 7.94182 9.53311

C 4.55737 6.93223 6.92750

C 3.42252 6.28380 7.74218

H 3.80990 5.65131 8.54461

H 2.81430 5.65719 7.08287

H 2.75652 7.02969 8.18418

C 3.89504 7.92266 5.95026

H 3.36283 8.70105 6.50596

H 3.17105 7.40154 5.31323

H 4.62337 8.41484 5.30474

C 5.23296 5.79728 6.15042

H 5.98216 6.15426 5.44469

H 4.48008 5.25035 5.57305

H 5.70735 5.08454 6.83165

C 7.86001 8.19785 6.57998

C 7.28307 8.60511 5.21514

H 8.08537 8.64930 4.47037

H 6.81748 9.59406 5.27209

H 6.53692 7.90042 4.84821

C 8.46879 6.78756 6.50278

H 9.04404 6.57873 7.40880

H 9.15508 6.71533 5.65116

H 7.71818 6.00675 6.39887

C 9.03016 9.15448 6.86611

H 8.69962 10.19655 6.91668

H 9.76764 9.07353 6.06120

H 9.53620 8.90191 7.80299

C 5.75626 9.79405 11.03502

C 7.07724 10.40592 11.50945

H 7.83708 9.63502 11.66459

H 6.93047 10.92877 12.46000

H 7.46313 11.13173 10.78643

C 5.22259 8.84732 12.11920

H 4.28987 8.36594 11.81001

H 5.02509 9.39848 13.04516

H 5.95102 8.06337 12.34523

C 4.73419 10.91934 10.79557

H 5.08577 11.60283 10.01615

H 4.57254 11.49696 11.71284

H 3.77081 10.51028 10.47516

P 9.07810 6.43408 10.17286

Fe 8.11972 4.39267 11.01545

C 9.56753 3.34655 12.19571

C 8.28468 2.70456 12.30949

C 7.93953 2.24962 10.97384

H 7.01940 1.74469 10.72195

C 8.96708 2.53432 10.05627

C 9.91652 3.28004 10.78984

H 10.81561 3.70526 10.36849

C 10.62523 3.81570 13.19870

C 11.60215 4.80098 12.53018

H 11.07997 5.65863 12.09867

H 12.30740 5.17292 13.27966

H 12.19439 4.32912 11.74158

C 11.44229 2.57769 13.61898

H 11.88399 2.09474 12.74137

H 12.25600 2.87137 14.29185

H 10.82596 1.83703 14.13188

C 10.08259 4.52435 14.44244

H 9.46872 3.87490 15.06561

H 10.92273 4.86021 15.05945

H 9.49555 5.40526 14.16966

C 7.37967 2.38851 13.50538

C 8.12352 1.48885 14.50651

H 7.43318 1.14792 15.28573

H 8.53233 0.60597 14.00439

H 8.94593 2.00665 15.00096

C 6.84562 3.64967 14.20581

H 6.12968 4.16315 13.55913

H 6.32403 3.37041 15.12832

H 7.62696 4.36140 14.46011

C 6.14234 1.60379 13.03703

H 6.41046 0.64053 12.59145

H 5.49723 1.40425 13.89832

H 5.55591 2.17781 12.31280

C 9.02512 2.22210 8.57577

C 7.93826 1.21248 8.19518

H 6.93772 1.61876 8.35840

H 8.02357 0.95472 7.13460

H 8.03874 0.28958 8.77613

C 8.83280 3.51256 7.76518

H 9.61246 4.24315 8.00041

H 8.87232 3.30807 6.68946

H 7.86398 3.96934 7.98848

C 10.40226 1.62162 8.25114

H 10.57672 0.70871 8.82934

H 10.46548 1.37039 7.18692

H 11.20976 2.32481 8.47706

C 8.58502 6.88519 11.70151

C 5.59371 4.07016 9.68680

O 8.30469 7.21053 12.79686

O 5.34352 3.33797 8.80468

100

Cp2Fe2P2CO2, 2S+1=9, dispersion

P 5.71676 5.08330 10.91759

Fe 6.64185 6.95878 9.54786

C 5.43452 7.76474 7.83099

C 6.76141 8.34328 7.67182

C 6.98325 9.17992 8.80751

H 7.90401 9.70232 9.01460

C 5.87971 9.13099 9.68628

C 4.92907 8.27046 9.07553

H 3.95878 8.03557 9.48693

C 4.49902 6.99032 6.89819

C 3.35318 6.35422 7.70721

H 3.72945 5.71959 8.51361

H 2.74264 5.73282 7.04523

H 2.69146 7.10706 8.14389

C 3.85709 7.99241 5.91908

H 3.33135 8.77711 6.47201

H 3.13139 7.48267 5.27494

H 4.59827 8.47543 5.28068

C 5.16241 5.85005 6.11861

H 5.91639 6.20007 5.41456

H 4.40264 5.31666 5.53779

H 5.62542 5.12645 6.79539

C 7.82998 8.18977 6.58452

C 7.28855 8.60157 5.20654

H 8.10230 8.60736 4.47305

H 6.86026 9.60807 5.24666

H 6.52059 7.92254 4.83649

C 8.39695 6.76062 6.53525

H 8.94678 6.54277 7.45516

H 9.09996 6.65798 5.70076

H 7.62624 6.00115 6.42171

C 9.02220 9.11453 6.88323

H 8.72317 10.16699 6.90874

H 9.77496 8.99778 6.09720

H 9.49837 8.86288 7.83593

C 5.69148 9.87344 10.99132

C 7.00463 10.51794 11.44310

H 7.78720 9.76690 11.58335

H 6.86199 11.03591 12.39678

H 7.35923 11.25309 10.71354

C 5.20414 8.90653 12.08044

H 4.27635 8.40555 11.78808

H 5.01622 9.44428 13.01611

H 5.95598 8.13787 12.28426

C 4.63515 10.97085 10.77527

H 4.95382 11.66704 9.99303

H 4.47486 11.53992 11.69796

H 3.67718 10.53771 10.47085

P 9.04068 6.63938 10.22101

Fe 8.15705 4.42262 10.98540

C 9.64845 3.24631 12.20777

C 8.36913 2.56018 12.33348

C 8.03216 2.07743 11.02867

H 7.11870 1.55971 10.77949

C 9.02351 2.44579 10.09257

C 10.00711 3.16062 10.82250

H 10.90274 3.58343 10.39242

C 10.68121 3.76343 13.21466

C 11.65826 4.73226 12.52243

H 11.13062 5.56247 12.04396

H 12.34461 5.14791 13.26665

H 12.27007 4.23368 11.76600

C 11.50561 2.55899 13.70963

H 11.97569 2.04789 12.86364

H 12.29664 2.89292 14.39104

H 10.88756 1.82998 14.23670

C 10.10559 4.52396 14.41318

H 9.48320 3.89980 15.05327

H 10.92836 4.89761 15.03199

H 9.51659 5.38662 14.08895

C 7.46026 2.28822 13.53667

C 8.20312 1.47053 14.60564

H 7.50774 1.17166 15.39776

H 8.63083 0.56296 14.16768

H 9.01170 2.03154 15.07449

C 6.88636 3.58017 14.14578

H 6.18791 4.04667 13.44534

H 6.33220 3.34915 15.06274

H 7.65039 4.31467 14.39056

C 6.24738 1.44940 13.10003

H 6.54702 0.47706 12.69685

H 5.60308 1.26943 13.96636

H 5.64946 1.97307 12.34744

C 9.05924 2.17379 8.60232

C 7.95122 1.19675 8.20057

H 6.96025 1.60746 8.40599

H 8.00799 0.98633 7.12757

H 8.05312 0.24781 8.73710

C 8.87766 3.49690 7.84061

H 9.66720 4.20976 8.09916

H 8.90682 3.33810 6.75676

H 7.91207 3.95403 8.08278

C 10.42141 1.56446 8.23508

H 10.58869 0.63044 8.78046

H 10.46579 1.34824 7.16199

H 11.24319 2.24631 8.47396

C 8.59895 7.04736 11.77310

C 5.56478 4.11026 9.57996

O 8.32908 7.36146 12.87277

O 5.37380 3.39296 8.67091

98

Cp2Fe2As2CO, 2S+1=3, dispersion - adduct CO decoord.

C 7.21269 2.62040 8.85256

C 7.10986 4.04268 9.12170

C 6.46030 4.62240 7.96375

H 6.22826 5.66968 7.85422

C 6.15172 3.62325 7.01438

C 6.64774 2.41306 7.55603

H 6.63104 1.46395 7.04372

C 7.76418 1.44706 9.66538

C 7.02359 1.31655 11.00616

H 7.20078 2.15660 11.67663

H 7.35416 0.40847 11.52158

H 5.94384 1.23718 10.84258

C 9.28170 1.56654 9.87161

H 9.78447 1.47290 8.90613

H 9.64091 0.76091 10.52186

H 9.57898 2.51932 10.30606

C 7.53248 0.12602 8.91061

H 6.46674 -0.06842 8.75047

H 7.93365 -0.69895 9.50777

H 8.04377 0.11797 7.94482

C 7.25487 4.89525 10.38541

C 7.39231 6.38467 10.01834

H 8.24018 6.55140 9.34850

H 7.55605 6.96515 10.93140

H 6.49080 6.78211 9.54450

C 8.44975 4.56172 11.28241

H 8.42811 3.54523 11.67136

H 8.45148 5.23961 12.14221

H 9.38840 4.70570 10.73992

C 5.94276 4.73279 11.18042

H 5.08432 5.02504 10.56702

H 5.96009 5.37383 12.06870

H 5.78580 3.70263 11.50585

C 5.41176 3.77792 5.70589

C 6.13119 3.00211 4.59822

H 7.14037 3.39545 4.46077

H 5.58893 3.09412 3.65143

H 6.21893 1.93798 4.83525

C 3.99651 3.20480 5.90258

H 4.03688 2.14515 6.17400

H 3.41524 3.29882 4.97879

H 3.46537 3.73819 6.69749

C 5.31165 5.25161 5.30394

H 4.75329 5.83407 6.04397

H 4.78901 5.34387 4.34668

H 6.30541 5.69770 5.19586

C 12.35643 4.55671 5.71540

C 12.30340 3.11307 5.84748

C 11.42330 2.65662 4.81171

H 11.14144 1.62706 4.66027

C 10.93169 3.74061 4.03319

C 11.50178 4.89807 4.60629

H 11.32664 5.90409 4.25841

C 13.27540 5.64768 6.27579

C 13.74908 5.47245 7.71946

H 12.89913 5.44089 8.40690

H 14.37048 6.33046 7.99610

H 14.35177 4.57729 7.86537

C 12.59651 7.02823 6.21259

H 12.36461 7.33882 5.19101

H 13.27400 7.78010 6.62909

H 11.67186 7.04202 6.79526

C 14.50300 5.68243 5.34106

H 15.04449 4.73351 5.35142

H 15.19238 6.47457 5.65355

H 14.19767 5.88253 4.30919

C 13.02468 2.12242 6.76266

C 12.71102 2.34112 8.24864

H 12.98585 3.33011 8.60464

H 13.24610 1.60046 8.85388

H 11.64019 2.21978 8.42007

C 12.57047 0.68792 6.43957

H 11.49252 0.56530 6.57535

H 13.07599 -0.00735 7.11708

H 12.82907 0.39744 5.41630

C 14.53976 2.17746 6.50408

H 14.75496 2.00970 5.44358

H 15.04671 1.39593 7.08055

H 14.98030 3.13414 6.78718

C 10.10801 3.67732 2.76844

C 9.10376 4.83382 2.71764

H 8.42309 4.79660 3.57122

H 8.51437 4.78535 1.79591

H 9.60801 5.80497 2.73996

C 11.07983 3.80190 1.58022

H 11.61516 4.75639 1.61067

H 10.53535 3.74485 0.63100

H 11.82197 2.99722 1.59788

C 9.36576 2.34152 2.66844

H 10.06512 1.50258 2.59516

H 8.73761 2.32750 1.77189

H 8.72970 2.17072 3.53866

As 9.00303 5.78990 6.34085

As 10.21187 4.96281 8.13730

Fe 8.30918 3.79058 7.37900

Fe 10.36528 3.91314 6.05794

O 9.05292 1.33530 6.03121

C 9.23715 2.47808 6.30241

98

Cp2Fe2As2CO, 2S+1=3, dispersion - TS CO decoord.

As 8.95170 5.77949 6.32249

As 10.17920 5.08361 8.19368

Fe 8.30784 3.87253 7.46521

Fe 10.41119 3.99685 6.12343

O 9.00992 1.46406 6.01640

C 7.22129 2.70640 8.90668

C 7.09271 4.12001 9.18750

C 6.47725 4.70508 8.01863

H 6.24633 5.75304 7.91042

C 6.20148 3.71648 7.04292

C 6.68855 2.50552 7.59372

H 6.68691 1.55907 7.07640

C 7.77261 1.53204 9.71827

C 7.02458 1.39061 11.05359

H 7.19622 2.22694 11.73013

H 7.35369 0.48005 11.56562

H 5.94598 1.31044 10.88304

C 9.28824 1.65727 9.93581

H 9.79883 1.56910 8.97383

H 9.64597 0.85126 10.58665

H 9.57866 2.61084 10.37272

C 7.55208 0.21305 8.95636

H 6.48858 0.01723 8.78391

H 7.94935 -0.61361 9.55387

H 8.07191 0.20882 7.99500

C 7.23001 4.96811 10.45430

C 7.35342 6.46042 10.09368

H 8.20030 6.63778 9.42506

H 7.51269 7.03889 11.00880

H 6.44795 6.85113 9.62170

C 8.42975 4.64229 11.34787

H 8.41743 3.62430 11.73338

H 8.42826 5.31722 12.21014

H 9.36543 4.79537 10.80280

C 5.92045 4.79234 11.25028

H 5.05906 5.07843 10.63810

H 5.93260 5.43103 12.14042

H 5.77262 3.75998 11.57288

C 5.44341 3.86859 5.74543

C 6.15939 3.11164 4.62392

H 7.15823 3.52597 4.47542

H 5.60170 3.19535 3.68520

H 6.27245 2.04889 4.85664

C 4.03929 3.27259 5.95513

H 4.09960 2.21150 6.21707

H 3.44320 3.36537 5.04058

H 3.51142 3.79079 6.76225

C 5.31209 5.34253 5.35443

H 4.75490 5.91054 6.10648

H 4.77415 5.43225 4.40530

H 6.29603 5.80661 5.23466

C 12.41575 4.60267 5.71026

C 12.37109 3.16049 5.81364

C 11.46275 2.71260 4.80365

H 11.16929 1.68562 4.65698

C 10.95075 3.80182 4.05050

C 11.52502 4.95774 4.63036

H 11.35088 5.96628 4.28970

C 13.33845 5.68721 6.27425

C 13.79333 5.50914 7.72398

H 12.93218 5.48677 8.39747

H 14.41927 6.36170 8.00716

H 14.38653 4.60906 7.87941

C 12.66100 7.06850 6.20452

H 12.46221 7.38820 5.17846

H 13.32536 7.81621 6.64845

H 11.71906 7.07347 6.75909

C 14.57370 5.71891 5.35024

H 15.11826 4.77188 5.37029

H 15.25814 6.51483 5.66389

H 14.27668 5.91271 4.31460

C 13.07745 2.16117 6.73164

C 12.73115 2.37212 8.21268

H 12.98827 3.36348 8.57544

H 13.25972 1.63403 8.82656

H 11.65794 2.23997 8.36475

C 12.63134 0.72885 6.38940

H 11.55300 0.59981 6.51890

H 13.13535 0.02819 7.06227

H 12.89649 0.45142 5.36419

C 14.59704 2.22139 6.50460

H 14.83490 2.05679 5.44854

H 15.09273 1.43925 7.08972

H 15.02890 3.17807 6.79971

C 10.12666 3.74246 2.78551

C 9.14130 4.91352 2.71712

H 8.44340 4.88795 3.55712

H 8.56695 4.87159 1.78576

H 9.65889 5.87737 2.74606

C 11.10801 3.84452 1.60183

H 11.65567 4.79208 1.62739

H 10.56774 3.78645 0.65032

H 11.83901 3.03010 1.63066

C 9.36578 2.41699 2.68926

H 10.05287 1.56740 2.62370

H 8.74297 2.40782 1.78899

H 8.72249 2.26026 3.55636

C 9.15834 2.59304 6.35620

98

Cp2Fe2As2CO, 2S+1=3, dispersion - product CO decoord.

C 7.10870 2.58887 8.25998

C 6.98872 3.82303 9.03121

C 6.31656 4.75743 8.18893

H 6.12635 5.78693 8.44895

C 6.01869 4.18721 6.93372

C 6.51136 2.85222 6.98299

H 6.40129 2.12838 6.19004

C 7.68950 1.20695 8.58198

C 7.10220 0.61529 9.87092

H 7.39134 1.17260 10.76097

H 7.45508 -0.41373 9.99998

H 6.00896 0.59498 9.82278

C 9.22297 1.26407 8.65906

H 9.62953 1.60762 7.70040

H 9.63674 0.27202 8.87456

H 9.57982 1.95274 9.42342

C 7.34445 0.21785 7.45563

H 6.26284 0.11456 7.32527

H 7.74715 -0.76839 7.70593

H 7.78272 0.52144 6.50062

C 7.25862 4.17481 10.49613

C 7.29060 5.70304 10.67998

H 8.04054 6.16460 10.03068

H 7.54647 5.93616 11.71831

H 6.31949 6.16262 10.47682

C 8.58846 3.64783 11.04580

H 8.63892 2.56001 11.07552

H 8.72750 4.00548 12.07138

H 9.42441 4.01875 10.44407

C 6.08453 3.64424 11.34398

H 5.14163 4.07524 10.99305

H 6.21906 3.92754 12.39409

H 5.99277 2.55861 11.29395

C 5.19427 4.79020 5.81780

C 5.79740 4.45320 4.44917

H 6.79608 4.88919 4.35377

H 5.16896 4.85280 3.64612

H 5.88475 3.37302 4.29830

C 3.78058 4.18396 5.90928

H 3.81134 3.09682 5.78454

H 3.13302 4.60010 5.12935

H 3.32837 4.39951 6.88248

C 5.10067 6.31118 5.96614

H 4.60375 6.59230 6.90009

H 4.51920 6.73436 5.14064

H 6.09614 6.76606 5.95437

C 12.56924 4.51494 5.53378

C 12.41581 3.26222 6.25338

C 11.39965 2.52052 5.58065

H 11.05067 1.54260 5.87208

C 10.93056 3.23211 4.44494

C 11.67031 4.44747 4.41587

H 11.54150 5.22840 3.68326

C 13.48783 5.72889 5.69275

C 13.73016 6.15340 7.14503

H 12.77783 6.35854 7.64371

H 14.32793 7.07077 7.16044

H 14.26838 5.40891 7.72939

C 12.85898 6.95483 5.00270

H 12.78522 6.83155 3.91893

H 13.48724 7.83253 5.18365

H 11.85979 7.15815 5.39951

C 14.82434 5.43682 4.98456

H 15.35247 4.59229 5.43029

H 15.47969 6.31360 5.03503

H 14.65089 5.20206 3.92942

C 13.14008 2.67010 7.46725

C 12.70834 3.31913 8.79220

H 12.88025 4.39387 8.80871

H 13.25823 2.86773 9.62627

H 11.63916 3.15841 8.95232

C 12.81669 1.17016 7.58740

H 11.75968 0.99948 7.79947

H 13.38792 0.74294 8.41752

H 13.08486 0.62555 6.67646

C 14.66439 2.75677 7.28808

H 14.96835 2.29582 6.34275

H 15.16197 2.21922 8.10229

H 15.03560 3.78008 7.29845

C 9.95556 2.72233 3.40891

C 9.37051 3.88156 2.59775

H 8.87793 4.60279 3.25642

H 8.63454 3.50793 1.87842

H 10.14642 4.40931 2.03411

C 10.72080 1.77231 2.46969

H 11.55281 2.29278 1.98478

H 10.05802 1.38259 1.68884

H 11.13115 0.92410 3.02684

C 8.81538 1.95479 4.08375

H 9.19092 1.10000 4.65314

H 8.10965 1.57762 3.33543

H 8.27239 2.61291 4.76977

As 8.92286 6.04974 5.97616

As 9.94876 5.58240 8.04311

Fe 8.32558 4.04170 7.15024

Fe 10.54384 4.42892 6.14329

O 10.45304 -1.92419 6.75573

C 10.40915 -0.97202 6.13741

98

Cp2Fe2As2CO, 2S+1=1 - open-shell singlet, dispersion - adduct CO decoord.

C 7.18224 2.72314 8.97960

C 6.99026 4.14401 9.20797

C 6.38896 4.66442 8.00506

C 6.19931 3.63342 7.05286

C 6.71225 2.46050 7.65385

C 7.72741 1.59429 9.85818

C 7.58695 0.24501 9.13146

C 7.07171 5.04786 10.44179

C 5.73250 4.90743 11.19284

C 5.52497 3.72606 5.70441

C 5.45401 5.17884 5.22651

C 6.90460 1.47945 11.15205

C 9.22464 1.76580 10.16300

C 7.21962 6.52055 10.01567

C 8.23337 4.76702 11.39872

C 6.29522 2.89480 4.67476

C 4.09750 3.16880 5.85444

H 6.11406 5.69645 7.85709

H 6.77372 1.50052 7.16612

H 7.00276 2.35062 11.79885

H 7.23433 0.60508 11.72342

H 5.84227 1.35072 10.92015

H 9.79896 1.68210 9.23708

H 9.56142 0.97644 10.84478

H 9.46021 2.72933 10.60953

H 6.54101 0.01373 8.90413

H 7.96912 -0.54954 9.78018

H 8.16448 0.22512 8.20396

H 8.10046 6.66160 9.38328

H 7.33468 7.14355 10.90797

H 6.34279 6.89150 9.47857

H 8.19911 3.77235 11.83994

H 8.20036 5.48854 12.22171

H 9.19128 4.88710 10.88493

H 4.89633 5.17183 10.53756

H 5.71245 5.57797 12.05918

H 5.56931 3.88726 11.54689

H 7.31898 3.26378 4.58963

H 5.81721 2.95969 3.69178

H 6.34333 1.83873 4.95599

H 4.11830 2.12476 6.18260

H 3.56482 3.21659 4.89816

H 3.52930 3.74416 6.59239

H 4.85853 5.79740 5.90566

H 4.98621 5.22757 4.23808

H 6.45451 5.61679 5.15615

C 11.62984 5.04298 4.62385

C 12.52043 4.64021 5.68341

C 12.45111 3.19135 5.74122

C 11.51895 2.79261 4.73202

C 11.02619 3.91114 4.02110

C 13.45179 5.69064 6.29665

C 14.69479 5.76519 5.38697

C 13.17013 2.14726 6.59923

C 14.68966 2.24260 6.38183

C 10.14178 3.89822 2.79623

C 9.45818 2.53600 2.64269

C 13.88990 5.44106 7.74194

C 12.77707 7.07501 6.29132

C 12.81481 2.25972 8.09006

C 12.75329 0.73083 6.16504

C 9.08594 5.00715 2.86953

C 11.04595 4.14788 1.57409

H 11.20131 1.77638 4.56350

H 11.46752 6.06447 4.31729

H 13.02243 5.40000 8.40636

H 14.52445 6.27111 8.06954

H 14.46724 4.52617 7.86533

H 12.58098 7.44331 5.28112

H 13.44063 7.80036 6.77217

H 11.83351 7.05524 6.84325

H 15.23580 4.81640 5.36166

H 15.38029 6.54098 5.74589

H 14.40570 6.01299 4.36064

H 13.03489 3.23918 8.50771

H 13.36992 1.50842 8.66332

H 11.74658 2.07623 8.23022

H 11.68107 0.56637 6.29928

H 13.28412 -0.00061 6.78250

H 13.01335 0.53266 5.12009

H 14.93115 2.14113 5.31867

H 15.19369 1.43405 6.92221

H 15.11060 3.18506 6.73141

H 8.42777 4.86325 3.72928

H 8.47623 5.01306 1.95973

H 9.54871 5.99374 2.97030

H 11.53384 5.12549 1.64071

H 10.45701 4.12193 0.65044

H 11.82684 3.38388 1.50458

H 10.18939 1.74549 2.44537

H 8.75843 2.55742 1.80133

H 8.90341 2.26377 3.54362

As 10.27326 4.99287 8.31888

As 9.03861 5.88409 6.51024

Fe 10.50264 4.08881 6.15928

Fe 8.33413 3.91095 7.56245

C 9.36774 2.60932 6.60056

O 9.32724 1.44058 6.39193

98

Cp2Fe2As2CO, 2S+1=1 - open-shell singlet, dispersion - TS CO decoord.

C 7.17102 2.70990 8.98872

C 6.98693 4.13174 9.21723

C 6.37791 4.65361 8.01864

H 6.10628 5.68667 7.87188

C 6.17650 3.62253 7.06904

C 6.68946 2.44804 7.66703

H 6.74298 1.48733 7.17979

C 7.71800 1.57958 9.86422

C 6.90441 1.46963 11.16436

H 7.01136 2.34089 11.80965

H 7.23445 0.59425 11.73401

H 5.83979 1.34552 10.94056

C 9.21818 1.74484 10.15748

H 9.78501 1.65698 9.22737

H 9.55658 0.95509 10.83801

H 9.46147 2.70807 10.60049

C 7.56613 0.23017 9.14000

H 6.51748 0.00312 8.92109

H 7.95001 -0.56533 9.78655

H 8.13632 0.20690 8.20804

C 7.08186 5.03632 10.44954

C 7.23322 6.50794 10.02090

H 8.11005 6.64432 9.38197

H 7.35759 7.13127 10.91172

H 6.35419 6.88230 9.48981

C 8.24951 4.75121 11.39791

H 8.21448 3.75702 11.84015

H 8.22578 5.47349 12.22055

H 9.20404 4.86688 10.87683

C 5.74778 4.90263 11.21084

H 4.90786 5.17018 10.56166

H 5.73731 5.57409 12.07664

H 5.58265 3.88356 11.56716

C 5.49312 3.71675 5.72523

C 6.25481 2.88406 4.69031

H 7.27875 3.25103 4.59862

H 5.77042 2.95007 3.71054

H 6.30274 1.82788 4.97115

C 4.06558 3.16245 5.88473

H 4.08635 2.11833 6.21262

H 3.52659 3.21144 4.93204

H 3.50350 3.73889 6.62651

C 5.42210 5.16974 5.24800

H 4.83265 5.78953 5.93129

H 4.94763 5.21957 4.26281

H 6.42307 5.60544 5.17073

C 12.49158 4.59944 5.65240

C 12.41295 3.15100 5.70690

C 11.47131 2.76099 4.70307

H 11.14612 1.74726 4.53391

C 10.98136 3.88455 3.99822

C 11.59621 5.01082 4.59999

H 11.43905 6.03415 4.29683

C 13.43439 5.64219 6.26117

C 13.88243 5.38576 7.70223

H 13.02009 5.34863 8.37353

H 14.52511 6.21068 8.02691

H 14.45464 4.46671 7.81846

C 12.76843 7.03079 6.26490

H 12.56658 7.40306 5.25728

H 13.44034 7.75068 6.74236

H 11.82917 7.01538 6.82425

C 14.67061 5.71112 5.34190

H 15.20517 4.75886 5.30989

H 15.36400 6.48147 5.69741

H 14.37510 5.96350 4.31852

C 13.13083 2.10009 6.55751

C 12.78714 2.21209 8.05107

H 13.01664 3.18926 8.46900

H 13.34136 1.45600 8.61886

H 11.71877 2.03537 8.19843

C 12.70049 0.68757 6.12377

H 11.62801 0.53143 6.26552

H 13.23022 -0.04890 6.73619

H 12.95159 0.48930 5.07666

C 14.64933 2.18520 6.32899

H 14.88219 2.08359 5.26392

H 15.15179 1.37241 6.86446

H 15.07939 3.12427 6.67665

C 10.08825 3.88081 2.77957

C 9.03784 4.99402 2.86518

H 8.38502 4.84890 3.72881

H 8.42191 5.00681 1.95965

H 9.50563 5.97809 2.96741

C 10.98488 4.13170 1.55215

H 11.47774 5.10676 1.61953

H 10.38928 4.11254 0.63261

H 11.76173 3.36446 1.47372

C 9.39732 2.52245 2.62487

H 10.12358 1.72916 2.42051

H 8.69303 2.55045 1.78750

H 8.84605 2.24960 3.52777

As 9.02234 5.86273 6.50474

As 10.26605 4.96347 8.30309

Fe 8.31592 3.89224 7.56072

Fe 10.47378 4.06029 6.14075

O 9.28899 1.41707 6.38401

C 9.33539 2.58580 6.59156

98

Cp2Fe2As2CO, 2S+1=1 - open-shell singlet, dispersion - product CO decoord.

C 7.13469 2.73479 8.90009

C 6.76031 4.07133 9.36469

C 6.09576 4.69428 8.27167

C 6.06161 3.83503 7.14315

C 6.69807 2.63347 7.53790

C 7.77915 1.53876 9.60925

C 7.64296 0.27763 8.73965

C 6.87847 4.80650 10.70281

C 5.66472 4.44412 11.57899

C 5.36239 4.08108 5.82567

C 5.03039 5.56436 5.64388

C 7.06770 1.22489 10.93358

C 9.28606 1.75168 9.82056

C 6.84349 6.32791 10.46061

C 8.18122 4.53917 11.46525

C 6.24217 3.61564 4.66170

C 4.05501 3.26612 5.83463

H 5.73103 5.70936 8.27114

H 6.81476 1.76572 6.90774

H 7.18419 2.01441 11.67424

H 7.47603 0.30500 11.36605

H 5.99637 1.07516 10.76628

H 9.77907 1.83330 8.84672

H 9.72899 0.89929 10.34865

H 9.51337 2.65821 10.37943

H 6.59694 0.04810 8.51530

H 8.07367 -0.57732 9.27028

H 8.18538 0.38222 7.79632

H 7.62631 6.63103 9.75797

H 7.00993 6.85027 11.40795

H 5.87825 6.66588 10.07408

H 8.29257 3.50264 11.78039

H 8.20408 5.15552 12.37017

H 9.04554 4.80978 10.85041

H 4.73447 4.69740 11.06066

H 5.69429 5.00351 12.52096

H 5.63273 3.37925 11.81752

H 7.17828 4.18103 4.65259

H 5.73185 3.76894 3.70468

H 6.49064 2.55402 4.74616

H 4.26231 2.19653 5.93779

H 3.50076 3.42041 4.90202

H 3.41386 3.56852 6.66871

H 4.34821 5.92181 6.42176

H 4.54349 5.72409 4.67643

H 5.93804 6.17530 5.67556

C 11.86403 4.92024 4.33355

C 12.63404 4.61931 5.49091

C 12.29426 3.25232 5.86809

C 11.33749 2.78786 4.91174

C 11.05649 3.80764 3.96485

C 13.65021 5.66019 5.97446

C 14.91483 5.50499 5.10712

C 12.86235 2.29757 6.92156

C 14.36234 2.09007 6.64689

C 10.21194 3.70851 2.71266

C 9.26456 2.50997 2.79498

C 14.04643 5.58897 7.45024

C 13.08282 7.07812 5.76260

C 12.63342 2.74821 8.37131

C 12.18919 0.91950 6.80069

C 9.39549 4.99105 2.50550

C 11.16389 3.51682 1.51797

H 10.89660 1.80482 4.90835

H 11.87999 5.86333 3.80914

H 13.16957 5.69543 8.09487

H 14.72838 6.41596 7.67410

H 14.56795 4.66706 7.70692

H 12.92625 7.31719 4.70802

H 13.78940 7.81576 6.15632

H 12.13019 7.19757 6.28772

H 15.36738 4.51736 5.23293

H 15.66076 6.25981 5.38102

H 14.67405 5.62904 4.04682

H 13.10336 3.69793 8.60609

H 13.03285 1.99406 9.05878

H 11.56481 2.85620 8.56604

H 11.10980 0.98684 6.96841

H 12.60067 0.24923 7.56143

H 12.36166 0.45969 5.82320

H 14.51843 1.72880 5.62531

H 14.77356 1.34713 7.33915

H 14.93583 3.01013 6.76626

H 8.71613 5.16431 3.34542

H 8.80202 4.92129 1.58753

H 10.04282 5.86884 2.41941

H 11.84474 4.36812 1.42093

H 10.59742 3.42281 0.58457

H 11.76903 2.61371 1.64586

H 9.81970 1.56846 2.84797

H 8.62414 2.46909 1.90811

H 8.61950 2.58182 3.67474

As 10.22011 5.43637 8.13212

As 8.77589 6.16239 6.41863

Fe 10.38682 4.51917 5.91863

Fe 8.23868 4.21775 7.73340

C 8.26931 0.25455 5.18039

O 9.18363 -0.39500 4.99566

98

Cp2Fe2P2CO, 2S+1=3, dispersion - adduct CO decoord.

Fe 3.42572 4.02171 6.15820

Fe 1.38789 3.91368 7.47932

P 2.11634 5.81611 6.47825

P 3.21971 5.10698 8.11428

O 2.13125 1.43054 6.16964

C 2.31150 2.57705 6.42214

C 3.98395 3.84670 4.12983

C 4.54649 5.00911 4.69793

H 4.35218 6.01395 4.35686

C 5.41174 4.67636 5.80259

C 5.37234 3.23459 5.93863

C 4.48688 2.76848 4.91051

H 4.21417 1.73599 4.76192

C -0.76971 3.74440 7.11275

C -0.27586 2.54641 7.68476

H -0.29511 1.58512 7.19611

C 0.28222 2.78168 8.98010

C 0.18618 4.20931 9.21182

C -0.45869 4.76399 8.03796

H -0.67822 5.81062 7.90034

C 3.14199 3.77548 2.87740

C 2.45045 2.41360 2.76456

H 1.84029 2.19902 3.64412

H 3.18080 1.60500 2.65821

H 1.80344 2.39310 1.88171

C 2.09238 4.89231 2.86965

H 1.42597 4.80469 3.73042

H 1.49319 4.84521 1.95408

H 2.55806 5.88164 2.91379

C 4.08540 3.96029 1.67445

H 3.52610 3.89729 0.73426

H 4.86007 3.18671 1.66409

H 4.58171 4.93534 1.71219

C 6.32080 5.77365 6.36466

C 7.56784 5.79543 5.45548

H 8.11265 4.84946 5.49283

H 8.24764 6.59563 5.76859

H 7.28318 5.97707 4.41425

C 5.64702 7.15435 6.26233

H 5.45263 7.45162 5.22892

H 6.31282 7.90961 6.69133

H 4.70360 7.18135 6.81315

C 6.76078 5.61768 7.82151

H 5.89646 5.59082 8.49082

H 7.37478 6.48016 8.10113

H 7.36313 4.72633 7.99068

C 6.09575 2.25357 6.86279

C 5.66808 0.81274 6.53170

H 4.59105 0.67173 6.65658

H 6.17761 0.12406 7.21294

H 5.94191 0.52958 5.51024

C 5.74960 2.47127 8.34185

H 5.99715 3.46739 8.69780

H 6.28562 1.74303 8.96126

H 4.67793 2.33225 8.49177

C 7.61446 2.32833 6.63378

H 7.85296 2.16980 5.57685

H 8.11863 1.54875 7.21527

H 8.03853 3.28749 6.93268

C -1.50801 3.87188 5.80048

C -2.95422 3.39856 6.03550

H -2.97594 2.35606 6.36870

H -3.53550 3.47383 5.10993

H -3.44611 4.00893 6.79968

C -1.52081 5.32534 5.31931

H -2.04003 5.97924 6.02742

H -2.04237 5.39742 4.35969

H -0.50320 5.70531 5.18750

C -0.84854 2.99304 4.73309

H 0.18697 3.30147 4.57696

H -1.38451 3.08070 3.78244

H -0.84229 1.93725 5.01878

C 0.83365 1.62545 9.81791

C 2.35531 1.73653 9.99679

H 2.84149 1.61422 9.02615

H 2.71862 0.94419 10.66095

H 2.66774 2.69715 10.40222

C 0.57790 0.28687 9.10270

H -0.49162 0.10242 8.95644

H 0.97376 -0.52638 9.71905

H 1.08109 0.24430 8.13344

C 0.11505 1.53781 11.17378

H 0.31615 2.39123 11.82023

H 0.44331 0.63833 11.70543

H -0.96843 1.46880 11.03172

C 0.35330 5.09342 10.45005

C 1.57076 4.78295 11.32500

H 1.55252 3.77944 11.74686

H 1.59775 5.48694 12.16322

H 2.49560 4.90321 10.75393

C 0.47658 6.57341 10.04299

H 0.65863 7.17651 10.93773

H -0.43813 6.95517 9.58148

H 1.30814 6.72915 9.35081

C -0.94019 4.95021 11.27843

H -1.08858 3.92939 11.63507

H -1.81268 5.22533 10.67693

H -0.90383 5.61502 12.14850

98

Cp2Fe2P2CO, 2S+1=3, dispersion - TS CO decoord.

Fe 3.65795 4.45481 6.23799

Fe 1.12278 4.50586 7.48604

P 2.04363 6.00579 6.18458

P 3.05666 5.46055 8.08870

O 3.50084 -1.81364 6.85040

C 3.46745 -0.87470 6.21168

C 4.00559 3.29283 4.43772

C 4.69444 4.52018 4.42504

H 4.52778 5.31692 3.71776

C 5.59120 4.59822 5.55763

C 5.50094 3.31594 6.24897

C 4.48988 2.58021 5.56942

H 4.12920 1.60703 5.86503

C -0.98505 4.32755 7.06151

C -0.42081 3.03494 7.11501

H -0.40912 2.33989 6.29020

C 0.15776 2.77512 8.39960

C -0.01584 3.97645 9.18708

C -0.70321 4.91097 8.32702

H -0.95807 5.92235 8.60294

C 2.96820 2.80449 3.45423

C 1.83618 2.08643 4.19715

H 1.37582 2.76196 4.92379

H 2.20670 1.20639 4.73033

H 1.06944 1.75244 3.48972

C 2.38472 3.97601 2.65977

H 1.94376 4.71863 3.33166

H 1.60568 3.61906 1.97863

H 3.15056 4.47317 2.05579

C 3.65682 1.81819 2.49412

H 2.94028 1.43644 1.75839

H 4.07109 0.96620 3.04255

H 4.47540 2.30518 1.95462

C 6.48157 5.83169 5.72032

C 7.78554 5.59202 4.93564

H 8.35114 4.74415 5.32679

H 8.42414 6.48085 4.98648

H 7.56638 5.39056 3.88209

C 5.78683 7.06386 5.10919

H 5.66711 6.98219 4.02557

H 6.39890 7.95135 5.29733

H 4.80154 7.22311 5.55703

C 6.79221 6.20291 7.17330

H 5.86577 6.35793 7.73430

H 7.36352 7.13682 7.19229

H 7.38838 5.45181 7.68960

C 6.27318 2.70170 7.42096

C 5.94782 1.20139 7.52481

H 4.89729 1.03436 7.77343

H 6.54652 0.75643 8.32565

H 6.17868 0.67125 6.59515

C 5.90074 3.32264 8.77643

H 6.06581 4.39746 8.80862

H 6.49220 2.85847 9.57424

H 4.84177 3.15070 8.98505

C 7.78740 2.79485 7.17234

H 8.04856 2.34993 6.20663

H 8.32510 2.24808 7.95433

H 8.15324 3.82073 7.18024

C -1.77993 4.92741 5.92420

C -3.21583 4.37948 6.02549

H -3.22498 3.28829 5.93658

H -3.84205 4.79394 5.22764

H -3.66718 4.64382 6.98721

C -1.81004 6.45532 6.02794

H -2.29755 6.78594 6.95076

H -2.37342 6.87635 5.18891

H -0.79628 6.86629 6.00525

C -1.17746 4.52896 4.57262

H -0.15427 4.90429 4.48552

H -1.77320 4.94748 3.75460

H -1.15326 3.44272 4.44236

C 0.80488 1.42051 8.69811

C 2.33439 1.53601 8.78376

H 2.72895 1.91874 7.83729

H 2.77707 0.55298 8.98311

H 2.65939 2.22111 9.56461

C 0.50049 0.42891 7.56221

H -0.57615 0.30079 7.41211

H 0.91981 -0.54891 7.81747

H 0.94973 0.74498 6.61708

C 0.22441 0.79774 9.97678

H 0.46009 1.37127 10.87178

H 0.63145 -0.20980 10.11449

H -0.86491 0.71581 9.90496

C 0.24841 4.34359 10.64843

C 1.58416 3.83831 11.20396

H 1.65418 2.75193 11.23747

H 1.70958 4.20069 12.22974

H 2.41438 4.22347 10.60434

C 0.27828 5.87533 10.80744

H 0.54168 6.12462 11.83996

H -0.69450 6.33245 10.60592

H 1.02444 6.32492 10.14548

C -0.92208 3.81217 11.49808

H -0.99720 2.72381 11.46157

H -1.87062 4.22418 11.13871

H -0.79621 4.11067 12.54487

98

Cp2Fe2P2CO, 2S+1=3, dispersion - product CO decoord.

Fe 3.39823 4.00761 6.17617

Fe 1.33367 3.86871 7.45164

P 2.04413 5.79388 6.55309

P 3.12443 5.03642 8.18072

O 2.10329 1.40383 6.14566

C 2.15816 2.54173 6.48177

C 3.98603 3.81983 4.08631

C 4.52348 4.98052 4.68217

H 4.32588 5.99055 4.35975

C 5.40454 4.62771 5.77671

C 5.39953 3.17948 5.85004

C 4.50633 2.72956 4.83186

H 4.22975 1.70011 4.67055

C -0.73186 3.72296 7.07573

C -0.22524 2.52932 7.65427

H -0.23411 1.56685 7.16804

C 0.31967 2.76867 8.96123

C 0.16945 4.18673 9.20039

C -0.46902 4.73447 8.02794

H -0.69955 5.77932 7.89233

C 3.10507 3.75936 2.85925

C 2.45351 2.37953 2.72604

H 1.88213 2.11694 3.61917

H 3.20411 1.59880 2.56544

H 1.77554 2.36751 1.86686

C 2.01912 4.83945 2.92194

H 1.37602 4.69232 3.79255

H 1.39863 4.80740 2.02024

H 2.45121 5.84191 2.99680

C 4.00342 4.01457 1.63471

H 3.41475 3.96250 0.71213

H 4.80197 3.26816 1.57366

H 4.46800 5.00430 1.68823

C 6.30962 5.71640 6.36004

C 7.56432 5.76000 5.46293

H 8.11727 4.81847 5.49120

H 8.23438 6.56110 5.79388

H 7.28772 5.95534 4.42190

C 5.62615 7.09353 6.27607

H 5.45222 7.41293 5.24536

H 6.27654 7.84373 6.73617

H 4.67092 7.09616 6.80680

C 6.73078 5.53524 7.81987

H 5.85527 5.49529 8.47383

H 7.33857 6.39408 8.12283

H 7.33343 4.64280 7.98303

C 6.11257 2.18189 6.76504

C 5.71035 0.74534 6.38792

H 4.63423 0.58504 6.49894

H 6.22308 0.04553 7.05493

H 5.99918 0.49594 5.36175

C 5.72188 2.36293 8.23882

H 5.95112 3.35280 8.62387

H 6.24432 1.62412 8.85687

H 4.64738 2.20959 8.35706

C 7.63550 2.28214 6.57941

H 7.90601 2.14287 5.52759

H 8.13230 1.49982 7.16323

H 8.03785 3.24151 6.90538

C -1.49965 3.84678 5.78106

C -2.96544 3.47827 6.07475

H -3.04204 2.45409 6.45386

H -3.57076 3.55262 5.16439

H -3.39318 4.14977 6.82625

C -1.43600 5.27878 5.24239

H -1.88997 5.99137 5.93843

H -1.98126 5.34945 4.29558

H -0.40112 5.58717 5.07032

C -0.93481 2.88662 4.73057

H 0.11542 3.11146 4.53610

H -1.49029 2.97956 3.79159

H -0.99923 1.84374 5.05469

C 0.85065 1.61587 9.81635

C 2.36345 1.72868 10.05024

H 2.88172 1.59961 9.09805

H 2.70184 0.94266 10.73523

H 2.66181 2.69380 10.45441

C 0.62338 0.27582 9.09322

H -0.44078 0.08372 8.92028

H 1.00976 -0.53548 9.71845

H 1.14884 0.23728 8.13593

C 0.08400 1.51990 11.14569

H 0.26441 2.36740 11.80605

H 0.38903 0.61513 11.68248

H -0.99400 1.45653 10.96428

C 0.31787 5.07123 10.44097

C 1.51699 4.76400 11.34173

H 1.49227 3.76014 11.76171

H 1.52359 5.46761 12.18085

H 2.45551 4.88589 10.79385

C 0.44043 6.55428 10.04467

H 0.59008 7.15459 10.94739

H -0.46215 6.93103 9.55647

H 1.29100 6.72445 9.37969

C -0.99098 4.92148 11.24458

H -1.14369 3.89830 11.59213

H -1.85239 5.19790 10.62805

H -0.97215 5.58147 12.11897

98

Cp2Fe2P2CO, 2S+1=1 - open-shell singlet, dispersion - adduct CO decoord.

C 7.16915 2.72985 8.99393

C 6.99369 4.15368 9.20638

C 6.40580 4.67005 7.99345

H 6.15023 5.70520 7.83315

C 6.20795 3.63430 7.05038

C 6.70882 2.46163 7.66604

H 6.76163 1.49623 7.18798

C 7.71748 1.60656 9.87822

C 6.92151 1.51198 11.18988

H 7.04632 2.38611 11.82804

H 7.25297 0.63772 11.76038

H 5.85266 1.39509 10.98313

C 9.22145 1.77340 10.15185

H 9.77723 1.67838 9.21579

H 9.56928 0.98927 10.83413

H 9.47012 2.74042 10.58353

C 7.55368 0.25032 9.16967

H 6.50267 0.02860 8.95662

H 7.93466 -0.54088 9.82314

H 8.12035 0.21189 8.23596

C 7.08673 5.06640 10.43196

C 7.20726 6.53768 9.99323

H 8.06860 6.68738 9.33666

H 7.33929 7.16704 10.87866

H 6.31072 6.89438 9.47927

C 8.27244 4.80182 11.36402

H 8.24956 3.81266 11.81854

H 8.25698 5.53281 12.17922

H 9.21761 4.91617 10.82598

C 5.76415 4.91967 11.21073

H 4.91279 5.16935 10.56929

H 5.75452 5.59952 12.07001

H 5.61735 3.90240 11.57896

C 5.53265 3.72162 5.70243

C 6.27072 2.84484 4.68726

H 7.30815 3.17143 4.59673

H 5.79600 2.91249 3.70293

H 6.27662 1.79220 4.98515

C 4.08813 3.21428 5.86664

H 4.07580 2.17703 6.21626

H 3.55458 3.25960 4.91066

H 3.54096 3.82295 6.59367

C 5.50774 5.16725 5.19850

H 4.92850 5.81473 5.86464

H 5.04414 5.21290 4.20790

H 6.52048 5.57467 5.12514

C 12.49573 4.62528 5.68876

C 12.43507 3.17676 5.73120

C 11.49256 2.78531 4.72891

H 11.17951 1.76916 4.55086

C 10.99162 3.90913 4.02870

C 11.59352 5.03524 4.63995

H 11.42000 6.05995 4.35077

C 13.42531 5.67019 6.31210

C 13.82323 5.42463 7.76992

H 12.93868 5.39078 8.41177

H 14.45578 6.25094 8.11079

H 14.39106 4.50626 7.91076

C 12.76705 7.06188 6.27676

H 12.61390 7.42601 5.25749

H 13.42180 7.78174 6.77743

H 11.80413 7.05902 6.79441

C 14.68950 5.72706 5.43083

H 15.22596 4.77581 5.42682

H 15.37080 6.50288 5.79766

H 14.42520 5.96657 4.39582

C 13.14749 2.12886 6.59054

C 12.75397 2.22816 8.07313

H 12.94865 3.20899 8.50038

H 13.30343 1.48134 8.65765

H 11.68517 2.02956 8.18487

C 12.74973 0.71431 6.13306

H 11.67731 0.53836 6.25041

H 13.27767 -0.02033 6.74920

H 13.02589 0.53193 5.08932

C 14.67097 2.23451 6.41305

H 14.94079 2.15342 5.35499

H 15.16407 1.41798 6.95147

H 15.07884 3.17119 6.79205

C 10.10092 3.90600 2.80834

C 9.02605 4.99363 2.91442

H 8.37869 4.81880 3.77619

H 8.40807 5.00873 2.01025

H 9.47157 5.98590 3.03401

C 10.99181 4.19834 1.58599

H 11.46177 5.18333 1.67023

H 10.39712 4.18101 0.66581

H 11.78638 3.45086 1.49484

C 9.44158 2.53505 2.62876

H 10.18645 1.76259 2.41164

H 8.73768 2.56150 1.79104

H 8.89543 2.23299 3.52554

P 9.06897 5.76427 6.55758

P 10.19370 5.02244 8.20864

Fe 8.34198 3.88941 7.57363

Fe 10.49019 4.05961 6.17724

O 9.29858 1.41233 6.37563

C 9.34992 2.57762 6.59453

98

Cp2Fe2P2CO, 2S+1=1 - open-shell singlet, dispersion - TS CO decoord.

C 7.16769 2.70893 8.99111

C 6.99697 4.13284 9.20683

C 6.40394 4.65273 7.99790

H 6.15008 5.68878 7.84072

C 6.19861 3.61907 7.05416

C 6.69972 2.44408 7.66528

H 6.74745 1.47934 7.18536

C 7.71817 1.58282 9.87046

C 6.92886 1.48754 11.18608

H 7.05887 2.36024 11.82516

H 7.26142 0.61154 11.75327

H 5.85871 1.37330 10.98470

C 9.22392 1.74584 10.13653

H 9.77457 1.65168 9.19734

H 9.57364 0.95949 10.81527

H 9.47695 2.71138 10.56896

C 7.54776 0.22828 9.16023

H 6.49518 0.00911 8.95237

H 7.93061 -0.56495 9.81012

H 8.10933 0.19047 8.22340

C 7.09861 5.04319 10.43347

C 7.22061 6.51492 9.99664

H 8.07897 6.66350 9.33594

H 7.35875 7.14243 10.88245

H 6.32237 6.87477 9.48785

C 8.28837 4.77408 11.35904

H 8.26537 3.78418 11.81196

H 8.27882 5.50366 12.17560

H 9.23109 4.88710 10.81646

C 5.77967 4.89836 11.21875

H 4.92565 5.15114 10.58209

H 5.77607 5.57678 12.07920

H 5.63233 3.88081 11.58603

C 5.51652 3.71024 5.70991

C 6.24750 2.83368 4.68943

H 7.28532 3.15785 4.59468

H 5.76827 2.90453 3.70751

H 6.25219 1.78044 4.98517

C 4.07178 3.20568 5.88065

H 4.05904 2.16785 6.22854

H 3.53345 3.25378 4.92750

H 3.52965 3.81424 6.61153

C 5.49205 5.15684 5.20871

H 4.91763 5.80430 5.87903

H 5.02341 5.20525 4.22061

H 6.50523 5.56231 5.13077

C 12.48137 4.59663 5.66019

C 12.41592 3.14819 5.69802

C 11.46628 2.76339 4.69986

H 11.14879 1.74890 4.52016

C 10.96525 3.89128 4.00637

C 11.57451 5.01321 4.61803

H 11.40291 6.03948 4.33323

C 13.41809 5.63623 6.28169

C 13.82416 5.38393 7.73611

H 12.94340 5.34994 8.38312

H 14.46097 6.20721 8.07636

H 14.39039 4.46351 7.86996

C 12.76403 7.03010 6.25564

H 12.60560 7.39851 5.23873

H 13.42415 7.74606 6.75484

H 11.80437 7.02826 6.77932

C 14.67704 5.69237 5.39292

H 15.21026 4.73935 5.38193

H 15.36317 6.46449 5.75856

H 14.40722 5.93679 4.36047

C 13.12958 2.09492 6.54971

C 12.74523 2.19084 8.03491

H 12.94653 3.16936 8.46434

H 13.29498 1.43973 8.61365

H 11.67626 1.99633 8.15222

C 12.72407 0.68327 6.09010

H 11.65180 0.51063 6.21355

H 13.25331 -0.05518 6.70054

H 12.99312 0.50334 5.04407

C 14.65233 2.19573 6.36332

H 14.91544 2.11642 5.30344

H 15.14589 1.37616 6.89667

H 15.06570 3.13008 6.74214

C 10.06767 3.89585 2.79105

C 8.99547 4.98492 2.90872

H 8.35291 4.80695 3.77344

H 8.37220 5.00563 2.00830

H 9.44351 5.97576 3.03068

C 10.95210 4.19259 1.56511

H 11.42449 5.17622 1.65157

H 10.35209 4.18107 0.64830

H 11.74465 3.44403 1.46563

C 9.40462 2.52710 2.60838

H 10.14683 1.75411 2.38416

H 8.69678 2.55888 1.77417

H 8.86202 2.22208 3.50631

P 9.06285 5.74368 6.55196

P 10.19439 4.99368 8.19457

Fe 8.33597 3.86775 7.56631

Fe 10.47689 4.03610 6.15869

O 9.27830 1.39163 6.35550

C 9.33409 2.55605 6.57795

98

Cp2Fe2P2CO, 2S+1=1 - open-shell singlet, dispersion - product CO decoord.

C 7.05303 2.71146 8.38039

C 6.75379 3.87185 9.21384

C 6.10380 4.81629 8.36725

H 5.79031 5.80133 8.67544

C 6.02019 4.32878 7.04200

C 6.60855 3.03136 7.05944

H 6.65556 2.36826 6.21082

C 7.71160 1.35722 8.66769

C 7.11799 0.67607 9.90834

H 7.32412 1.21728 10.83045

H 7.54116 -0.32842 10.01698

H 6.03239 0.57715 9.80938

C 9.23763 1.50135 8.79421

H 9.64769 1.91853 7.86624

H 9.70406 0.52409 8.96574

H 9.53239 2.16288 9.60839

C 7.45957 0.39826 7.49075

H 6.39030 0.27172 7.29569

H 7.87634 -0.58443 7.73142

H 7.94241 0.74585 6.57338

C 6.95279 4.18020 10.69944

C 6.89239 5.70139 10.93182

H 7.63345 6.22361 10.31825

H 7.10634 5.91512 11.98367

H 5.90362 6.11471 10.71471

C 8.30941 3.72762 11.25385

H 8.44086 2.64671 11.23988

H 8.40400 4.05054 12.29602

H 9.12556 4.18304 10.68306

C 5.80193 3.54445 11.50195

H 4.83894 3.92596 11.14813

H 5.89789 3.79353 12.56478

H 5.78337 2.45768 11.40809

C 5.28282 4.96088 5.88241

C 5.98690 4.66272 4.55476

H 6.99739 5.08039 4.55293

H 5.42845 5.10336 3.72214

H 6.06494 3.58749 4.36942

C 3.86939 4.34713 5.85262

H 3.91732 3.26321 5.70746

H 3.28111 4.77751 5.03438

H 3.34254 4.53923 6.79266

C 5.16960 6.47744 6.06384

H 4.60402 6.73305 6.96548

H 4.64568 6.91956 5.21012

H 6.15987 6.93718 6.13580

C 12.67386 4.54746 5.40865

C 12.46842 3.32579 6.16772

C 11.44093 2.58472 5.49081

H 11.08156 1.61053 5.78079

C 11.01646 3.28973 4.33461

C 11.78535 4.48545 4.29593

H 11.67280 5.26171 3.55515

C 13.59718 5.75856 5.57221

C 13.84366 6.17416 7.02684

H 12.89599 6.37603 7.53535

H 14.44078 7.09201 7.04482

H 14.38841 5.42635 7.60104

C 12.96998 6.98977 4.88874

H 12.89628 6.87257 3.80417

H 13.59963 7.86581 5.07339

H 11.97069 7.19540 5.28500

C 14.93436 5.46735 4.86506

H 15.45733 4.61742 5.30722

H 15.59315 6.34124 4.92429

H 14.76343 5.24015 3.80790

C 13.19691 2.71758 7.37281

C 12.80792 3.37668 8.70589

H 13.00174 4.44747 8.71959

H 13.36585 2.91468 9.52873

H 11.73988 3.23565 8.89404

C 12.83421 1.22749 7.50398

H 11.77372 1.08807 7.72395

H 13.39885 0.79010 8.33338

H 13.08108 0.67051 6.59475

C 14.71958 2.76236 7.16531

H 14.99212 2.29305 6.21458

H 15.21862 2.21276 7.97070

H 15.11782 3.77569 7.16639

C 10.00374 2.81435 3.31545

C 9.42188 3.99865 2.53671

H 8.95090 4.71873 3.21325

H 8.66548 3.64935 1.82641

H 10.19453 4.52302 1.96569

C 10.71310 1.85808 2.34125

H 11.53620 2.36595 1.82859

H 10.01298 1.48770 1.58369

H 11.12721 0.99798 2.87659

C 8.86390 2.06965 4.02094

H 9.23055 1.17996 4.54065

H 8.10944 1.74767 3.29485

H 8.38094 2.72076 4.75694

P 8.98728 6.16494 6.30469

P 10.08379 5.63639 8.05441

Fe 8.20655 4.42848 7.61494

Fe 10.48918 4.47303 6.09228

O 10.49914 -1.79349 6.81974

C 10.42391 -0.88308 6.14407

98

Cp2Fe2As2CO, 2S+1=1, dispersion

As 9.03269 5.90279 6.46858

As 10.20807 5.03686 8.19982

Fe 8.33333 3.82480 7.34789

Fe 10.33965 3.94799 6.11110

O 9.00699 1.39455 5.95388

C 7.32686 2.69574 8.81693

C 7.19217 4.11091 9.10171

C 6.55924 4.69548 7.94964

H 6.33206 5.74441 7.84327

C 6.28547 3.70914 6.97091

C 6.76899 2.49279 7.51419

H 6.75573 1.54669 6.99800

C 7.86648 1.51884 9.63174

C 7.10592 1.37821 10.96058

H 7.28585 2.20347 11.64830

H 7.41702 0.45696 11.46461

H 6.02737 1.31589 10.78196

C 9.37934 1.63425 9.85759

H 9.88961 1.51772 8.89958

H 9.72580 0.84143 10.53020

H 9.67795 2.59561 10.27210

C 7.64180 0.20085 8.86854

H 6.57719 0.00519 8.70227

H 8.04144 -0.62587 9.46451

H 8.15636 0.19770 7.90486

C 7.32567 4.94815 10.37761

C 7.44024 6.44690 10.04199

H 8.29475 6.64781 9.39090

H 7.57640 7.01127 10.96957

H 6.53871 6.83539 9.56082

C 8.51514 4.61435 11.28198

H 8.50340 3.59075 11.65127

H 8.49751 5.27682 12.15354

H 9.45963 4.78003 10.75619

C 6.00800 4.75579 11.15768

H 5.15210 5.05186 10.54273

H 6.01256 5.38010 12.05795

H 5.85792 3.71865 11.46209

C 5.50838 3.87734 5.68710

C 6.02879 2.91761 4.61425

H 7.08393 3.10654 4.41226

H 5.46744 3.04821 3.68347

H 5.92931 1.87221 4.92036

C 4.03870 3.54270 6.00304

H 3.94115 2.51481 6.36674

H 3.42141 3.64819 5.10401

H 3.64140 4.21341 6.77179

C 5.59698 5.31618 5.17124

H 5.17106 6.02775 5.88583

H 5.03855 5.41421 4.23477

H 6.63472 5.60584 4.98599

C 12.31932 4.61451 5.78169

C 12.27203 3.16979 5.91279

C 11.40657 2.70064 4.87173

H 11.12945 1.66994 4.72211

C 10.90106 3.78229 4.10473

C 11.45158 4.94765 4.68545

H 11.25325 5.95188 4.34480

C 13.25178 5.70619 6.31988

C 13.74294 5.54939 7.75966

H 12.90233 5.52066 8.45855

H 14.36266 6.41410 8.01807

H 14.35491 4.66018 7.90532

C 12.58992 7.09378 6.23843

H 12.34966 7.38691 5.21354

H 13.28464 7.84341 6.62964

H 11.67381 7.13147 6.83250

C 14.46854 5.70968 5.36984

H 14.99702 4.75359 5.38606

H 15.17213 6.49576 5.66529

H 14.15468 5.90189 4.33907

C 12.98590 2.18741 6.84232

C 12.69944 2.44546 8.32650

H 13.03227 3.42166 8.66623

H 13.20168 1.68599 8.93606

H 11.62700 2.38352 8.51211

C 12.50101 0.75507 6.55623

H 11.42056 0.65896 6.69367

H 12.99244 0.06702 7.25096

H 12.75274 0.43217 5.54110

C 14.49802 2.21473 6.56317

H 14.69916 2.02739 5.50320

H 14.99871 1.43425 7.14635

H 14.95514 3.16909 6.82820

C 10.06147 3.73109 2.85045

C 8.97829 4.81422 2.88645

H 8.32285 4.67365 3.74792

H 8.37391 4.77907 1.97402

H 9.41257 5.81598 2.96207

C 11.00355 3.99595 1.66142

H 11.46815 4.98413 1.73987

H 10.44788 3.95362 0.71812

H 11.80215 3.24811 1.62162

C 9.41629 2.35309 2.67917

H 10.17519 1.57500 2.54698

H 8.77767 2.34717 1.79011

H 8.80863 2.08155 3.54413

C 9.15277 2.52367 6.30422

98

Cp2Fe2As2CO, 2S+1=5, dispersion

As 8.90853 5.87718 6.22664

As 10.10455 5.24891 8.07917

Fe 8.26924 3.87271 7.39427

Fe 10.38307 4.00137 6.04596

O 9.10055 1.41546 6.12501

C 7.17727 2.67626 8.84345

C 7.00954 4.07077 9.23597

C 6.33223 4.71021 8.15561

H 6.10627 5.76376 8.11049

C 6.06403 3.79357 7.11326

C 6.58760 2.54864 7.54243

H 6.56073 1.63577 6.96835

C 7.72982 1.45247 9.58138

C 7.02833 1.27096 10.93729

H 7.26399 2.05951 11.65069

H 7.33753 0.32043 11.38510

H 5.94150 1.24498 10.80772

C 9.25572 1.52727 9.75406

H 9.73755 1.42137 8.77960

H 9.60684 0.70981 10.39408

H 9.58640 2.47000 10.18731

C 7.44253 0.17333 8.77518

H 6.36734 0.01529 8.64022

H 7.83965 -0.68789 9.32221

H 7.92477 0.19687 7.79562

C 7.22999 4.84078 10.54150

C 7.32681 6.35120 10.25414

H 8.11860 6.56757 9.53119

H 7.55782 6.88174 11.18303

H 6.38766 6.76270 9.87475

C 8.49843 4.47105 11.31706

H 8.51312 3.43528 11.65177

H 8.57168 5.10238 12.20873

H 9.38855 4.64885 10.70681

C 5.98939 4.62324 11.43108

H 5.08495 4.94781 10.90685

H 6.07890 5.20981 12.35254

H 5.85739 3.57559 11.70616

C 5.31276 4.04229 5.82474

C 6.08637 3.45663 4.63741

H 7.05221 3.95849 4.53282

H 5.52285 3.58922 3.70773

H 6.27890 2.38758 4.76712

C 3.94548 3.34345 5.93969

H 4.06670 2.26474 6.07931

H 3.35540 3.50619 5.03109

H 3.37879 3.73339 6.79118

C 5.09821 5.54016 5.59494

H 4.50159 5.98789 6.39622

H 4.56568 5.70250 4.65242

H 6.05504 6.06956 5.54108

C 12.49984 4.63915 5.61372

C 12.42774 3.20514 5.83410

C 11.55945 2.67864 4.82502

H 11.27705 1.64244 4.73486

C 11.07033 3.72125 3.99813

C 11.65253 4.91420 4.49761

H 11.46815 5.89753 4.09517

C 13.39032 5.76120 6.15693

C 13.74983 5.68192 7.64300

H 12.84867 5.69249 8.26219

H 14.34933 6.55803 7.91111

H 14.33832 4.80073 7.89486

C 12.70622 7.12695 5.95652

H 12.57996 7.38084 4.90088

H 13.32735 7.91058 6.40123

H 11.72544 7.15124 6.43895

C 14.68476 5.75136 5.31944

H 15.23410 4.81374 5.43351

H 15.34154 6.57272 5.62818

H 14.45550 5.87732 4.25663

C 13.10093 2.27383 6.84534

C 12.68216 2.57281 8.29324

H 12.91377 3.58801 8.60421

H 13.18790 1.88162 8.97698

H 11.60422 2.43440 8.40484

C 12.68741 0.81644 6.57535

H 11.60628 0.67885 6.66600

H 13.17013 0.16676 7.31203

H 13.00107 0.48043 5.58186

C 14.62901 2.33945 6.68778

H 14.91893 2.10954 5.65733

H 15.10291 1.60314 7.34590

H 15.03716 3.31839 6.93956

C 10.23153 3.58129 2.74728

C 9.37771 4.83312 2.51864

H 8.70751 5.00972 3.36466

H 8.77117 4.71552 1.61483

H 9.99531 5.72716 2.38898

C 11.20516 3.41125 1.56556

H 11.86965 4.27682 1.47888

H 10.65198 3.30714 0.62548

H 11.82607 2.51959 1.69836

C 9.31777 2.35525 2.82890

H 9.89009 1.43032 2.94523

H 8.72873 2.26758 1.91011

H 8.63062 2.43039 3.67327

C 9.20056 2.57259 6.39471

96

Cp2Fe2As2, 2S+1=1, dispersion

As 6.09180 4.56872 9.62488

Fe 6.91502 6.57630 9.55082

C 5.55944 7.32778 8.11257

C 6.87308 7.94671 7.98743

C 7.12754 8.62245 9.22518

H 8.00866 9.20212 9.44497

C 6.07629 8.37747 10.14469

C 5.11727 7.59521 9.44894

H 4.19288 7.23802 9.87585

C 4.59508 6.72127 7.09125

C 3.42610 6.00953 7.79498

H 3.77749 5.20369 8.44515

H 2.76842 5.57096 7.03812

H 2.82029 6.70151 8.38687

C 3.97719 7.88786 6.29335

H 3.47150 8.58325 6.97104

H 3.23450 7.50337 5.58547

H 4.72160 8.45187 5.73220

C 5.22775 5.69888 6.14374

H 5.98175 6.13611 5.49022

H 4.45300 5.26426 5.50360

H 5.69188 4.88928 6.71609

C 7.90048 8.00202 6.85002

C 7.29918 8.61035 5.57342

H 8.09542 8.78796 4.84277

H 6.81578 9.56967 5.78482

H 6.56730 7.95473 5.10123

C 8.50113 6.62096 6.55445

H 9.08417 6.28399 7.41385

H 9.16108 6.67786 5.68102

H 7.74242 5.86477 6.36348

C 9.07945 8.90384 7.25419

H 8.76430 9.93698 7.43307

H 9.81483 8.91478 6.44380

H 9.58283 8.52596 8.14998

C 5.93619 8.92170 11.54745

C 7.24844 9.55136 12.02116

H 8.06463 8.82124 11.99164

H 7.14433 9.90497 13.05187

H 7.53204 10.40874 11.40205

C 5.55094 7.79499 12.50979

H 4.63065 7.29142 12.19787

H 5.39617 8.18960 13.51994

H 6.34505 7.04706 12.53591

C 4.83287 9.99381 11.52536

H 5.08215 10.79826 10.82564

H 4.70665 10.43288 12.52101

H 3.87391 9.56423 11.21886

As 8.92466 6.54813 10.40140

Fe 8.10144 4.54056 10.47546

C 9.45702 3.78907 11.91370

C 8.14340 3.17012 12.03884

C 7.88893 2.49441 10.80108

H 7.00781 1.91474 10.58129

C 8.94016 2.73940 9.88157

C 9.89919 3.52164 10.57733

H 10.82358 3.87884 10.15041

C 10.42140 4.39560 12.93498

C 11.59044 5.10718 12.23119

H 11.23912 5.91299 11.58095

H 12.24816 5.54576 12.98801

H 12.19619 4.41510 11.63936

C 11.03920 3.22905 13.73300

H 11.54485 2.53355 13.05538

H 11.78190 3.61355 14.44086

H 10.29475 2.66514 14.29420

C 9.78881 5.41815 13.88238

H 9.03475 4.98107 14.53592

H 10.56359 5.85276 14.52249

H 9.32479 6.22775 13.30993

C 7.11600 3.11476 13.17627

C 7.71719 2.50602 14.45272

H 6.92092 2.32840 15.18334

H 8.20040 1.54666 14.24110

H 8.44919 3.16140 14.92506

C 6.51560 4.49585 13.47215

H 5.93258 4.83310 12.61285

H 5.85567 4.43888 14.34560

H 7.27445 5.25188 13.66326

C 5.93686 2.21325 12.77192

H 6.25183 1.18010 12.59274

H 5.20152 2.20220 13.58235

H 5.43349 2.59145 11.87626

C 9.08026 2.19519 8.47880

C 7.76800 1.56553 8.00510

H 6.95181 2.29565 8.03465

H 7.87210 1.21195 6.97437

H 7.48442 0.70813 8.62418

C 9.46549 3.32194 7.51648

H 10.38579 3.82549 7.82840

H 9.62023 2.92736 6.50632

H 8.67138 4.06987 7.49040

C 10.18358 1.12309 8.50086

H 9.93432 0.31863 9.20057

H 10.30980 0.68405 7.50519

H 11.14255 1.55268 8.80735

96

Cp2Fe2As2, 2S+1=1 - open-shell singlet, dispersion

C 5.46405 7.31053 8.18000

C 6.78165 7.86661 7.89710

C 7.17880 8.59953 9.06572

H 8.09449 9.15874 9.16560

C 6.21812 8.43103 10.09795

C 5.16794 7.65704 9.53684

H 4.28237 7.35118 10.07181

C 4.37885 6.69704 7.29275

C 3.26441 6.06926 8.14846

H 3.65487 5.28351 8.80142

H 2.51735 5.61966 7.48719

H 2.74686 6.81314 8.76080

C 3.73135 7.85386 6.50405

H 3.32597 8.60222 7.19264

H 2.90605 7.47234 5.89271

H 4.43942 8.35627 5.84502

C 4.86445 5.60484 6.33639

H 5.56043 5.97627 5.58525

H 4.00722 5.18003 5.80385

H 5.35132 4.80072 6.89714

C 7.69958 7.79097 6.67127

C 7.01923 8.36116 5.41726

H 7.74966 8.43302 4.60445

H 6.62617 9.36539 5.60671

H 6.20024 7.73519 5.06258

C 8.18747 6.35614 6.41621

H 8.78322 6.01823 7.26672

H 8.80820 6.32823 5.51330

H 7.37195 5.64662 6.29471

C 8.96141 8.63669 6.91339

H 8.72437 9.69624 7.05445

H 9.61905 8.55567 6.04250

H 9.52057 8.27857 7.78384

C 6.24076 9.00492 11.49664

C 7.60719 9.61716 11.81294

H 8.40086 8.86743 11.72415

H 7.61658 10.00441 12.83673

H 7.84228 10.44835 11.13997

C 5.93851 7.90086 12.51765

H 4.96802 7.43128 12.33013

H 5.92460 8.31438 13.53200

H 6.69842 7.11793 12.45693

C 5.16085 10.09807 11.57511

H 5.35005 10.88883 10.84175

H 5.14846 10.55187 12.57201

H 4.16688 9.68435 11.37759

C 9.42107 3.82353 12.00921

C 8.14108 3.12770 11.97672

C 8.03439 2.52913 10.67724

H 7.21175 1.91803 10.34209

C 9.15079 2.89201 9.87531

C 10.00696 3.65379 10.71480

H 10.95054 4.07355 10.40602

C 10.23355 4.46627 13.13505

C 11.38941 5.30819 12.56732

H 11.02331 6.10519 11.91375

H 11.93324 5.77117 13.39649

H 12.10880 4.70183 12.00974

C 10.88328 3.33020 13.95180

H 11.51676 2.71423 13.30537

H 11.51472 3.75349 14.74077

H 10.14854 2.67567 14.41925

C 9.42701 5.39555 14.04548

H 8.67178 4.86938 14.62815

H 10.10069 5.88683 14.75523

H 8.93265 6.16909 13.44943

C 7.03600 2.93106 13.02376

C 7.59409 2.28897 14.30430

H 6.76507 1.99272 14.95558

H 8.17829 1.39242 14.07299

H 8.22440 2.97210 14.87390

C 6.30260 4.23823 13.36721

H 5.72409 4.58097 12.50597

H 5.61538 4.06720 14.20393

H 6.97957 5.04470 13.63817

C 5.96824 1.96754 12.47816

H 6.38249 0.97776 12.26002

H 5.18215 1.84312 13.22932

H 5.49779 2.36481 11.57320

C 9.42815 2.45725 8.45464

C 8.15435 2.51889 7.60432

H 7.78844 3.54672 7.54181

H 8.35972 2.15348 6.59237

H 7.35483 1.90276 8.02733

C 10.49542 3.35408 7.81994

H 11.45990 3.25629 8.32859

H 10.64489 3.07636 6.77178

H 10.19078 4.40373 7.85892

C 9.94067 1.00689 8.50825

H 9.18509 0.34031 8.93634

H 10.18163 0.64844 7.50150

H 10.84367 0.93383 9.12311

As 6.08196 4.61266 9.75907

As 9.04515 6.55684 10.20602

Fe 6.95060 6.58391 9.51830

Fe 8.16428 4.59421 10.47843

96

Cp2Fe2As2, 2S+1=3, dispersion

As 6.12821 4.43404 9.58212

Fe 6.90359 6.54148 9.52349

C 5.50040 7.43636 8.07909

C 6.78262 8.07414 7.88962

C 7.11350 8.69517 9.13493

H 8.01867 9.24784 9.32643

C 6.07148 8.51362 10.08474

C 5.09743 7.72614 9.44100

H 4.17411 7.39116 9.88817

C 4.49512 6.81280 7.10809

C 3.35649 6.11721 7.87498

H 3.74072 5.34116 8.54329

H 2.67977 5.64146 7.15837

H 2.76204 6.82467 8.45979

C 3.85176 7.95573 6.29774

H 3.37824 8.67656 6.97205

H 3.07978 7.55623 5.63027

H 4.57993 8.49564 5.69222

C 5.09004 5.75826 6.16926

H 5.83748 6.16512 5.48930

H 4.29449 5.32205 5.55596

H 5.55173 4.95246 6.74968

C 7.79348 8.06374 6.73666

C 7.18744 8.58790 5.42663

H 7.97147 8.67608 4.66670

H 6.74467 9.57852 5.57205

H 6.41867 7.92856 5.02408

C 8.38564 6.65817 6.53574

H 8.92728 6.35269 7.43635

H 9.09044 6.65697 5.69609

H 7.62363 5.90442 6.34212

C 8.97915 8.98453 7.07264

H 8.65861 10.01822 7.23822

H 9.68535 8.98186 6.23646

H 9.51897 8.63642 7.95893

C 6.02260 9.02277 11.50954

C 7.35215 9.67567 11.89631

H 8.17894 8.96298 11.80117

H 7.31552 10.01691 12.93587

H 7.57216 10.54424 11.26691

C 5.75069 7.85410 12.46637

H 4.81576 7.34084 12.22057

H 5.68158 8.20751 13.50124

H 6.55941 7.12006 12.40127

C 4.89136 10.05831 11.61966

H 5.06198 10.89634 10.93619

H 4.82929 10.45419 12.63961

H 3.92448 9.61071 11.36899

As 8.97738 6.46241 10.43787

Fe 8.12356 4.47748 10.47557

C 9.45819 3.72888 11.97570

C 8.14897 3.09596 12.05111

C 7.94930 2.42563 10.79776

H 7.08574 1.83251 10.54521

C 9.03396 2.68243 9.91771

C 9.95355 3.47359 10.65367

H 10.89225 3.83953 10.26757

C 10.38214 4.33433 13.03458

C 11.56736 5.06461 12.37869

H 11.23002 5.87428 11.72564

H 12.19441 5.50084 13.16230

H 12.20019 4.38601 11.79977

C 10.98339 3.16195 13.83767

H 11.51599 2.47849 13.16850

H 11.70019 3.54386 14.57297

H 10.22588 2.58603 14.36843

C 9.71125 5.33964 13.97401

H 8.93955 4.88942 14.59714

H 10.46204 5.77040 14.64453

H 9.26040 6.15282 13.39619

C 7.08218 3.02897 13.15074

C 7.64125 2.41564 14.44458

H 6.82110 2.23288 15.14681

H 8.13296 1.45808 14.24472

H 8.35558 3.06968 14.94480

C 6.46455 4.40706 13.43465

H 5.90787 4.74763 12.55864

H 5.77711 4.33991 14.28586

H 7.21181 5.16657 13.65446

C 5.92397 2.12282 12.70004

H 6.25134 1.09220 12.52861

H 5.15947 2.10426 13.48279

H 5.45176 2.50211 11.78804

C 9.23491 2.15390 8.51610

C 7.95974 1.48308 8.00058

H 7.11970 2.18617 8.00997

H 8.10594 1.13953 6.97166

H 7.68759 0.61303 8.60711

C 9.61080 3.30115 7.57153

H 10.49811 3.83851 7.92000

H 9.81822 2.91595 6.56715

H 8.79008 4.01960 7.51580

C 10.37434 1.12049 8.56194

H 10.13393 0.30270 9.24912

H 10.54303 0.69338 7.56748

H 11.30925 1.58039 8.89737

96

Cp2Fe2As2, 2S+1=5, dispersion

As 6.07172 4.49994 9.52339

Fe 6.90641 6.56729 9.55069

C 5.52859 7.44848 8.03990

C 6.80168 8.10797 7.89566

C 7.08425 8.71963 9.15924

H 7.96766 9.29681 9.37977

C 6.01314 8.51640 10.07401

C 5.07878 7.71305 9.39474

H 4.14530 7.36156 9.80676

C 4.55828 6.83270 7.02880

C 3.39715 6.12116 7.74573

H 3.75875 5.33152 8.41101

H 2.74496 5.65854 6.99860

H 2.78285 6.81597 8.32506

C 3.93548 7.98839 6.21961

H 3.43707 8.69494 6.89112

H 3.18694 7.59694 5.52147

H 4.67963 8.54187 5.64692

C 5.18757 5.79851 6.08984

H 5.95012 6.22291 5.43792

H 4.41263 5.36698 5.44769

H 5.63992 4.98718 6.67009

C 7.85126 8.11713 6.77778

C 7.28747 8.65612 5.45480

H 8.09729 8.76045 4.72456

H 6.83286 9.64166 5.59846

H 6.53837 7.99726 5.01578

C 8.45723 6.71690 6.57704

H 8.97832 6.40418 7.48728

H 9.18285 6.73129 5.75554

H 7.70561 5.96079 6.35534

C 9.02035 9.03824 7.16680

H 8.69100 10.06950 7.33078

H 9.75683 9.04630 6.35728

H 9.52782 8.68086 8.06857

C 5.89811 9.02427 11.49575

C 7.18594 9.73187 11.92447

H 8.04339 9.05220 11.86476

H 7.09876 10.07651 12.95985

H 7.39281 10.60527 11.29726

C 5.63807 7.84865 12.44827

H 4.74254 7.28800 12.16217

H 5.50271 8.20534 13.47533

H 6.48437 7.15544 12.42937

C 4.72329 10.01441 11.56279

H 4.88200 10.85498 10.87957

H 4.61407 10.41281 12.57768

H 3.78297 9.52759 11.28571

As 8.94474 6.61691 10.50287

Fe 8.11005 4.54955 10.47556

C 9.48788 3.66836 11.98634

C 8.21479 3.00888 12.13059

C 7.93221 2.39721 10.86700

H 7.04881 1.82003 10.64649

C 9.00332 2.60044 9.95224

C 9.93769 3.40379 10.63150

H 10.87117 3.75528 10.21948

C 10.45819 4.28414 12.99743

C 11.61924 4.99579 12.28050

H 11.25757 5.78545 11.61529

H 12.27146 5.45839 13.02762

H 12.23355 4.30105 11.70109

C 11.08107 3.12845 13.80655

H 11.57949 2.42195 13.13499

H 11.82963 3.51990 14.50468

H 10.33698 2.57490 14.37924

C 9.82885 5.31824 13.93647

H 9.06635 4.89376 14.58838

H 10.60378 5.74979 14.57863

H 9.37644 6.12958 13.35627

C 7.16524 2.99972 13.24849

C 7.72903 2.46062 14.57142

H 6.91922 2.35631 15.30169

H 8.18357 1.47506 14.42769

H 8.47819 3.11941 15.01046

C 6.55938 4.39999 13.44933

H 6.03827 4.71279 12.53914

H 5.83380 4.38562 14.27087

H 7.31107 5.15603 13.67104

C 5.99606 2.07872 12.85946

H 6.32533 1.04746 12.69532

H 5.25966 2.07060 13.66906

H 5.48851 2.43624 11.95780

C 9.11835 2.09257 8.53049

C 7.83050 1.38497 8.10178

H 6.97306 2.06465 8.16150

H 7.91768 1.04034 7.06640

H 7.62363 0.51157 8.72899

C 9.37838 3.26819 7.57798

H 10.27391 3.82884 7.86406

H 9.51373 2.91150 6.55091

H 8.53208 3.96140 7.59689

C 10.29316 1.10243 8.46344

H 10.13446 0.26186 9.14666

H 10.40237 0.70403 7.44855

H 11.23348 1.58925 8.74052

98

Cp2Fe2P2CO, 2S+1=1, dispersion

Fe 3.35537 3.96104 6.15646

Fe 1.36521 3.84970 7.39030

P 2.07340 5.81957 6.51565

P 3.15504 5.09661 8.10872

O 2.05040 1.38539 6.05530

C 2.18738 2.52278 6.37872

C 3.92495 3.77234 4.15329

C 4.45503 4.95424 4.71774

H 4.23282 5.95071 4.36958

C 5.32521 4.65103 5.82122

C 5.30106 3.20926 5.97490

C 4.44347 2.71021 4.93994

H 4.18464 1.67275 4.80443

C -0.68677 3.75202 7.03375

C -0.21031 2.53397 7.58063

H -0.23994 1.58426 7.07181

C 0.35836 2.73875 8.87954

C 0.24437 4.15656 9.15370

C -0.39193 4.74113 8.00301

H -0.59727 5.79355 7.88695

C 3.08017 3.68895 2.90411

C 2.44656 2.30224 2.76233

H 1.84151 2.04433 3.63344

H 3.21126 1.52740 2.64541

H 1.80707 2.27281 1.87436

C 1.98586 4.76112 2.92822

H 1.33998 4.62930 3.79806

H 1.37424 4.70145 2.02191

H 2.40885 5.76898 2.98115

C 4.01297 3.94088 1.70508

H 3.45209 3.87723 0.76608

H 4.81784 3.19938 1.67336

H 4.46931 4.93427 1.76354

C 6.23176 5.76537 6.35509

C 7.47162 5.76327 5.43522

H 8.01529 4.81739 5.48979

H 8.15471 6.56882 5.72639

H 7.18026 5.92287 4.39229

C 5.55611 7.14214 6.21975

H 5.35419 7.41009 5.17973

H 6.22633 7.90847 6.62111

H 4.61722 7.18454 6.77657

C 6.68755 5.64816 7.81047

H 5.83129 5.62047 8.48983

H 7.28871 6.52744 8.06392

H 7.31013 4.77243 7.98961

C 6.01694 2.25520 6.93194

C 5.56806 0.80906 6.65680

H 4.48792 0.69022 6.77826

H 6.06266 0.14091 7.36849

H 5.84363 0.47931 5.64998

C 5.68823 2.53297 8.40402

H 5.99420 3.52120 8.73382

H 6.18753 1.79318 9.03974

H 4.61264 2.45662 8.56220

C 7.53420 2.30871 6.68828

H 7.76510 2.11156 5.63617

H 8.03523 1.54568 7.29379

H 7.96635 3.27482 6.95219

C -1.46756 3.92171 5.75251

C -2.94452 3.63667 6.08326

H -3.07014 2.61933 6.46753

H -3.56396 3.74297 5.18580

H -3.31719 4.33369 6.84084

C -1.33894 5.34876 5.21255

H -1.73811 6.08395 5.91853

H -1.90191 5.44841 4.27891

H -0.29471 5.60455 5.01611

C -0.98428 2.92750 4.69327

H 0.07732 3.07186 4.48694

H -1.54142 3.06604 3.76115

H -1.12515 1.89128 5.01449

C 0.89202 1.55915 9.69595

C 2.41303 1.64103 9.87906

H 2.89632 1.49288 8.91155

H 2.75757 0.85294 10.55826

H 2.74422 2.60276 10.26706

C 0.61356 0.23749 8.95694

H -0.45952 0.07071 8.81520

H 1.00209 -0.59194 9.55643

H 1.10863 0.20564 7.98363

C 0.16935 1.45250 11.04869

H 0.39815 2.27610 11.72373

H 0.46818 0.52566 11.54980

H -0.91580 1.42216 10.90556

C 0.42284 5.00221 10.41741

C 1.64785 4.67012 11.27404

H 1.64030 3.65157 11.65765

H 1.67324 5.34333 12.13725

H 2.57020 4.81809 10.70555

C 0.52427 6.49825 10.06610

H 0.69713 7.06887 10.98371

H -0.39711 6.88212 9.61983

H 1.35179 6.69804 9.38105

C -0.86471 4.82283 11.24909

H -1.00773 3.79070 11.57191

H -1.74216 5.11562 10.66348

H -0.82379 5.45798 12.14086

98

Cp2Fe2P2CO, 2S+1=5, dispersion

Fe 3.40014 4.01555 6.09713

Fe 1.30362 3.89452 7.44626

P 1.96837 5.80926 6.31817

P 3.05987 5.29001 8.00075

O 2.13625 1.41449 6.21373

C 2.23187 2.57477 6.46728

C 4.08482 3.73054 4.04451

C 4.65802 4.92948 4.53665

H 4.46248 5.90992 4.13248

C 5.50971 4.66652 5.65348

C 5.44924 3.23413 5.88210

C 4.58029 2.69606 4.87884

H 4.30681 1.65695 4.79376

C -0.90232 3.81753 7.17947

C -0.37987 2.57917 7.62877

H -0.41438 1.65572 7.07228

C 0.21562 2.72774 8.92606

C 0.05630 4.12913 9.29163

C -0.62433 4.75189 8.20258

H -0.84480 5.80566 8.13932

C 3.23849 3.57902 2.79999

C 2.35125 2.33354 2.88327

H 1.67297 2.38624 3.73672

H 2.94477 1.42004 2.98371

H 1.75244 2.24086 1.97134

C 2.35749 4.81480 2.58655

H 1.69044 4.97292 3.43843

H 1.74658 4.69088 1.68647

H 2.95610 5.72175 2.45795

C 4.20408 3.43471 1.60836

H 3.64428 3.32221 0.67315

H 4.84551 2.55625 1.73147

H 4.84870 4.31488 1.51883

C 6.38972 5.79667 6.19520

C 7.70217 5.77543 5.38612

H 8.25148 4.84181 5.52711

H 8.35017 6.60373 5.69485

H 7.49509 5.88329 4.31674

C 5.71239 7.15908 5.95386

H 5.61893 7.39529 4.89060

H 6.32127 7.94931 6.40378

H 4.71773 7.19483 6.40614

C 6.71431 5.74001 7.69057

H 5.80017 5.74989 8.29045

H 7.30209 6.62347 7.96082

H 7.30450 4.86682 7.96567

C 6.12150 2.31550 6.90592

C 5.73241 0.85152 6.63522

H 4.65291 0.69683 6.71688

H 6.21830 0.21060 7.37746

H 6.06008 0.51900 5.64503

C 5.67155 2.61823 8.34377

H 5.89198 3.63601 8.65457

H 6.16501 1.93229 9.04166

H 4.59207 2.47644 8.43306

C 7.65101 2.39974 6.77746

H 7.96421 2.17369 5.75300

H 8.12059 1.66886 7.44465

H 8.04246 3.38301 7.03842

C -1.65195 4.04779 5.88659

C -3.03024 3.37436 6.01828

H -2.92647 2.29746 6.18387

H -3.61826 3.52476 5.10618

H -3.59001 3.79380 6.86029

C -1.84075 5.54363 5.62267

H -2.43219 6.01851 6.41215

H -2.36929 5.69340 4.67579

H -0.87552 6.05610 5.55971

C -0.88869 3.42229 4.71286

H 0.08636 3.90322 4.59686

H -1.44940 3.54524 3.78015

H -0.71624 2.35275 4.86508

C 0.76613 1.51297 9.68180

C 2.29515 1.57115 9.83088

H 2.76188 1.43951 8.85240

H 2.64440 0.76103 10.48121

H 2.64482 2.51655 10.24273

C 0.45205 0.22146 8.90569

H -0.62656 0.07548 8.78541

H 0.84396 -0.63383 9.46557

H 0.92396 0.21809 7.92066

C 0.08592 1.36724 11.05256

H 0.34977 2.16379 11.74710

H 0.38724 0.41953 11.51157

H -1.00344 1.35819 10.94396

C 0.30497 4.92478 10.57586

C 1.59542 4.56884 11.32098

H 1.61107 3.54274 11.68488

H 1.70120 5.22366 12.19228

H 2.46707 4.72099 10.67815

C 0.38945 6.42956 10.25718

H 0.64486 6.97781 11.16925

H -0.56248 6.83033 9.89829

H 1.15907 6.63697 9.50830

C -0.91098 4.72532 11.50284

H -1.03526 3.68419 11.80414

H -1.82947 5.03880 10.99648

H -0.79705 5.33164 12.40869

96

Cp2Fe2P2, 2S+1=1, dispersion

Fe 11.54370 6.04795 3.61573

Fe 9.19594 6.76747 3.45105

P 10.35664 5.99892 1.90482

P 10.37590 6.79544 5.16153

C 13.36537 6.30033 4.67399

C 13.47657 6.68409 3.27259

C 13.21136 5.50393 2.49910

H 13.22970 5.45310 1.42271

C 12.83429 4.43508 3.35142

C 12.93204 4.93658 4.67625

H 12.69487 4.36855 5.56224

C 7.74538 8.23483 3.88789

C 7.77627 7.95184 2.45736

C 7.46945 6.55975 2.31438

H 7.41897 6.03083 1.37698

C 7.31570 5.95206 3.58880

C 7.44259 6.99804 4.54152

H 7.36765 6.86716 5.60953

C 13.82644 6.95111 5.98026

C 13.48985 8.43565 6.14018

H 13.99725 9.06608 5.41091

H 13.80563 8.77109 7.13352

H 12.41120 8.59852 6.05527

C 13.21493 6.22661 7.19302

H 12.12195 6.24456 7.15902

H 13.53556 6.73099 8.10978

H 13.55035 5.18817 7.26387

C 15.35388 6.74892 6.05711

H 15.60026 5.68333 6.00944

H 15.73668 7.14515 7.00427

H 15.87794 7.24935 5.24145

C 13.80425 8.01447 2.58791

C 12.76138 9.09597 2.91006

H 13.03629 10.03393 2.41375

H 12.66556 9.28835 3.97574

H 11.78127 8.77874 2.55156

C 13.77698 7.83579 1.06049

H 14.53891 7.12942 0.71536

H 13.97834 8.79992 0.58363

H 12.79592 7.49417 0.71546

C 15.21769 8.49247 2.95556

H 15.95833 7.71026 2.75874

H 15.30146 8.78313 4.00309

H 15.48085 9.36701 2.35100

C 12.49286 3.01175 2.97434

C 11.22592 2.56098 3.71062

H 11.34107 2.63561 4.79635

H 10.99429 1.51851 3.46708

H 10.37939 3.18893 3.42353

C 13.67773 2.12320 3.39449

H 14.59910 2.43807 2.89383

H 13.48426 1.07805 3.12949

H 13.84524 2.17537 4.47492

C 12.27327 2.88072 1.46567

H 11.46198 3.53392 1.12833

H 12.00932 1.84858 1.21369

H 13.17758 3.13887 0.90476

C 7.81188 9.53076 4.69773

C 8.92272 10.48491 4.25076

H 8.98470 11.32769 4.94711

H 8.74913 10.89926 3.25811

H 9.88748 9.96784 4.24881

C 6.43414 10.21883 4.61715

H 6.43561 11.12820 5.22832

H 5.65582 9.55288 5.00362

H 6.16069 10.49461 3.59935

C 8.06199 9.23500 6.18680

H 8.99289 8.68119 6.33908

H 7.24336 8.66770 6.63878

H 8.13434 10.18222 6.73032

C 7.99844 8.83202 1.21874

C 9.44167 9.34295 1.08888

H 9.50633 10.06381 0.26562

H 10.11455 8.51011 0.86913

H 9.80446 9.82338 1.99409

C 7.71719 8.01513 -0.05442

H 7.87581 8.65266 -0.92978

H 6.68506 7.65133 -0.08767

H 8.39799 7.16287 -0.14127

C 7.02108 10.01878 1.20551

H 7.26281 10.76553 1.96219

H 5.99177 9.68346 1.36822

H 7.06398 10.51763 0.23155

C 6.98824 4.50578 3.87914

C 7.75653 4.01849 5.11362

H 7.51115 4.61059 6.00069

H 8.83428 4.09408 4.94913

H 7.50147 2.97507 5.32764

C 7.34534 3.62046 2.68146

H 8.40127 3.72996 2.41698

H 6.74498 3.87157 1.80111

H 7.15681 2.56920 2.92137

C 5.47505 4.41690 4.14692

H 5.18220 3.38091 4.34961

H 4.90341 4.77153 3.28305

H 5.19427 5.02650 5.01179

96

Cp2Fe2P2, 2S+1=1 - open-shell singlet, dispersion

C 5.49481 7.29302 8.18816

C 6.80792 7.85730 7.90010

C 7.20966 8.58032 9.07133

H 8.13388 9.12424 9.17661

C 6.25523 8.40520 10.10738

C 5.20080 7.63668 9.54553

H 4.31750 7.32758 10.08215

C 4.41660 6.65358 7.31129

C 3.32533 6.00150 8.17873

H 3.74102 5.23178 8.83504

H 2.58552 5.52906 7.52510

H 2.79204 6.73582 8.78928

C 3.73351 7.78899 6.52173

H 3.31469 8.53051 7.20966

H 2.91309 7.38330 5.91935

H 4.42261 8.30603 5.85392

C 4.92233 5.56943 6.35592

H 5.59692 5.95709 5.59337

H 4.07120 5.11742 5.83603

H 5.43967 4.78148 6.91176

C 7.71317 7.80632 6.66360

C 7.00399 8.37435 5.42421

H 7.72219 8.47207 4.60323

H 6.58979 9.36678 5.62979

H 6.19556 7.73289 5.07308

C 8.22508 6.38456 6.38476

H 8.86099 6.05468 7.20867

H 8.81301 6.37528 5.45971

H 7.42237 5.65714 6.28829

C 8.96114 8.67540 6.89515

H 8.70373 9.72813 7.05107

H 9.60580 8.61727 6.01267

H 9.54284 8.32170 7.75217

C 6.29011 8.96933 11.50936

C 7.67963 9.51802 11.84191

H 8.44018 8.73497 11.75393

H 7.69649 9.89765 12.86853

H 7.95755 10.34320 11.17804

C 5.93084 7.87517 12.52201

H 4.93996 7.45304 12.32840

H 5.93111 8.28358 13.53847

H 6.65306 7.05761 12.46167

C 5.25832 10.10837 11.58412

H 5.48994 10.89478 10.85830

H 5.25459 10.55586 12.58398

H 4.24975 9.73922 11.37258

C 9.38724 3.84892 12.00131

C 8.11187 3.14235 11.97153

C 8.00950 2.54519 10.67258

H 7.18021 1.94744 10.32977

C 9.12686 2.90646 9.87258

C 9.98072 3.67004 10.71188

H 10.92100 4.09639 10.40211

C 10.18662 4.52186 13.11862

C 11.31629 5.38912 12.53605

H 10.92715 6.16128 11.86619

H 11.84576 5.88282 13.35685

H 12.05438 4.79462 11.99021

C 10.86848 3.41397 13.94718

H 11.51513 2.80589 13.30634

H 11.49168 3.86312 14.72842

H 10.15214 2.74694 14.42591

C 9.35738 5.43820 14.02172

H 8.62427 4.89534 14.61740

H 10.01994 5.95976 14.72026

H 8.83375 6.18853 13.42132

C 7.01501 2.92391 13.02296

C 7.59593 2.28733 14.29628

H 6.77880 1.96968 14.95253

H 8.19853 1.40546 14.05604

H 8.21550 2.98195 14.86394

C 6.25583 4.21186 13.38082

H 5.66602 4.55179 12.52604

H 5.57408 4.01684 14.21679

H 6.91585 5.02872 13.66144

C 5.96325 1.94179 12.47894

H 6.39706 0.96280 12.25033

H 5.18679 1.79487 13.23613

H 5.47674 2.33455 11.58086

C 9.39712 2.47401 8.44993

C 8.12619 2.57940 7.59886

H 7.78938 3.61751 7.54730

H 8.32217 2.21864 6.58335

H 7.30935 1.98201 8.01539

C 10.49105 3.34492 7.82525

H 11.45083 3.21457 8.33556

H 10.63494 3.07264 6.77488

H 10.21711 4.40276 7.87229

C 9.86828 1.00940 8.49220

H 9.09304 0.36053 8.91221

H 10.10192 0.65317 7.48294

H 10.76711 0.90540 9.10872

P 6.14552 4.69001 9.85541

P 8.98272 6.47424 10.11221

Fe 6.97688 6.56051 9.52042

Fe 8.14165 4.61161 10.47178

96

Cp2Fe2P2, 2S+1=3, dispersion

Fe 11.52855 5.86200 3.56817

Fe 9.13865 6.51879 3.43275

P 10.33882 5.84152 1.77701

P 10.09216 5.66280 5.14886

C 13.43918 6.37346 4.62360

C 13.58240 6.65012 3.21911

C 13.31040 5.41598 2.53126

H 13.35161 5.28422 1.46259

C 13.00940 4.37673 3.45739

C 13.08289 4.98488 4.73727

H 12.91181 4.47006 5.66970

C 7.78588 8.18538 3.95238

C 7.72377 8.01558 2.52384

C 7.33398 6.65347 2.28882

H 7.20732 6.21018 1.31402

C 7.10751 5.97255 3.51247

C 7.39179 6.92660 4.53032

H 7.30587 6.73504 5.58804

C 13.68265 7.19776 5.88894

C 13.10148 8.61406 5.83267

H 13.59579 9.24700 5.09674

H 13.22040 9.09783 6.80784

H 12.03327 8.57555 5.59765

C 13.03998 6.51935 7.11195

H 11.96347 6.37698 6.97438

H 13.19032 7.15247 7.99184

H 13.49258 5.54887 7.33383

C 15.20117 7.24211 6.15194

H 15.59682 6.22703 6.25912

H 15.40446 7.78548 7.08154

H 15.74934 7.72959 5.34566

C 13.90939 7.92081 2.42679

C 12.74926 8.92752 2.46419

H 13.00233 9.81532 1.87289

H 12.50151 9.24915 3.47354

H 11.85423 8.46927 2.04067

C 14.12075 7.56783 0.94379

H 14.93347 6.84718 0.80680

H 14.38144 8.47647 0.39237

H 13.20944 7.16203 0.49385

C 15.20904 8.57872 2.91372

H 16.03392 7.85898 2.92203

H 15.11519 9.00108 3.91414

H 15.48151 9.39765 2.23947

C 12.78319 2.90997 3.16865

C 11.58819 2.38277 3.97239

H 11.72119 2.54302 5.04665

H 11.46138 1.30774 3.80404

H 10.67258 2.89909 3.67050

C 14.05677 2.14961 3.58249

H 14.92835 2.52054 3.03343

H 13.95032 1.07951 3.37337

H 14.25510 2.26994 4.65227

C 12.52003 2.68413 1.67790

H 11.64628 3.25416 1.34569

H 12.33091 1.62285 1.48719

H 13.37840 2.98274 1.06733

C 8.10442 9.37051 4.86517

C 9.25297 10.24794 4.35783

H 9.50569 10.99586 5.11670

H 9.00649 10.78718 3.44404

H 10.14058 9.63593 4.17038

C 6.82503 10.20689 5.05970

H 7.01789 11.03832 5.74690

H 6.02926 9.58951 5.48883

H 6.45514 10.62074 4.12055

C 8.53849 8.87253 6.25622

H 9.40622 8.20914 6.18560

H 7.74012 8.33970 6.77937

H 8.81139 9.73173 6.87687

C 7.92461 8.97142 1.34149

C 9.39655 9.33674 1.10021

H 9.46945 10.07342 0.29190

H 9.95414 8.44486 0.80062

H 9.88214 9.74731 1.98168

C 7.43368 8.30593 0.04312

H 7.54215 9.01388 -0.78445

H 6.37904 8.01867 0.10347

H 8.02818 7.42126 -0.20394

C 7.07911 10.24171 1.52897

H 7.43680 10.86629 2.34715

H 6.03284 9.98769 1.72699

H 7.11469 10.84573 0.61611

C 6.56435 4.57009 3.66863

C 6.64755 4.11384 5.12699

H 6.04409 4.75019 5.78258

H 7.68228 4.13439 5.48441

H 6.27267 3.08966 5.22140

C 7.35337 3.59065 2.79047

H 8.39945 3.55712 3.10768

H 7.33612 3.88930 1.73792

H 6.92642 2.58444 2.86432

C 5.09015 4.58590 3.22399

H 4.64456 3.59158 3.33747

H 4.99985 4.88184 2.17406

H 4.50858 5.29214 3.82545

96

Cp2Fe2P2, 2S+1=5, dispersion

Fe 11.55672 6.05969 3.70556

Fe 9.18819 6.80964 3.52641

P 10.32138 5.85765 1.91893

P 10.40156 6.92736 5.27442

C 13.53696 6.30607 4.64632

C 13.66974 6.62481 3.24033

C 13.28280 5.45423 2.52073

H 13.26358 5.36781 1.44654

C 12.94548 4.39709 3.41674

C 13.08852 4.93337 4.71302

H 12.90113 4.39629 5.63002

C 7.58947 8.24733 3.91015

C 7.65993 8.01358 2.48540

C 7.40054 6.61201 2.29226

H 7.38804 6.11625 1.33536

C 7.13589 5.97247 3.52739

C 7.28169 6.96989 4.51609

H 7.16045 6.81220 5.57654

C 14.02547 6.99759 5.92118

C 13.67730 8.48466 6.03647

H 14.14478 9.09352 5.26397

H 14.02520 8.86373 7.00308

H 12.59369 8.63142 5.98760

C 13.43639 6.31541 7.16892

H 12.34277 6.32011 7.14785

H 13.76046 6.85919 8.06158

H 13.78335 5.28418 7.27740

C 15.55501 6.80791 5.97950

H 15.80686 5.74281 5.95758

H 15.95173 7.23315 6.90827

H 16.06100 7.28754 5.14034

C 13.96102 7.93425 2.50156

C 12.85949 8.97351 2.77737

H 13.05946 9.89409 2.21692

H 12.77170 9.22820 3.83167

H 11.89264 8.58007 2.45065

C 13.94695 7.69032 0.98278

H 14.70649 6.96248 0.67922

H 14.16115 8.63102 0.46609

H 12.96618 7.34197 0.64401

C 15.34723 8.49610 2.84954

H 16.12624 7.75131 2.65662

H 15.42883 8.80766 3.89089

H 15.55704 9.37352 2.22824

C 12.57372 2.96997 3.07494

C 11.25946 2.58802 3.76953

H 11.32382 2.72320 4.85402

H 11.01096 1.53916 3.57367

H 10.44471 3.21525 3.39549

C 13.70526 2.05332 3.57215

H 14.65820 2.32025 3.10381

H 13.48511 1.00785 3.32913

H 13.82876 2.13166 4.65692

C 12.40872 2.79650 1.56312

H 11.62506 3.45693 1.17747

H 12.12724 1.76357 1.33411

H 13.34120 3.01352 1.03143

C 7.58037 9.51426 4.76805

C 8.68928 10.51160 4.41847

H 8.67145 11.34536 5.12824

H 8.58022 10.93455 3.42041

H 9.67009 10.02874 4.48390

C 6.19427 10.17708 4.63594

H 6.13662 11.05895 5.28386

H 5.41043 9.47799 4.94476

H 5.97733 10.49021 3.61497

C 7.76051 9.16719 6.25632

H 8.68906 8.61636 6.43290

H 6.92602 8.57759 6.64631

H 7.79825 10.09383 6.83748

C 7.95871 8.92314 1.28585

C 9.42316 9.39271 1.26574

H 9.58077 10.09713 0.44079

H 10.08172 8.53238 1.11356

H 9.72363 9.88148 2.19099

C 7.74122 8.14564 -0.02374

H 7.94334 8.80811 -0.87122

H 6.71189 7.78456 -0.11848

H 8.42572 7.29546 -0.10171

C 7.01027 10.13108 1.24556

H 7.19771 10.83821 2.05381

H 5.96497 9.81118 1.30538

H 7.14524 10.67202 0.30276

C 6.83063 4.51002 3.76446

C 7.67375 3.98054 4.93389

H 7.47072 4.52959 5.85847

H 8.74114 4.07825 4.71399

H 7.45211 2.92326 5.11510

C 7.14100 3.68430 2.51248

H 8.18690 3.80930 2.21247

H 6.50518 3.97790 1.67071

H 6.96072 2.62250 2.70878

C 5.33719 4.38015 4.11108

H 5.07375 3.33119 4.28718

H 4.71342 4.75734 3.29435

H 5.09289 4.94969 5.01356

98

Cp2Fe2As2CO, 2S+1=1 - open-shell singlet, without dispersion

C 7.02078 2.70248 8.98042

C 6.83941 4.10759 9.33515

C 6.20664 4.72418 8.20395

H 5.95068 5.77121 8.14283

C 5.96756 3.78025 7.17305

C 6.49405 2.55538 7.65244

H 6.49633 1.63039 7.09534

C 7.52136 1.47424 9.76679

C 6.71577 1.29513 11.06879

H 6.88424 2.09457 11.79084

H 7.00176 0.35390 11.55173

H 5.64197 1.25046 10.85787

C 9.03122 1.53342 10.06967

H 9.60328 1.49526 9.13805

H 9.32498 0.67090 10.67985

H 9.32339 2.43839 10.60062

C 7.29352 0.18621 8.94891

H 6.23239 0.02388 8.73138

H 7.64535 -0.67170 9.53175

H 7.84879 0.19299 8.00723

C 6.98602 4.91677 10.63869

C 7.13633 6.42155 10.32765

H 7.99798 6.61351 9.68092

H 7.28662 6.96907 11.26401

H 6.24560 6.84378 9.85389

C 8.19249 4.54524 11.51162

H 8.14834 3.52720 11.89872

H 8.23240 5.21577 12.37730

H 9.12608 4.66494 10.95299

C 5.68375 4.74779 11.45327

H 4.81993 5.09224 10.87499

H 5.73339 5.34207 12.37340

H 5.50294 3.70753 11.73260

C 5.18139 3.98349 5.88907

C 5.87604 3.29494 4.70493

H 6.86141 3.73694 4.52768

H 5.27745 3.40826 3.79409

H 6.01338 2.22328 4.88033

C 3.78574 3.35448 6.09183

H 3.86105 2.28140 6.29527

H 3.17230 3.48806 5.19333

H 3.26279 3.82106 6.93323

C 5.01158 5.47466 5.57010

H 4.45227 5.99564 6.35438

H 4.45490 5.59595 4.63472

H 5.98235 5.96798 5.45525

C 12.67860 4.62662 5.52952

C 12.59113 3.18455 5.72478

C 11.65621 2.70490 4.75032

H 11.35237 1.67430 4.65287

C 11.17038 3.76318 3.93951

C 11.79268 4.93710 4.44371

H 11.63694 5.93000 4.04864

C 13.60460 5.73505 6.06644

C 14.01708 5.60605 7.53880

H 13.13960 5.59574 8.19265

H 14.63024 6.47041 7.81683

H 14.61252 4.71540 7.74004

C 12.92575 7.11569 5.93582

H 12.75572 7.40510 4.89493

H 13.57638 7.87976 6.37401

H 11.96741 7.14089 6.46312

C 14.87170 5.75591 5.18178

H 15.42853 4.81747 5.24115

H 15.53888 6.56691 5.49682

H 14.60880 5.92258 4.13203

C 13.33708 2.19330 6.63972

C 13.02678 2.38898 8.13626

H 13.27970 3.38321 8.50043

H 13.59096 1.65952 8.72969

H 11.96149 2.22850 8.32866

C 12.92622 0.74465 6.30520

H 11.85835 0.57205 6.46856

H 13.47417 0.05895 6.95982

H 13.16890 0.47722 5.27142

C 14.85645 2.27751 6.39083

H 15.08659 2.12239 5.33136

H 15.36970 1.49711 6.96407

H 15.28285 3.23600 6.68969

C 10.34894 3.64567 2.66590

C 9.46485 4.88339 2.45573

H 8.75829 5.00732 3.28202

H 8.89500 4.78638 1.52505

H 10.05903 5.80006 2.38235

C 11.34189 3.53786 1.48738

H 11.98269 4.42371 1.42933

H 10.80061 3.44621 0.53844

H 11.98797 2.66051 1.59618

C 9.46409 2.39199 2.68143

H 10.05742 1.47611 2.76885

H 8.89568 2.32498 1.74739

H 8.75280 2.41441 3.51112

As 8.96743 5.90650 6.34854

As 10.20185 5.14834 8.20770

Fe 8.21732 3.97481 7.56552

Fe 10.52944 4.11162 6.07214

O 9.15108 1.47475 6.23944

C 9.24204 2.62372 6.48493

98

Cp2Fe2As2CO, 2S+1 = 1, without dispersion

As 9.03249 5.93293 6.47740

As 10.20541 5.07649 8.21453

Fe 8.32474 3.84512 7.37074

Fe 10.35897 3.97476 6.11071

O 8.99842 1.43392 5.93084

C 7.30516 2.69680 8.85652

C 7.15625 4.11504 9.14398

C 6.52774 4.69477 7.98340

H 6.28659 5.74236 7.88253

C 6.24858 3.70716 7.00487

C 6.75500 2.49653 7.54641

H 6.74296 1.54808 7.03220

C 7.80494 1.50046 9.68820

C 6.97017 1.34611 10.97519

H 7.12205 2.15905 11.68585

H 7.24745 0.41462 11.48138

H 5.90094 1.29377 10.74310

C 9.30378 1.59222 10.01940

H 9.88895 1.51141 9.10007

H 9.59174 0.76571 10.67992

H 9.57969 2.52750 10.50459

C 7.61466 0.18596 8.90251

H 6.55860 -0.01481 8.69135

H 7.99061 -0.64543 9.50832

H 8.16710 0.18628 7.95929

C 7.24853 4.96031 10.43091

C 7.37340 6.46433 10.10624

H 8.25509 6.67738 9.49579

H 7.46520 7.02378 11.04297

H 6.49210 6.85555 9.58994

C 8.40743 4.62552 11.37938

H 8.36240 3.61072 11.77409

H 8.37565 5.30609 12.23740

H 9.37274 4.76305 10.88260

C 5.90466 4.77608 11.17543

H 5.06539 5.08410 10.54288

H 5.89147 5.39749 12.07831

H 5.73372 3.74026 11.47557

C 5.40091 3.86516 5.75451

C 5.83213 2.88218 4.65862

H 6.86740 3.05852 4.35752

H 5.19296 2.99981 3.77680

H 5.75061 1.84164 4.98788

C 3.94309 3.55275 6.15877

H 3.84822 2.52977 6.53767

H 3.27832 3.65783 5.29361

H 3.59390 4.23639 6.93977

C 5.46472 5.29721 5.20674

H 5.08485 6.02641 5.93010

H 4.84772 5.38040 4.30545

H 6.48920 5.58029 4.94863

C 12.37205 4.63267 5.75522

C 12.32329 3.18331 5.88714

C 11.43656 2.72097 4.85781

H 11.16374 1.68850 4.70548

C 10.93709 3.80098 4.08027

C 11.49071 4.96544 4.66595

H 11.30330 5.97182 4.32123

C 13.32246 5.72928 6.27942

C 13.81950 5.57625 7.72154

H 12.98369 5.55885 8.42728

H 14.45072 6.43526 7.97386

H 14.42353 4.68133 7.87097

C 12.67274 7.12642 6.19092

H 12.44738 7.42346 5.16277

H 13.37245 7.86940 6.58765

H 11.75094 7.18013 6.77570

C 14.54100 5.73273 5.32545

H 15.08056 4.78282 5.34365

H 15.23895 6.52560 5.61790

H 14.22944 5.92143 4.29296

C 13.08113 2.17877 6.77569

C 12.83363 2.38013 8.27980

H 13.12733 3.36421 8.63905

H 13.39917 1.63253 8.84858

H 11.77326 2.25086 8.50688

C 12.62497 0.74009 6.45656

H 11.55172 0.60494 6.61807

H 13.15176 0.04548 7.11901

H 12.86134 0.45109 5.42699

C 14.59123 2.23445 6.46829

H 14.77917 2.09772 5.39789

H 15.10662 1.42969 7.00465

H 15.05045 3.17603 6.77413

C 10.15420 3.73720 2.78025

C 9.07771 4.83025 2.72407

H 8.34100 4.69600 3.52011

H 8.55710 4.79785 1.76043

H 9.50832 5.83101 2.83358

C 11.16180 3.97068 1.63350

H 11.63027 4.95743 1.71027

H 10.65345 3.91115 0.66430

H 11.95617 3.21697 1.64664

C 9.50194 2.36163 2.58676

H 10.25387 1.56817 2.51832

H 8.92784 2.34983 1.65402

H 8.82742 2.11230 3.40932

C 9.15326 2.55509 6.29858

98

Cp2Fe2As2CO, 2S+1 = 3, , without dispersion

As 8.96457 5.89992 6.46495

As 10.14565 5.03565 8.25549

Fe 8.26883 3.87196 7.42481

Fe 10.38305 4.02247 6.11705

O 9.03118 1.44676 6.03385

C 7.24546 2.71701 8.92833

C 7.07664 4.13253 9.20501

C 6.43828 4.69956 8.03871

H 6.18040 5.74305 7.93695

C 6.17376 3.70770 7.06289

C 6.70488 2.50832 7.61140

H 6.69927 1.55449 7.10588

C 7.73961 1.52675 9.77305

C 6.88612 1.37423 11.04784

H 7.01175 2.19979 11.74933

H 7.16771 0.45429 11.57277

H 5.82215 1.30327 10.79725

C 9.23340 1.62333 10.12893

H 9.83509 1.59233 9.21673

H 9.52250 0.77274 10.75785

H 9.48914 2.53857 10.66064

C 7.56663 0.20758 8.99081

H 6.51534 0.00449 8.75942

H 7.93096 -0.62061 9.60804

H 8.13760 0.20223 8.05856

C 7.16701 4.99543 10.47961

C 7.31806 6.49193 10.13189

H 8.21151 6.68074 9.52963

H 7.40836 7.06665 11.05956

H 6.45103 6.88941 9.59696

C 8.31396 4.66212 11.44262

H 8.25071 3.65510 11.85433

H 8.28570 5.35769 12.28875

H 9.28485 4.77645 10.95075

C 5.81649 4.83883 11.21808

H 4.98444 5.14124 10.57344

H 5.80099 5.47608 12.10992

H 5.63586 3.80925 11.53507

C 5.33757 3.84359 5.80233

C 5.90166 2.97465 4.67054

H 6.91569 3.29093 4.41226

H 5.27318 3.06120 3.77725

H 5.94020 1.91630 4.94662

C 3.90901 3.36516 6.14013

H 3.90869 2.31828 6.46125

H 3.25856 3.45131 5.26198

H 3.47253 3.96515 6.94571

C 5.27326 5.30196 5.32963

H 4.80171 5.95039 6.07575

H 4.67763 5.37244 4.41300

H 6.27295 5.69502 5.11980

C 12.44130 4.61868 5.70881

C 12.42351 3.16515 5.78637

C 11.52158 2.72397 4.76889

H 11.24583 1.69369 4.60442

C 11.01525 3.81462 4.01067

C 11.55679 4.97298 4.61500

H 11.38620 5.98436 4.27755

C 13.37539 5.71174 6.26438

C 13.85118 5.52490 7.70988

H 13.00156 5.48245 8.39802

H 14.47088 6.38170 7.99597

H 14.45963 4.63105 7.84770

C 12.69883 7.09815 6.21058

H 12.48761 7.42635 5.18886

H 13.37466 7.84095 6.64711

H 11.76434 7.11081 6.77762

C 14.60421 5.76546 5.32620

H 15.16640 4.82849 5.32878

H 15.27995 6.56795 5.64357

H 14.29949 5.97030 4.29464

C 13.18460 2.14277 6.65202

C 12.89737 2.28547 8.15734

H 13.16374 3.26346 8.55315

H 13.46456 1.53026 8.71417

H 11.83441 2.12706 8.35879

C 12.76881 0.70780 6.26914

H 11.69943 0.53666 6.42281

H 13.31261 -0.00078 6.90233

H 13.01456 0.47197 5.22835

C 14.70002 2.24202 6.38346

H 14.91578 2.13405 5.31506

H 15.22189 1.43770 6.91411

H 15.12976 3.18695 6.71960

C 10.22100 3.74895 2.71656

C 9.21702 4.90664 2.61674

H 8.48004 4.86325 3.42354

H 8.68426 4.86112 1.66049

H 9.71384 5.88080 2.67077

C 11.23323 3.86893 1.55579

H 11.76850 4.82344 1.59369

H 10.71516 3.80797 0.59173

H 11.97466 3.06398 1.59418

C 9.47756 2.41263 2.58423

H 10.17268 1.56762 2.54040

H 8.89320 2.40239 1.65797

H 8.79607 2.24265 3.42143

C 9.10007 2.57284 6.40350

98

Cp2Fe2As2CO 2S+1 = 5, , without dispersion

As 8.93698 5.92057 6.26361

As 10.11586 5.26878 8.11451

Fe 8.25318 3.90147 7.41951

Fe 10.41720 4.02544 6.05039

O 9.09669 1.44915 6.12463

C 7.12584 2.69057 8.89246

C 6.93755 4.08839 9.28232

C 6.26993 4.71865 8.18847

H 6.02039 5.76822 8.14599

C 6.01002 3.79709 7.14526

C 6.55326 2.56059 7.58119

H 6.53178 1.64326 7.01239

C 7.63897 1.44895 9.65113

C 6.86439 1.25535 10.96978

H 7.06403 2.03710 11.70303

H 7.15116 0.30078 11.42514

H 5.78478 1.22789 10.78752

C 9.15446 1.49853 9.92176

H 9.70343 1.45226 8.97732

H 9.45551 0.63523 10.52716

H 9.46471 2.40268 10.44471

C 7.38650 0.17248 8.82244

H 6.31866 0.01290 8.63753

H 7.75579 -0.69298 9.38308

H 7.91192 0.19472 7.86443

C 7.10724 4.86796 10.60087

C 7.21304 6.38326 10.32352

H 8.04321 6.61198 9.64862

H 7.38856 6.91017 11.26733

H 6.29487 6.79648 9.89622

C 8.34607 4.50019 11.42954

H 8.33215 3.47237 11.79235

H 8.39851 5.15066 12.30963

H 9.26127 4.65073 10.84851

C 5.83382 4.65539 11.45089

H 4.94596 4.98985 10.90422

H 5.89927 5.23737 12.37799

H 5.68311 3.60813 11.72027

C 5.18141 4.02142 5.89122

C 5.82473 3.33915 4.67482

H 6.80487 3.77598 4.45967

H 5.19080 3.46465 3.78993

H 5.96307 2.26540 4.83481

C 3.79006 3.39875 6.14001

H 3.86591 2.32303 6.32881

H 3.14459 3.54599 5.26642

H 3.30201 3.85944 7.00523

C 5.00905 5.51676 5.59412

H 4.48509 6.03335 6.40519

H 4.41636 5.65084 4.68286

H 5.97739 6.00573 5.44508

C 12.57700 4.65298 5.58299

C 12.50970 3.21512 5.80497

C 11.61180 2.69480 4.81482

H 11.33603 1.65616 4.72140

C 11.12193 3.73306 3.97909

C 11.70559 4.92699 4.48045

H 11.53029 5.91024 4.06986

C 13.48923 5.78128 6.10282

C 13.88325 5.69833 7.58386

H 12.99850 5.69831 8.22735

H 14.48408 6.57661 7.84450

H 14.48521 4.82009 7.81803

C 12.81160 7.15614 5.91765

H 12.65621 7.40984 4.86524

H 13.45725 7.93430 6.33800

H 11.84684 7.20090 6.43094

C 14.76955 5.77818 5.23663

H 15.32824 4.84390 5.33217

H 15.42952 6.60040 5.53787

H 14.52241 5.91323 4.17849

C 13.24552 2.25647 6.76213

C 12.90405 2.49584 8.24463

H 13.15099 3.49926 8.58689

H 13.45359 1.78272 8.87052

H 11.83472 2.34116 8.41641

C 12.84908 0.79587 6.46380

H 11.77736 0.62421 6.60099

H 13.38116 0.13464 7.15560

H 13.12320 0.49519 5.44711

C 14.76856 2.34475 6.53931

H 15.02066 2.16104 5.48943

H 15.27538 1.58440 7.14427

H 15.18242 3.31440 6.81905

C 10.33613 3.58220 2.68598

C 9.41504 4.78716 2.44718

H 8.67932 4.88770 3.25071

H 8.87655 4.66771 1.50063

H 9.97738 5.72474 2.38885

C 11.36526 3.50850 1.53602

H 11.97275 4.41822 1.48960

H 10.85346 3.39126 0.57373

H 12.04131 2.65711 1.66692

C 9.49908 2.29619 2.68070

H 10.12549 1.40396 2.78030

H 8.95573 2.21033 1.73342

H 8.77062 2.28664 3.49505

C 9.20663 2.60236 6.39951

98

Cp2Fe2P2CO 2S+1 = 1 - open-shell singlet, without dispersion

C 7.02985 2.68380 8.98925

C 6.84039 4.08831 9.33623

C 6.21369 4.69849 8.19698

H 5.95501 5.74450 8.13055

C 5.98384 3.74981 7.16903

C 6.51352 2.52956 7.65750

H 6.52189 1.60171 7.10518

C 7.54099 1.46191 9.77933

C 6.74782 1.28340 11.08869

H 6.92030 2.08587 11.80638

H 7.04267 0.34509 11.57193

H 5.67228 1.23330 10.88827

C 9.05264 1.53218 10.07125

H 9.61830 1.49935 9.13547

H 9.35799 0.67152 10.67835

H 9.34157 2.43897 10.60091

C 7.31507 0.16880 8.96893

H 6.25381 0.00180 8.75555

H 7.67203 -0.68492 9.55476

H 7.86709 0.17227 8.02530

C 6.98068 4.90247 10.63682

C 7.09920 6.40927 10.32209

H 7.94838 6.61943 9.66485

H 7.25019 6.96065 11.25604

H 6.19399 6.81288 9.85953

C 8.20163 4.55244 11.49868

H 8.17313 3.53630 11.89265

H 8.24227 5.22814 12.36036

H 9.12779 4.67918 10.92968

C 5.68823 4.71354 11.46258

H 4.81387 5.04015 10.88977

H 5.73471 5.31286 12.37960

H 5.52777 3.67185 11.74809

C 5.20133 3.94412 5.88146

C 5.89529 3.24197 4.70512

H 6.88386 3.67597 4.52698

H 5.30040 3.35202 3.79148

H 6.02486 2.17071 4.88883

C 3.80268 3.32250 6.08615

H 3.87330 2.25098 6.29951

H 3.19193 3.45020 5.18491

H 3.27942 3.79861 6.92199

C 5.03870 5.43331 5.54926

H 4.47727 5.96215 6.32673

H 4.48586 5.54858 4.61080

H 6.01093 5.92329 5.43474

C 12.66194 4.65166 5.52673

C 12.58882 3.20905 5.71729

C 11.65126 2.72535 4.74698

H 11.35822 1.69202 4.64503

C 11.15340 3.78246 3.94031

C 11.76645 4.95893 4.44687

H 11.59842 5.95250 4.05863

C 13.58042 5.76421 6.06702

C 13.97101 5.64253 7.54628

H 13.08432 5.62352 8.18713

H 14.57206 6.51282 7.83226

H 14.57230 4.75804 7.75754

C 12.90381 7.14371 5.91651

H 12.75176 7.42551 4.87076

H 13.54749 7.91053 6.35999

H 11.93709 7.17489 6.42754

C 14.85916 5.78289 5.19908

H 15.41796 4.84675 5.27027

H 15.51988 6.59752 5.51845

H 14.60935 5.94361 4.14514

C 13.34068 2.22245 6.63261

C 13.01308 2.40880 8.12676

H 13.24803 3.40577 8.49525

H 13.58163 1.68593 8.72408

H 11.94837 2.23293 8.30885

C 12.95133 0.77074 6.28656

H 11.88502 0.58259 6.44267

H 13.50457 0.08804 6.93987

H 13.20315 0.51386 5.25229

C 14.86136 2.32592 6.40063

H 15.10509 2.18111 5.34273

H 15.37729 1.54734 6.97395

H 15.27359 3.28677 6.71135

C 10.33162 3.66289 2.66708

C 9.41398 4.87960 2.47867

H 8.70031 4.96754 3.30313

H 8.85083 4.78603 1.54352

H 9.98291 5.81350 2.42672

C 11.32374 3.60084 1.48434

H 11.93781 4.50602 1.43651

H 10.78243 3.50614 0.53567

H 11.99611 2.74173 1.57896

C 9.48296 2.38443 2.66438

H 10.10368 1.48492 2.72937

H 8.90970 2.31885 1.73326

H 8.77748 2.36946 3.49923

P 9.01466 5.81981 6.45321

P 10.14306 5.12362 8.12054

Fe 8.23051 3.95863 7.57524

Fe 10.52006 4.11457 6.08105

O 9.16315 1.45618 6.23147

C 9.24939 2.60323 6.48151

98

Cp2Fe2P2CO, 2S+1 = 1, without dispersion

Fe 3.37705 3.98243 6.16060

Fe 1.35748 3.86784 7.41218

P 2.08261 5.84568 6.53479

P 3.15908 5.12087 8.13059

O 2.03830 1.42080 6.02924

C 2.18590 2.54982 6.37246

C 3.95975 3.78666 4.13236

C 4.49306 4.96757 4.70175

H 4.28183 5.96633 4.34908

C 5.37845 4.66416 5.79710

C 5.35411 3.21754 5.95037

C 4.47341 2.72596 4.92818

H 4.21820 1.68710 4.78947

C -0.72323 3.75375 7.06531

C -0.22724 2.54057 7.61186

H -0.25718 1.58795 7.10618

C 0.33260 2.74342 8.91835

C 0.20706 4.16486 9.19420

C -0.42152 4.74454 8.03327

H -0.64007 5.79600 7.92229

C 3.17002 3.69087 2.83831

C 2.53939 2.30210 2.66900

H 1.86975 2.05650 3.49680

H 3.30335 1.51930 2.61211

H 1.96387 2.26600 1.73777

C 2.07362 4.76350 2.77580

H 1.34646 4.62577 3.57983

H 1.54606 4.70790 1.81703

H 2.48590 5.77345 2.86858

C 4.16557 3.92539 1.68141

H 3.65134 3.84495 0.71685

H 4.97219 3.18484 1.69836

H 4.61845 4.92049 1.74191

C 6.30217 5.78389 6.31763

C 7.53561 5.79433 5.38299

H 8.09170 4.85487 5.42426

H 8.21454 6.60412 5.67390

H 7.23702 5.96184 4.34297

C 5.63120 7.16820 6.19455

H 5.41903 7.44362 5.15771

H 6.31252 7.92903 6.58942

H 4.69948 7.21958 6.76325

C 6.77692 5.66115 7.77044

H 5.93091 5.63370 8.46330

H 7.38541 6.53708 8.02074

H 7.39911 4.78245 7.94099

C 6.12224 2.23900 6.85890

C 5.70003 0.78787 6.54986

H 4.62861 0.63088 6.70421

H 6.23616 0.11098 7.22307

H 5.95099 0.49472 5.52490

C 5.84969 2.45326 8.35715

H 6.12078 3.44652 8.70856

H 6.42037 1.72247 8.94243

H 4.78875 2.30823 8.57081

C 7.63479 2.32281 6.57105

H 7.84075 2.17823 5.50499

H 8.15932 1.53505 7.12354

H 8.06988 3.27715 6.87247

C -1.57474 3.91396 5.81806

C -3.03898 3.65859 6.23919

H -3.16365 2.64902 6.64459

H -3.70612 3.76283 5.37575

H -3.36096 4.37257 7.00457

C -1.46699 5.33102 5.23919

H -1.80800 6.08848 5.95267

H -2.09547 5.41806 4.34618

H -0.43747 5.57113 4.95980

C -1.18519 2.89276 4.74146

H -0.14377 3.01933 4.43593

H -1.82003 3.01930 3.85780

H -1.31067 1.86329 5.09148

C 0.82695 1.54716 9.75380

C 2.33600 1.61437 10.04242

H 2.89355 1.49543 9.11024

H 2.62508 0.80073 10.71825

H 2.64325 2.55798 10.49129

C 0.58798 0.22641 8.99215

H -0.47691 0.04965 8.80514

H 0.95567 -0.60499 9.60301

H 1.12219 0.19862 8.03900

C 0.02598 1.42767 11.06553

H 0.21940 2.24430 11.76139

H 0.29561 0.49538 11.57431

H -1.05067 1.39889 10.86579

C 0.33956 5.02029 10.47017

C 1.52454 4.68375 11.38526

H 1.47897 3.67502 11.79551

H 1.52863 5.37548 12.23502

H 2.47462 4.80120 10.85605

C 0.46384 6.52051 10.12835

H 0.58244 7.08761 11.05747

H -0.42933 6.91084 9.63212

H 1.33072 6.72683 9.49498

C -0.98397 4.85501 11.25444

H -1.15709 3.82381 11.56798

H -1.83804 5.16683 10.64379

H -0.96512 5.48363 12.15227

98

Cp2Fe2P2CO 2S+1 = 3, without dispersion

Fe 3.40481 4.03463 6.16946

Fe 1.30548 3.89754 7.46846

P 2.02221 5.82169 6.53522

P 3.09722 5.09443 8.17797

O 2.06629 1.44254 6.12231

C 2.13218 2.57374 6.47293

C 4.03405 3.81028 4.06152

C 4.56275 4.97915 4.65346

H 4.37460 5.98584 4.31161

C 5.45248 4.64585 5.75097

C 5.45049 3.19473 5.84495

C 4.54788 2.73347 4.83585

H 4.28246 1.69865 4.68299

C -0.79389 3.74677 7.12356

C -0.26854 2.54956 7.68305

H -0.28728 1.58922 7.19026

C 0.27828 2.76857 8.99703

C 0.12541 4.18672 9.25638

C -0.51084 4.74703 8.08466

H -0.74938 5.79361 7.96831

C 3.23276 3.72283 2.77303

C 2.50418 2.37678 2.65772

H 1.82662 2.20821 3.49848

H 3.20872 1.53924 2.62098

H 1.91730 2.34987 1.73342

C 2.21443 4.86788 2.67097

H 1.48594 4.82460 3.48533

H 1.67319 4.80619 1.72040

H 2.70039 5.84812 2.71004

C 4.23556 3.84409 1.60436

H 3.71150 3.76929 0.64452

H 4.98612 3.04769 1.64407

H 4.76038 4.80475 1.63054

C 6.37019 5.75471 6.30094

C 7.61288 5.80375 5.38095

H 8.18380 4.87268 5.40828

H 8.27602 6.61816 5.69461

H 7.32192 5.98816 4.34154

C 5.68438 7.13463 6.21094

H 5.49506 7.44485 5.17936

H 6.34468 7.88928 6.65074

H 4.73741 7.15147 6.75636

C 6.82373 5.59406 7.75699

H 5.96495 5.54923 8.43314

H 7.42864 6.46221 8.04069

H 7.44200 4.71032 7.91570

C 6.21499 2.19056 6.72860

C 5.82305 0.74695 6.35351

H 4.75502 0.56140 6.49996

H 6.37163 0.05109 6.99657

H 6.08054 0.50710 5.31644

C 5.90422 2.34547 8.22820

H 6.15230 3.33102 8.61688

H 6.47250 1.60355 8.80153

H 4.84057 2.17598 8.41626

C 7.73244 2.30680 6.48017

H 7.96489 2.19185 5.41599

H 8.25690 1.51412 7.02556

H 8.14541 3.26003 6.81375

C -1.63486 3.87614 5.86561

C -3.07128 3.43564 6.22136

H -3.09222 2.39569 6.56375

H -3.72548 3.51823 5.34564

H -3.48933 4.06112 7.01727

C -1.67006 5.32608 5.36469

H -2.11866 5.99959 6.10265

H -2.27441 5.39218 4.45339

H -0.66438 5.69181 5.13645

C -1.09733 2.97279 4.74778

H -0.07756 3.25989 4.47821

H -1.72825 3.05759 3.85608

H -1.08312 1.91903 5.04342

C 0.77303 1.58423 9.85008

C 2.27606 1.66830 10.16864

H 2.85461 1.60427 9.24344

H 2.56918 0.83112 10.81350

H 2.55636 2.59508 10.66680

C 0.56464 0.25542 9.09303

H -0.49377 0.06632 8.88313

H 0.92675 -0.56843 9.71734

H 1.11972 0.22531 8.15177

C -0.05089 1.46261 11.14723

H 0.10759 2.29473 11.83383

H 0.22871 0.54483 11.67693

H -1.12200 1.40662 10.92489

C 0.24362 5.06350 10.51841

C 1.41514 4.73914 11.45465

H 1.35618 3.73965 11.88533

H 1.41451 5.44838 12.28994

H 2.37228 4.83750 10.93426

C 0.38284 6.55621 10.15069

H 0.49421 7.14086 11.06985

H -0.49919 6.94476 9.63398

H 1.26056 6.74328 9.52563

C -1.08910 4.91675 11.29053

H -1.26422 3.89159 11.62368

H -1.93583 5.21351 10.66246

H -1.08253 5.56434 12.17506

98

Cp2Fe2P2CO, 2S+1 = 5, without dispersion

Fe 3.43470 4.03801 6.09474

Fe 1.29275 3.92182 7.46751

P 1.99309 5.84415 6.34370

P 3.07338 5.31216 8.02643

O 2.14393 1.44230 6.21838

C 2.24565 2.60066 6.47040

C 4.14205 3.73604 4.02243

C 4.71157 4.93898 4.51453

H 4.52391 5.91744 4.09821

C 5.58424 4.68296 5.62194

C 5.53018 3.24772 5.85740

C 4.63736 2.71006 4.87115

H 4.37258 1.66777 4.78590

C -0.95543 3.83031 7.21231

C -0.41308 2.59676 7.65849

H -0.44649 1.67158 7.10308

C 0.16756 2.73927 8.96635

C -0.00967 4.14177 9.33852

C -0.68274 4.76229 8.24177

H -0.92616 5.81285 8.18888

C 3.35415 3.56502 2.73308

C 2.53221 2.26918 2.74221

H 1.80888 2.25659 3.56126

H 3.16981 1.38510 2.84372

H 1.98376 2.17014 1.79919

C 2.41825 4.75713 2.48748

H 1.68131 4.85413 3.29014

H 1.88090 4.62471 1.54199

H 2.96906 5.70099 2.42222

C 4.38010 3.49288 1.58027

H 3.86655 3.36011 0.62093

H 5.06719 2.65123 1.71680

H 4.97604 4.40958 1.52305

C 6.48575 5.82246 6.13470

C 7.78047 5.80664 5.28984

H 8.34139 4.87710 5.41285

H 8.43224 6.63747 5.58525

H 7.55035 5.91955 4.22523

C 5.81032 7.19209 5.90912

H 5.67819 7.42435 4.84865

H 6.44590 7.97924 6.32792

H 4.83479 7.24760 6.40032

C 6.85454 5.76767 7.62387

H 5.96027 5.77004 8.25377

H 7.44461 6.65477 7.87930

H 7.46035 4.89876 7.88219

C 6.26199 2.30784 6.83574

C 5.88665 0.83971 6.54782

H 4.81550 0.65608 6.67309

H 6.41748 0.19200 7.25326

H 6.17764 0.53227 5.53782

C 5.88909 2.56466 8.30765

H 6.11881 3.57591 8.63853

H 6.43210 1.86650 8.95568

H 4.81779 2.40336 8.45992

C 7.78767 2.40923 6.64038

H 8.06163 2.21437 5.59796

H 8.29059 1.66215 7.26478

H 8.18642 3.38649 6.91510

C -1.79120 4.04559 5.96133

C -3.18829 3.44453 6.23028

H -3.12441 2.37081 6.43448

H -3.83910 3.58690 5.35980

H -3.66352 3.92403 7.09241

C -1.94644 5.53869 5.64355

H -2.46055 6.07253 6.44976

H -2.54254 5.66700 4.73367

H -0.97354 6.01435 5.48332

C -1.16755 3.33644 4.74997

H -0.18320 3.75516 4.51898

H -1.80728 3.45816 3.86874

H -1.04393 2.26309 4.92424

C 0.67607 1.50258 9.73682

C 2.19493 1.53780 9.98920

H 2.73183 1.48041 9.03861

H 2.49414 0.67447 10.59547

H 2.52099 2.44076 10.50445

C 0.39974 0.21792 8.92847

H -0.67163 0.06979 8.75504

H 0.76309 -0.64439 9.49776

H 0.91738 0.22041 7.96598

C -0.08342 1.33663 11.06764

H 0.13839 2.12527 11.78704

H 0.19628 0.38421 11.53185

H -1.16584 1.32256 10.90120

C 0.18666 4.94022 10.64147

C 1.44008 4.57784 11.45064

H 1.42258 3.55767 11.83483

H 1.51607 5.24457 12.31676

H 2.34503 4.70701 10.84919

C 0.29234 6.45033 10.33739

H 0.49916 6.99135 11.26672

H -0.63680 6.86123 9.93198

H 1.10243 6.66500 9.63416

C -1.07054 4.74812 11.52002

H -1.21955 3.70680 11.81206

H -1.96787 5.07584 10.98484

H -0.98468 5.34699 12.43458

96

Cp2Fe2N2, 2S+1 = 1 open-shell, with dispersion

C 5.55047 7.25712 8.32607

C 6.85557 7.81694 7.96116

C 7.32652 8.52842 9.10323

H 8.29504 8.99366 9.18013

C 6.43035 8.36731 10.18669

C 5.32377 7.62727 9.67743

H 4.48608 7.30033 10.27185

C 4.48535 6.47470 7.55801

C 3.54409 5.76339 8.54799

H 4.11419 5.11561 9.22033

H 2.83042 5.15221 7.98648

H 2.95854 6.47241 9.14125

C 3.64039 7.47407 6.74580

H 3.18649 8.21636 7.41033

H 2.83368 6.94524 6.22605

H 4.23041 8.00998 6.00064

C 5.04772 5.37923 6.64723

H 5.63704 5.77268 5.81975

H 4.21935 4.81096 6.21139

H 5.66723 4.69012 7.22788

C 7.68426 7.78052 6.67264

C 6.85384 8.24299 5.46635

H 7.50112 8.33393 4.58767

H 6.40175 9.22206 5.65562

H 6.05671 7.54445 5.21319

C 8.29062 6.39176 6.42091

H 8.94168 6.12285 7.25554

H 8.88386 6.40569 5.49946

H 7.53639 5.61386 6.32644

C 8.87234 8.75137 6.79284

H 8.54300 9.77806 6.98268

H 9.43372 8.74691 5.85341

H 9.55903 8.44664 7.58776

C 6.57918 8.92525 11.58206

C 8.06195 9.07559 11.93972

H 8.58531 8.12184 11.82817

H 8.16603 9.41983 12.97379

H 8.55797 9.80939 11.29652

C 5.90747 7.99235 12.59528

H 4.82756 7.92464 12.43070

H 6.06446 8.36337 13.61326

H 6.32207 6.98444 12.51998

C 5.89535 10.30321 11.61523

H 6.35977 10.98744 10.89792

H 5.97818 10.74600 12.61387

H 4.83272 10.21957 11.36533

C 9.25239 3.91624 11.93228

C 7.99463 3.16547 11.83945

C 7.97091 2.58759 10.54284

H 7.14075 2.03871 10.12903

C 9.12712 2.96688 9.80594

C 9.93011 3.72530 10.69512

H 10.85345 4.20823 10.42060

C 9.93633 4.70145 13.05121

C 10.96074 5.68179 12.44893

H 10.49302 6.32292 11.69558

H 11.37095 6.30891 13.24666

H 11.80511 5.16196 11.98681

C 10.70385 3.70334 13.93887

H 11.42865 3.14062 13.34196

H 11.25150 4.24195 14.72023

H 10.03956 2.98489 14.42290

C 8.98519 5.54396 13.90403

H 8.29886 4.94032 14.49671

H 9.56845 6.15142 14.60365

H 8.40326 6.22001 13.27256

C 6.84960 2.91737 12.82755

C 7.39480 2.32649 14.13725

H 6.56190 2.02682 14.78212

H 8.00728 1.44025 13.94103

H 8.00096 3.03941 14.69662

C 6.00751 4.17281 13.10517

H 5.57276 4.53811 12.17147

H 5.19956 3.92325 13.80257

H 6.58786 4.98097 13.54321

C 5.87862 1.87906 12.23803

H 6.37925 0.93198 12.01160

H 5.08883 1.67432 12.96740

H 5.40009 2.25575 11.32956

C 9.46054 2.55562 8.39168

C 8.21494 2.66579 7.50405

H 7.83753 3.69066 7.50578

H 8.45626 2.37847 6.47525

H 7.41060 2.01204 7.85435

C 10.57000 3.44599 7.82453

H 11.50812 3.31469 8.37344

H 10.76007 3.18978 6.77749

H 10.28776 4.50094 7.87690

C 9.94172 1.09451 8.42809

H 9.16006 0.43361 8.81598

H 10.20722 0.75562 7.42072

H 10.82351 0.98893 9.06831

N 6.43746 4.94973 9.96529

N 8.74020 6.21802 9.95640

Fe 7.08572 6.48506 9.58308

Fe 8.08592 4.68878 10.35386

96

Cp2Fe2N2, 2S+1 = 3, with dispersion

N 6.33441 4.89833 9.63874

Fe 6.85637 6.52274 9.64521

C 5.71300 7.36715 8.02465

C 6.99109 8.07141 8.19189

C 6.96776 8.64457 9.49606

H 7.79288 9.16812 9.94999

C 5.79111 8.25702 10.18451

C 5.01072 7.52217 9.24554

H 4.06992 7.04999 9.47643

C 5.06155 6.62326 6.85912

C 3.94529 5.70294 7.38703

H 4.33933 5.01306 8.13935

H 3.53723 5.12161 6.55439

H 3.11359 6.26789 7.81875

C 4.41305 7.66404 5.92666

H 3.68091 8.26466 6.47595

H 3.89081 7.15678 5.10794

H 5.14553 8.34596 5.49148

C 6.01666 5.71988 6.07413

H 6.79547 6.27233 5.55008

H 5.44956 5.16578 5.31907

H 6.48561 4.99675 6.74676

C 8.20045 8.26990 7.27230

C 7.76830 8.84555 5.91498

H 8.65487 9.10269 5.32601

H 7.17528 9.75634 6.04785

H 7.18013 8.14035 5.32815

C 9.00879 6.97381 7.08532

H 9.39745 6.63954 8.05057

H 9.85017 7.16137 6.40834

H 8.41539 6.16267 6.66944

C 9.16687 9.28829 7.90220

H 8.68458 10.25541 8.07798

H 10.00726 9.45150 7.22059

H 9.57631 8.91790 8.84635

C 5.40414 8.62285 11.59800

C 6.64329 8.65131 12.50072

H 7.14735 7.68129 12.49208

H 6.35307 8.89404 13.52815

H 7.36467 9.40599 12.17293

C 4.39961 7.60813 12.15456

H 3.45869 7.62193 11.59549

H 4.16723 7.84438 13.19772

H 4.80829 6.59471 12.11165

C 4.75817 10.01950 11.56006

H 5.46126 10.76537 11.17597

H 4.45153 10.32435 12.56654

H 3.87260 10.02459 10.91638

N 8.37020 6.32029 10.40770

Fe 7.89009 4.62423 10.42709

C 9.51115 3.76014 11.69928

C 8.26816 3.15967 12.13985

C 7.72272 2.48869 11.00541

H 6.76538 1.99342 10.98178

C 8.59747 2.59067 9.88760

C 9.69369 3.36155 10.32841

H 10.52749 3.66144 9.71319

C 10.60591 4.55870 12.40998

C 11.50945 5.25036 11.37204

H 10.91677 5.89461 10.71615

H 12.24542 5.87002 11.89416

H 12.06909 4.53214 10.76559

C 11.48211 3.57922 13.21267

H 11.90823 2.81958 12.54920

H 12.30919 4.11739 13.68965

H 10.91859 3.06244 13.99127

C 10.07893 5.68180 13.31180

H 9.49701 5.31766 14.15742

H 10.92529 6.24328 13.72197

H 9.46222 6.36897 12.72505

C 7.46708 3.25533 13.44189

C 8.31834 2.91426 14.67280

H 7.68857 2.91047 15.56913

H 8.76374 1.91966 14.56822

H 9.12381 3.62714 14.84280

C 6.82383 4.64758 13.58006

H 6.15485 4.83078 12.73201

H 6.22777 4.70586 14.49830

H 7.55962 5.45004 13.59998

C 6.31025 2.24006 13.42044

H 6.67220 1.21843 13.26686

H 5.78480 2.27219 14.38014

H 5.57880 2.47289 12.64119

C 8.39534 1.99846 8.50984

C 6.90743 1.73509 8.25203

H 6.32590 2.65435 8.37435

H 6.76472 1.35707 7.23418

H 6.50970 0.98219 8.94044

C 8.91808 2.96944 7.44309

H 9.98964 3.16076 7.55633

H 8.75786 2.55812 6.44081

H 8.39509 3.92829 7.50996

C 9.17770 0.67656 8.42886

H 8.82417 -0.03085 9.18582

H 9.05244 0.21424 7.44308

H 10.24659 0.84391 8.59614

1. **References**

[1] Gaussian 16, Revisions B.01v and B.01 (Gaussian Inc., 2016).

[2] A. D. Becke, “Density-functional thermochemistry. III. The role of exact exchange” *J. Chem. Phys.* **1993**, *98*, 5648–5652.

[3] K. Burke, J. P. Perdew, Y. Wang, Y. in *Electronic Density Functional Theory: Recent Progress and New Directions* (Eds.: J. F. Dobson, G. Vignale, P. D. Mukunda), Springer, New York, NY, **1997**, pp. 81-111.

[4] M. Dolg, U. Wedig, H. Stoll, H. Preuss, “Energy‐adjusted ab initio pseudopotentials for the first row transition elements” *J. Chem. Phys.* **1987**, *86*, 866–872.

[5] A. W. Ehlers, M. Böhme, S. Dapprich, A. Gobbi, A. Höllwarth, V. Jonas, K. F. Köhler, R. Stegmann, A. Veldkamp, G. Frenking, “A set of f-polarization functions for pseudo-potential basis sets of the transition metals Sc-Cu, Y-Ag and La-Au”, *Chem. Phys. Lett.* **1993**, *208*, 111–114.

[6] P. C. Hariharan, J. A. Pople, “The influence of polarization functions on molecular orbital hydrogenation energies”, *Theor. Chem. Acc.* **1973**, *28*, 213–222.

[7] W. J. Hehre, R. Ditchfield, J. A. Pople, “Self-consistent molecular orbital methods. XII. Further extensions of Gaussian-type basis sets for use in molecular orbital studies of organic molecules” *J. Chem. Phys.* **1972**, *56*, 2257–2261.

[8] A. E. Reed, L. A. Curtiss, F. Weinhold, “Intermolecular interactions from a natural bond orbital, donor–acceptor viewpoint”, *Chem. Rev.* **1988**, *88*, 899–926.

[9] S. Grimme, S. Ehrlich, L. J. Goerigk, “Effect of the damping function in dispersion corrected density functional theory”, *Comp. Chem.* **2011**, *32*, 1456-1465.
